# Global, regional and national burden of bladder cancer and its attributable risk factors in 204 countries and territories, 1990–2019: a systematic analysis for the Global Burden of Disease study 2019

**DOI:** 10.1136/bmjgh-2020-004128

**Published:** 2021-11-29

**Authors:** Saeid Safiri, Ali-Asghar Kolahi, Mohsen Naghavi, Global Burden of Disease Bladder Cancer Collaborators, Saeid Safiri

**Affiliations:** 1 Aging Research Institute, Tabriz University of Medical Sciences, Tabriz, Iran (the Islamic Republic of); 2 Social Determinants of Health Research Center, Shahid Beheshti University of Medical Sciences, Tehran, Iran; 3 Institute for Health Metrics and Evaluation, University of Washington, Seattle, Washington, USA

**Keywords:** cancer, epidemiology

## Abstract

**Introduction:**

The current study determined the level and trends associated with the incidence, death and disability rates for bladder cancer and its attributable risk factors in 204 countries and territories, from 1990 to 2019, by age, sex and sociodemographic index (SDI; a composite measure of sociodemographic factors).

**Methods:**

Various data sources from different countries, including vital registration and cancer registries were used to generate estimates. Mortality data and incidence data transformed to mortality estimates using the mortality to incidence ratio (MIR) were used in a cause of death ensemble model to estimate mortality. Mortality estimates were divided by the MIR to produce incidence estimates. Prevalence was calculated using incidence and MIR-based survival estimates. Age-specific mortality and standardised life expectancy were used to estimate years of life lost (YLLs). Prevalence was multiplied by disability weights to estimate years lived with disability (YLDs), while disability-adjusted life years (DALYs) are the sum of the YLLs and YLDs. All estimates were presented as counts and age-standardised rates per 100 000 population.

**Results:**

Globally, there were 524 000 bladder cancer incident cases (95% uncertainty interval 476 000 to 569 000) and 229 000 bladder cancer deaths (211 000 to 243 000) in 2019. Age-standardised death rate decreased by 15.7% (8.6 to 21.0), during the period 1990–2019. Bladder cancer accounted for 4.39 million (4.09 to 4.70) DALYs in 2019, and the age-standardised DALY rate decreased significantly by 18.6% (11.2 to 24.3) during the period 1990–2019. In 2019, Monaco had the highest age-standardised incidence rate (31.9 cases (23.3 to 56.9) per 100 000), while Lebanon had the highest age-standardised death rate (10.4 (8.1 to 13.7)). Cabo Verde had the highest increase in age-standardised incidence (284.2% (214.1 to 362.8)) and death rates (190.3% (139.3 to 251.1)) between 1990 and 2019. In 2019, the global age-standardised incidence and death rates were higher among males than females, across all age groups and peaked in the 95+ age group. Globally, 36.8% (28.5 to 44.0) of bladder cancer DALYs were attributable to smoking, more so in males than females (43.7% (34.0 to 51.8) vs 15.2% (10.9 to 19.4)). In addition, 9.1% (1.9 to 19.6) of the DALYs were attributable to elevated fasting plasma glucose (FPG) (males 9.3% (1.6 to 20.9); females 8.4% (1.6 to 19.1)).

**Conclusions:**

There was considerable variation in the burden of bladder cancer between countries during the period 1990–2019. Although there was a clear global decrease in the age-standardised death, and DALY rates, some countries experienced an increase in these rates. National policy makers should learn from these differences, and allocate resources for preventative measures, based on their country-specific estimates. In addition, smoking and elevated FPG play an important role in the burden of bladder cancer and need to be addressed with prevention programmes.

**Key questions**

**What is already known?**

- A small number of studies have investigated the global burden of bladder cancer over the last 20 years using information from the Global Cancer Incidence, Mortality and Prevalence project (GLOBOCAN).
- The most recent iteration of GLOBOCAN was 2020, which did not include DALYs, an important health metric to capture morbidity outcomes as well as mortality.

**Key questions**

**What are the new findings?**

- This research reports the most up-to-date estimates on the level and trends in the incidence, mortality, and DALYs for bladder cancer and its attributable by age, sex and socio-demographic index (SDI; a composite measure of socio-demographic factors) risk factors from 204 countries and territories from 1990 to 2019.
- Globally, there were 524,000 bladder cancer incident cases (95% UI: 476,000 to 569,000), 229,000 bladder cancer deaths (211,000 to 243,000), and 4.39 million (4.09 to 4.70) DALYs attributable to bladder cancer in 2019.
- The global age-standardised incidence and death rates were higher among males than females.
- Globally, 36.8% (28.5 to 44.0) and 9.1% (1.9 to 19.6) of bladder cancer DALYs were attributable to smoking and elevated fasting plasma glucose, respectively.

**What do the new findings imply?**

- Globally bladder cancer continues to be a considerable public health challenge. Although the rates of bladder cancer decreased globally, there were several some countries which registered increases.
- Additional research is needed into the reasons for the increases in these countries to guide new measures and to facilitate the early detection and treatment of this disease.
- Preventive measures should be developed to reduce exposure to risk factors such as high fasting plasma glucose and smoking, as well as placing higher taxes on smoked tobacco.

## Introduction

Cancers remain one of the leading causes of mortality, with 9.6 million deaths globally in 2017.^1^ Bladder cancer, as one of the important urological cancers, caused 196.5 thousand deaths and was categorised as the 9th and 19th leading cause of cancer-related deaths for males and females, respectively.^1 2^ A study on the economic cost of bladder cancer in the European Union (EU) found that this form of cancer cost the EU €4.9 billion in 2012, with healthcare accounting for €2.9 billion (59%), which represented 5% of the total healthcare costs for cancer. In 2012, bladder cancer accounted for 3% of all cancer costs in the EU (€143 billion), which represented an annual healthcare cost of €57 per 10 EU citizens. However, there were large variations in the cost by country, with the lowest cost being found in Bulgaria (€8 for every 10 citizens) and the highest in Luxembourg (€93).^3^ Understanding the variations and trends in the incidence, mortality, and disability-adjusted life years (DALYs) for bladder cancer allows national-level policy makers to make appropriate, evidence-based decisions in their countries, to evaluate the effectiveness of their interventions and to more efficiently manage its relevant costs.

Only a few previous studies have reported the global and regional rates of bladder cancer. However, these studies have only reported its burden at the global or regional level,^1^ ^4 5^ ^5^ or have not reported country-specific estimates using data collected after GLOBOCAN (Global Cancer Incidence, Mortality and Prevalence) 2012.^6 7^ However, a recent paper reported an update on the global epidemiology of this cancer using GLOBOCAN 2018 data, but comparing between countries is problematic, since the rates were not age-standardised.^8^ The most recent study om the burden of cancers is GLOBOCAN 2020 which still has the mentioned limitations.^9^ In addition, calculating the contribution the individual risk factors make to the burden of bladder cancer allows an understanding of the degree to which the burden of bladder cancer could be reduced by eliminating each risk factor and also provides information vital for prevention programmes. The attributable burden has not been estimated in previous research.

Therefore, considering the aforementioned issues, the present study supersedes the Global Burden of Disease (GBD) 2016 bladder cancer paper^10^ as new data sources have been added and new methods have been applied in GBD 2019.^11^ More specifically, the present article provides the most up-to-date estimates on the global, regional and national incidence, mortality, and DALYs for bladder cancer and its attributable risk factors in terms of counts and age-standardised rates for 204 countries and territories from 1990 to 2019 by age, sex and sociodemographic index (SDI).

## Methods

### Overview

The Global Burden of Diseases, Injuries and Risk Factors (GBD) study is a comprehensive effort to estimate burden due to 369 diseases and injuries, and 87 risk factors across 204 countries and territories, 21 regions and 7 super-regions. GBD 2019 is the latest round in which the estimates were not only updated for 2019, but also previous estimates (1990–2017) have been strengthened using additional data sources and new estimation methods. The main features of GBD 2019, and its general methodology, can be found in previously published papers.^11–13^ In the GBD 2019 study, 30 cancer groups, including bladder cancer, were estimated.^11^ The 95% uncertainty intervals (UIs) have been calculated for all the estimates and the rates were standardised based on the GBD standard population and reported per 100 000 population. The methods for propagating the UIs were similar to those used in previous GBD iterations. One thousand draws were taken at each computational step and final estimates were computed using mean estimates across the draws. 95% UIs were presented as the 25th and 975th ordered values across all 1000 draws.

This study is compliant with the Guidelines for Accurate and Transparent Health Estimates Reporting.^14^ This manuscript was produced as part of the GBD Collaborator Network and in accordance with the GBD Protocol.

### Estimation framework

All cancers coded C67–C67.9, D09.0, D30.3, D41.4-D41.8 and D49.4 in the International Classification of Diseases 10 were considered as bladder cancer.^11^ Six sequelae with different disability weights (DWs) were defined as bladder cancer (online supplemental table 1).^11^ The GBD 2013 European Disability Weights Measurement Study and GBD 2010 Disability Weights Measurement Study were used as sources of the DW values. More details have been reported elsewhere.^11 15^ The following data sources were used to estimate the non-fatal and fatal burden of bladder cancer: vital registration (21 734 site-years), vital registration-sample (825 site-years) and cancer registries (5 146 site-years).^11 12^ A site-year is a unique combination of the location and calendar year and is defined as a country or other subnational geographical unit contributing data in a given year.

10.1136/bmjgh-2020-004128.supp1 Supplementary data

### Mortality estimation

The availability of cancer mortality data was generally lower than for incidence data. Mortality to incidence ratios (MIRs) were obtained from linear-step mixed effect models using the locations where the incidence and mortality data were both provided for the same year. Age, sex and the healthcare access and quality index were also adjusted in the model and then smoothed across space and time using spatiotemporal Gaussian processes regression.^11 12^

Initially, mortality estimates were obtained by multiplying the corresponding incidence estimate with the MIR. These estimated mortalities, along with the observed deaths from vital registration systems and verbal autopsies, were used as inputs for the cause of death ensemble model (CODEm).^11^ This approach evaluates the predictive validity of various models to provide the highest model fit using all available data and covariates. The covariates used in CODEm are available in online supplemental table 2. The CoDCorrect algorithm was used to adjust the sum of predicted single-cause mortalities in an age-sex-location-year group to be consistent with the results from all-cause mortality estimation.^11^

10.1136/bmjgh-2020-004128.supp2 Supplementary data

### Incidence, prevalence and disability estimation

The final mortality estimates from CODEm were divided by the MIR to obtain the final incidence estimates. Ten-year prevalence of bladder cancer was calculated through modelling the survival for each country using MIRs and divided into five sequalae (online supplemental table 1). Sequelae-specific years lived with disability (YLDs) were calculated as the product of sequelae-specific prevalence and corresponding DWs. In addition, procedure-related YLDs, due to incontinence from cystectomy, were calculated for bladder cancer and were added to the previous sequelae-specific YLDs (online supplemental table 1). To estimate procedure-related disability for bladder cancer, the procedure proportions (proportion of bladder cancer population that underwent cystectomy) from hospital data were used as the input for a proportion model in DisMod-MR 2.1 to estimate the proportions for all locations, by age, year and sex.^11^ The years of life lost (YLLs) were computed by multiplying the estimated number of deaths by age with a standard life expectancy at that age. DALYs were obtained by summing YLDs and YLLs.

The current study examined the association of bladder cancer incidence, mortality and DALYs with SDI for each country using smoothing splines models.^16^ SDI is a composite indicator of lag-dependent income per capita, average years of schooling for the population older than 15 years of age, and total fertility rate under the age of 25. It ranges from 0 (lowest average income and education; highest fertility) to 1 (highest average income and education; lowest fertility).^11^ The world maps for age-standardised incidence, prevalence and DALYs were generated using R software, V.3.5.2.

### Risk factors

Systematic reviews were conducted in previous GBD rounds to assess possible risks associated with bladder cancer; inclusion criteria were based on World Cancer Research Fund criteria for convincing or probable evidence. Through this evaluation, smoking^17^ and high fasting plasma glucose (FPG)^18^ were identified as risk factors for bladder cancer.

To estimate risk-attributable burden, we first calculated the population attributable fraction (PAF)—the proportion of all bladder cancer cases attributable to each risk factor—using estimates of exposure distribution and levels and relative risks at different exposures (see Murray et al.^12^ for detailed methods). We then multiplied age-sex-year-location-specific PAFs for each risk factor by the number of bladder cancer DALYs in that population to get risk-attributable DALYs due to smoking and high FPG. Current smokers were defined as individuals who currently use any smoked tobacco product on a daily or occasional basis. Former smokers were defined as individuals who quit using all smoked tobacco products for at least 6 months, where available, or according to the definition used by the survey. The reference definition used for diabetes was: FPG >126 mg/dL (7 mmol/L) or on treatment.

## Results

### Global level

There were 524 000 bladder cancer cases (95% UI 476 000 to 569 000) in 2019, with an age-standardised rate of 6.5 (5.9 to 7.1) per 100 000, which increased, by 4% (−4.3 to 13.5), between 1990 and 2019 (online supplemental table 3). It was found that this cancer also globally accounted for 229 000 deaths (211 000 to 243 000) in 2019, with an age-standardised death rate of 2.9 (2.7 to 3.1) per 100 000, which decreased significantly by 15.7% (8.6 to 21.0) during the period 1990–2019 (online supplemental table 4). Globally bladder cancer also accounted for 4.39 million DALYs (4.09 to 4.70) in 2019, with an age-standardised rate of 54.2 (50.4 to 58) per 100 000 which significantly decreased by 18.6% (11.2 to 24.3) (online supplemental table 5).

10.1136/bmjgh-2020-004128.supp3 Supplementary data

10.1136/bmjgh-2020-004128.supp4 Supplementary data

10.1136/bmjgh-2020-004128.supp5 Supplementary data

### Regional level

**Figure 1:**
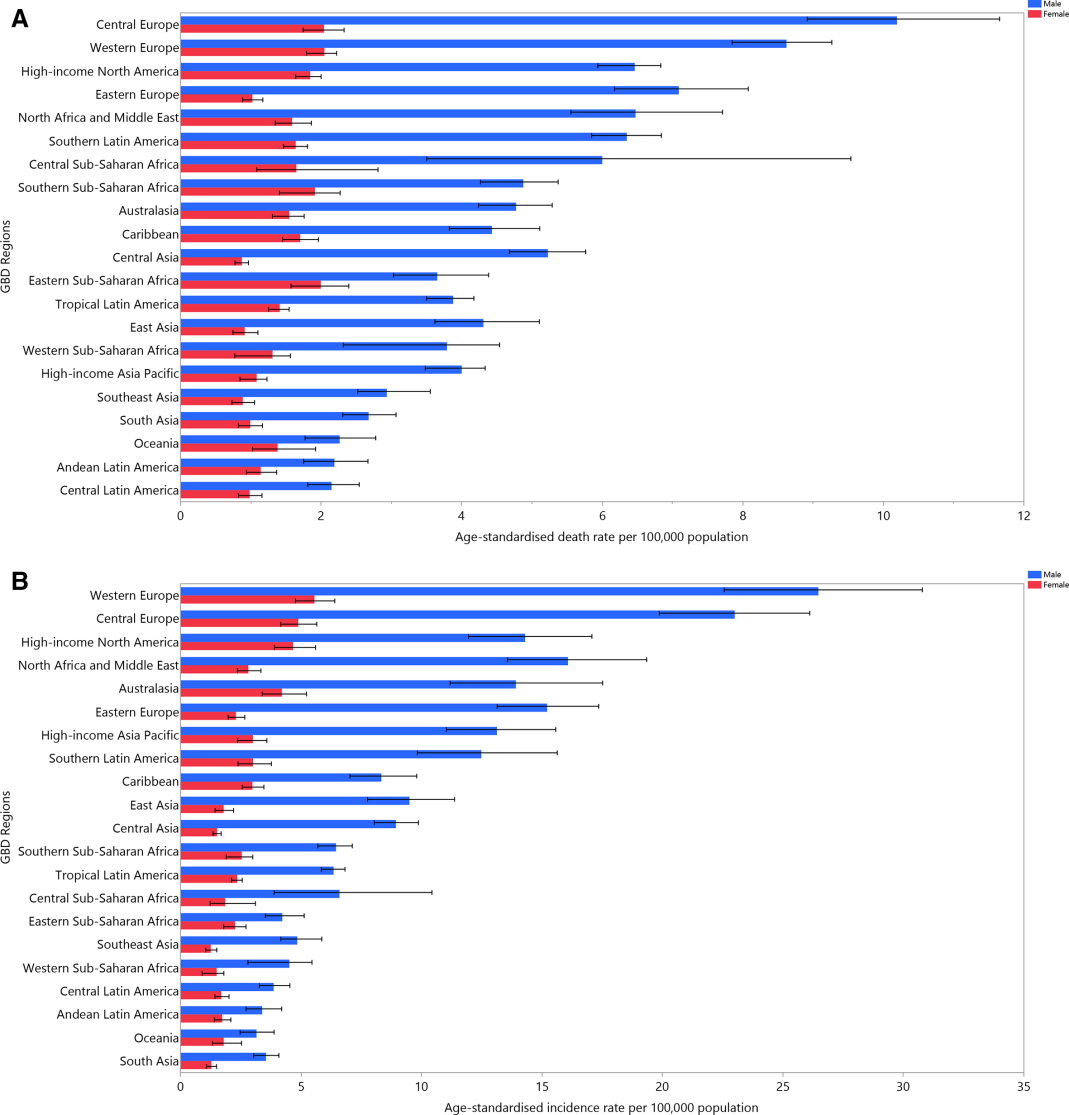
The age-standardised incidence (A) and death (B) rates of bladder cancer per 100 000 population in 2019 for the 21 Global Burden of Disease regions, by sex.

The age-standardised incidence rates of bladder cancer per 100 000 in 2019 were highest in western Europe (14.9 (95% UI 12.8 to 17.3)), central Europe (12.6 (11 to 14.3)) and north Africa and the Middle East (9.6 (8.1 to 11.4)). In contrast, south Asia (2.4 (2.1 to 2.7)), Oceania (2.5 (2 to 3.1)) and Andean Latin America (2.5 (2.1 to 3.1)) showed the lowest age-standardised incidence rates (online supplemental table 3). The age-standardised death rates of bladder cancer per 100 000 in 2019 were highest in central Europe (5.3 (4.7 to 6)), western Europe (4.8 (4.3 to 5.1)) and north Africa and the Middle East (4.1 (3.5 to 4.8)), whereas central Latin America (1.5 (1.3 to 1.8)), Andean Latin America (1.6 (1.3 to 2)) and southeast Asia (1.8 (1.5 to 2)) had the lowest age-standardised death rates (online supplemental table 4). The regional-level age-standardised incidence and death estimates per 100 000 for all GBD regions are presented, by sex, in figure 1A, B.

**Figure 2:**
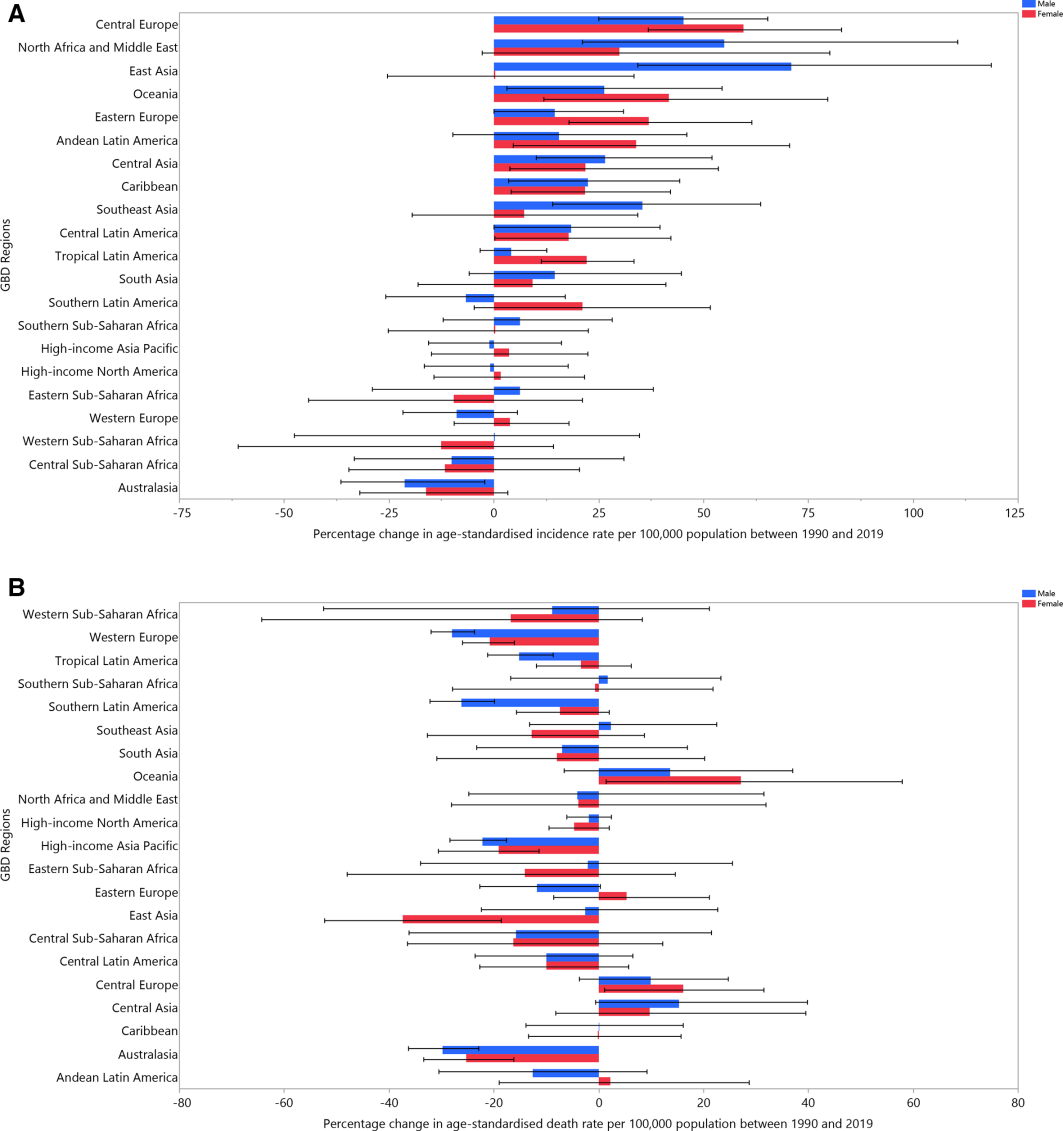
The percentage change in age-standardised incidence (A) and death (B) rates of bladder cancer from 1990 to 2019 for the 21 Global Burden of Disease regions by sex.

Although globally age-standardised incidence rate did not significantly change, there were substantial increases in some GBD regions, such as east Asia (55.6% (26.1 to 95.8)), north Africa and the Middle East (52.5% (21.3 to 107.1)) and central Europe (50.3% (30.3 to 70.7)) (online supplemental table 3). The age-standardised death rate also significantly decreased globally, but significantly increased in central Asia (17.9% (1.8 to 42.7)) (online supplemental table 4). Regional-level percentage changes in age-standardised incidence and death rates, due to bladder cancer, are presented by sex in figure 2A, B.

**Figure 3:**
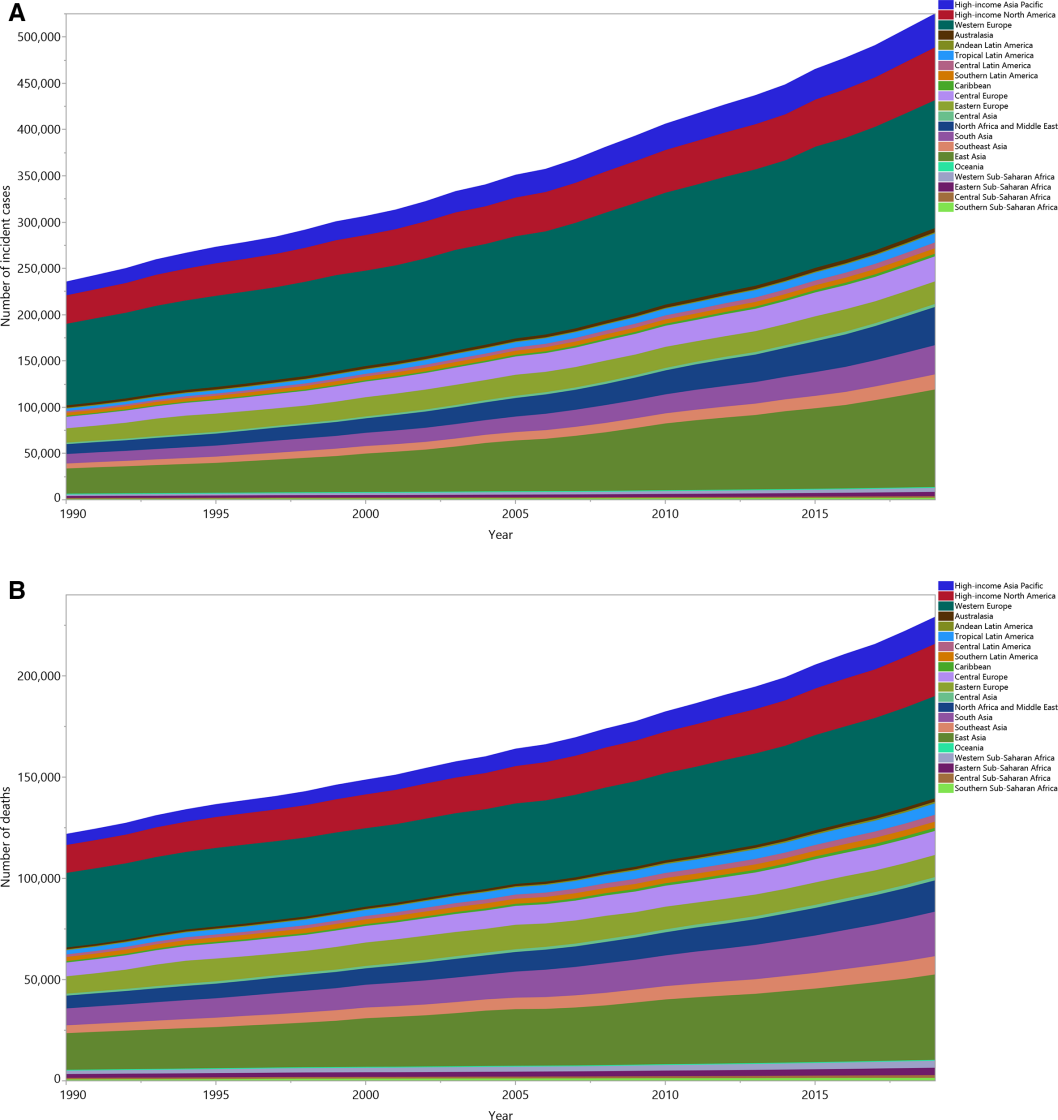
Number of incident cases (A) and deaths (B) due to bladder cancer from 1990 to 2019 for the 21 Global Burden of Disease regions.

The number of incident cases and deaths due to bladder cancer increased from 1990 to 2019 (incident cases from 235 000 (225 000 to 243 000) to 524 000 (476 000 to 569 000) and deaths from 122 000 (115 000 to 127 000) to 229 000 (211 000 to 243 000)), but the contributions of the individual GBD regions differed during this time-period (figure 3A, B). In 2019, western Europe and east Asia together accounted for nearly half of all incident and death cases of bladder cancer.

### National level

**Figure 4:**
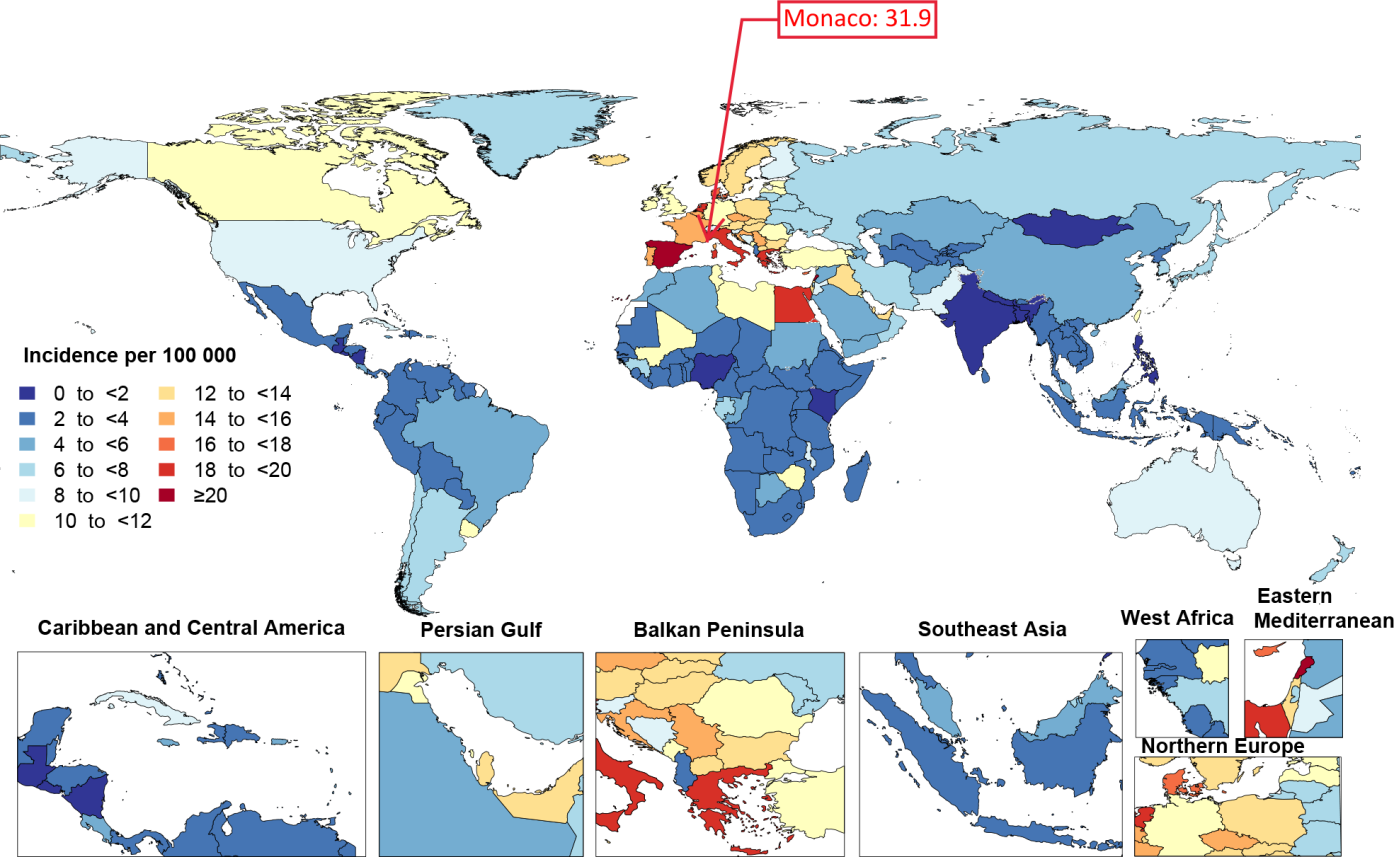
Age-standardised incidence rate of bladder cancer per 100 000 population by location for both sexes, 2019.

**Figure 5:**
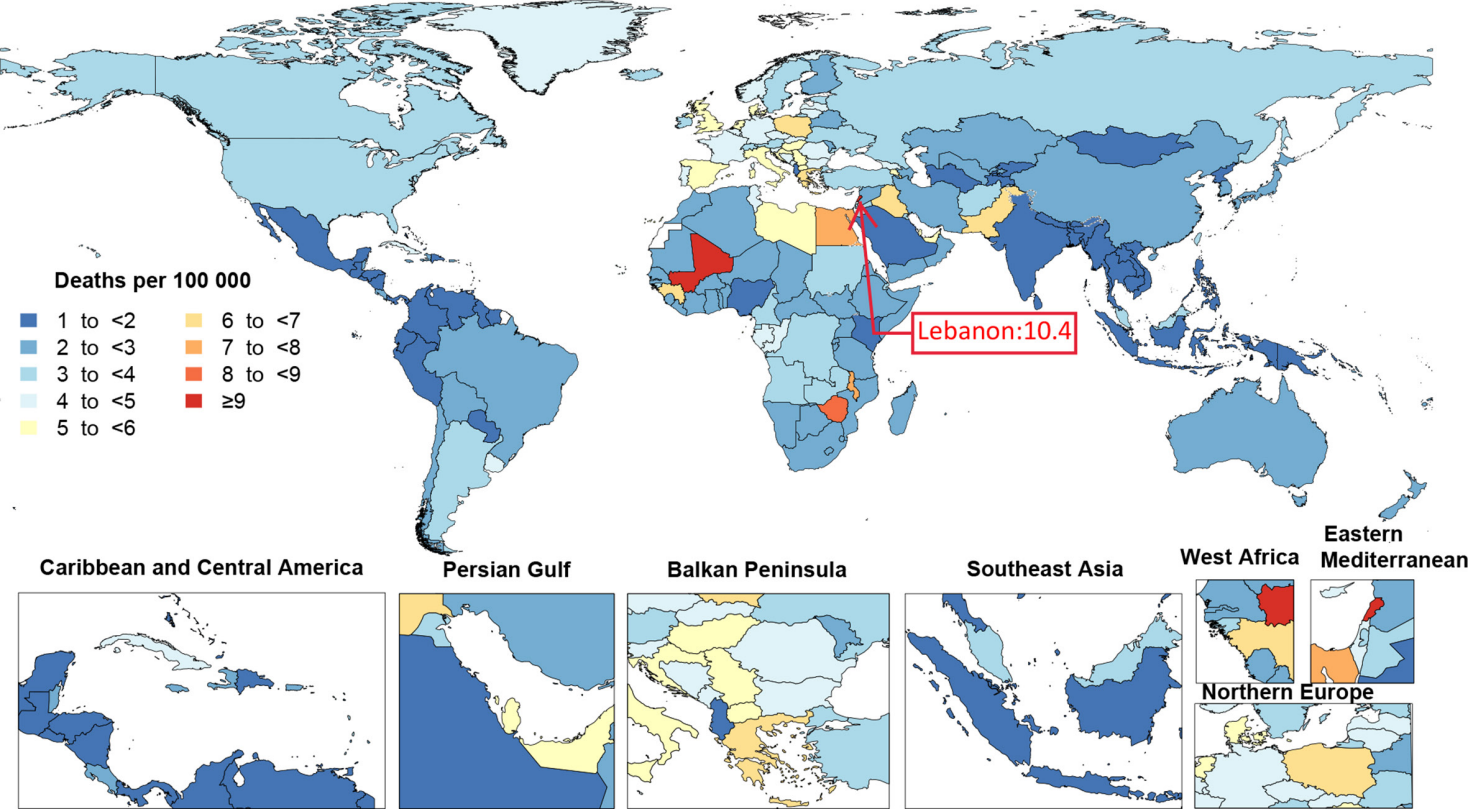
Age-standardised death rates of bladder cancer per 100 000 population by location for both sexes, 2019.

In 2019, the age-standardised incidence rates of bladder cancer ranged from 1.4 to 31.9 per 100 000 population among countries. Monaco (31.9 (23.3 to 56.9)), Lebanon (30.2 (23 to 40.4)) and San Marino (25.3 (18.9 to 33.9)) had the three highest age-standardised incidence rates per 100 000 and Nigeria (1.4 (1.1 to 1.7)), Guatemala (1.5 (1.2 to 1.9)) and Bangladesh (1.6 (1.1 to 2.1)) had the lowest (figure 4 and online supplemental table 3). The age-standardised death rate due to bladder cancer, in 2019, also varied between the countries (from 1 to 10.4 per 100 000 population). Lebanon (10.4 (8.1 to 13.7)), Mali (10.1 (4.4 to 13.5)) and Monaco (9.4 (6.9 to 16.9)) had the three highest age-standardised death rates per 100 000, whereas Palau (1 (0.8 to 1.3)), Albania (1.1 (0.8 to 1.4)) and El Salvador (1.1 (0.9 to 1.4)) had the lowest (figure 5 and online supplemental table 4).

The percentage change in age-standardised incidence rates from 1990 to 2019 differed substantially between countries. Cabo Verde (284.2% (214.1 to 362.8)), Qatar (126.5% (51.5 to 243.7)) and Northern Mariana Islands (119.8% (71.9 to 169.9)) showed the largest significant increases, while Liberia (−36.1% (−60.5 to −3.3)), Togo (−34.2% (−67.9 to −5.2)) and Burkina Faso (−34.1% (−71.1 to −3.6)) had the largest significant decreases (online supplemental table 3). The percentage change in age-standardised death rates, due to bladder cancer (from 1990 to 2019), also differed between countries. The largest significant increases were seen in Cabo Verde (190.3% (139.3 to 251.1)), Northern Mariana Islands (81.8% (45.3 to 120.9)) and Uzbekistan (64.7% (11.2 to 142)). In contrast, the largest significant decreases during this period were found in Singapore (−44.9% (−53.5 to −35.8)), Thailand (−42.1% (−57.7 to −22.7)) and Sierra Leone (−42.1% (−78.8 to −7)) (online supplemental table 4).

### Age and sex patterns

**Figure 6:**
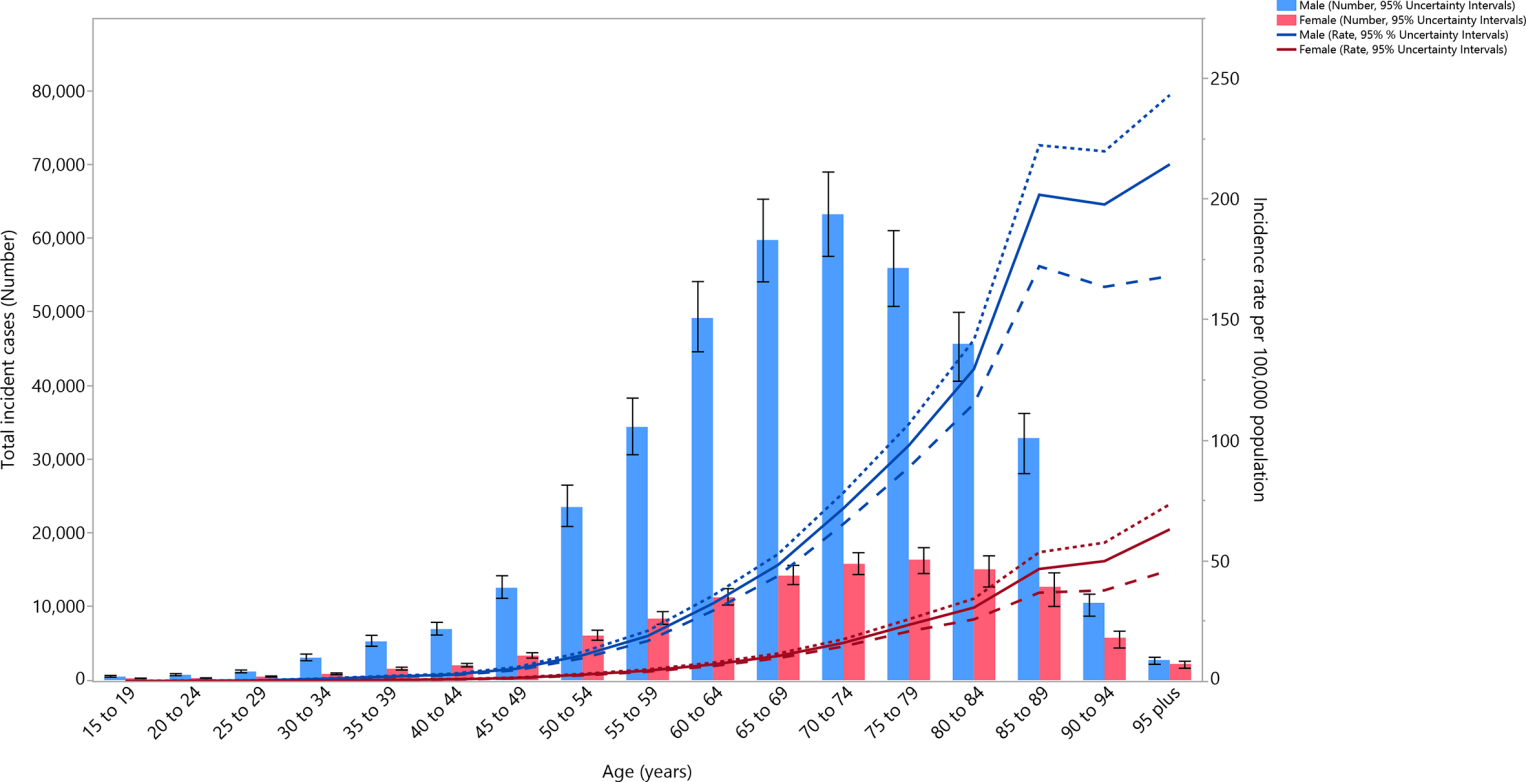
Global number of incident cases and age-standardised incidence rates of bladder cancer per 100 000 population by age and sex, 2019; dotted and dashed lines indicate 95% upper and lower UIs, respectively.

In 2019, the global incidence rates of bladder cancer per 100 000 were higher among males than females, across all age groups. The incidence rates increased with population ageing and peaked at the 95+ age group among both males (214.4 (168.1 to 243.2)) and females (63 (46.1 to 73.4)). The number of incident cases also peaked at 70–74 and 75–79 years old in males and females, respectively (figure 6). The global death rate per 100 000 in 2019 also peaked in the 95+ group in males (245.9 (192.6 to 277.8)) and females (75.4 (55.4 to 87.3)). However, the number of deaths was highest in the 80–84 age group in both males and females (online supplemental figure 1). The DALY rates per 100 000 were higher in males than females across all age groups and peaked at 90–94 years for males (1334.8 (1112 to 1466.8)) and 95+ years for females (401 (297.5 to 462.9)). The number of DALYs peaked at 65–69 and 70–74 years in males and females, respectively (online supplemental figure 2). The DALYs were mainly composed of YLLs, whose rate peaked in the 90–94 age group. The number of YLLs and YLDs were highest in the 70–74 age group (online supplemental figure 3).

10.1136/bmjgh-2020-004128.supp6 Supplementary data

10.1136/bmjgh-2020-004128.supp7 Supplementary data

10.1136/bmjgh-2020-004128.supp8 Supplementary data

### Burden of bladder cancer by SDI

**Figure 7:**
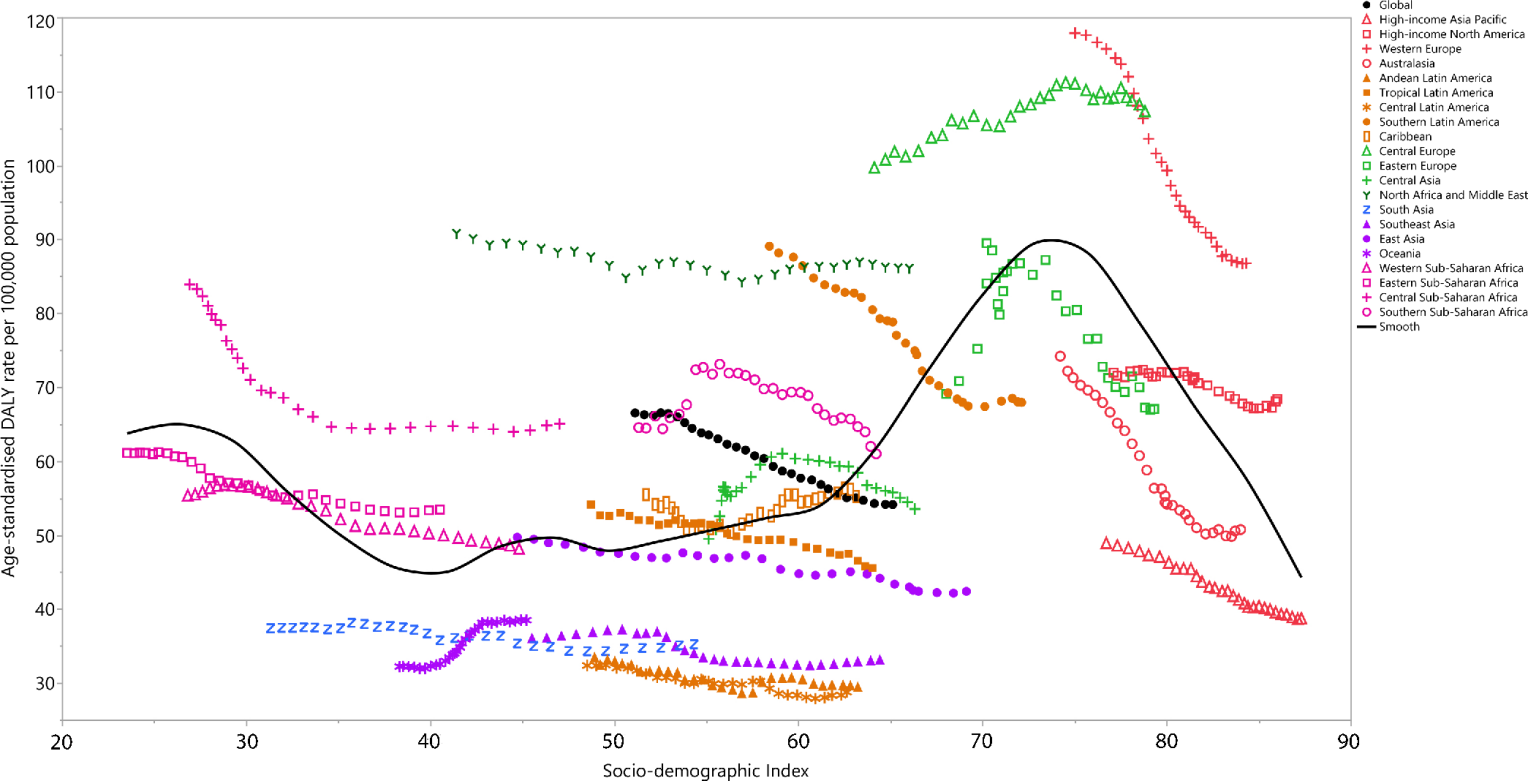
Age-standardised DALY rates of bladder cancer per 100 000 population for the 21 Global Burden of Disease regions by sociodemographic index (SDI), 1990–2019; expected values based on SDI and disease rates in all locations are shown as the black line. DALY, disability-adjusted life year.

There was a non-linear association between the age-standardised DALY rate per 100 000 and the SDI of the GBD regions. The global age-standardised DALY rate was higher than expected up until 2013, but was lower than expected during the period 2014–2019. Despite the decreasing trend of age-standardised DALY rate in the high-income GBD super-region, western Europe and high-income North America still had DALY rates higher than expected, based on SDI. In the Latin America super-region, all regions had lower than expected age-standardised DALY rates in 2019. The age-standardised DALY rate was higher than expected from 1990 to 2019 in central Europe. Both eastern Europe and central Asia showed a downward trend in the last few years of the measurement period and their rates were lower than expected in 2019. North Africa and the Middle East had higher than expected age-standardised DALY rates, based on their SDI during the period 1990–2019. In contrast, south Asia, southeast Asia and Oceania had lower than expected age-standardised DALY rates during the measurement period. All regions in sub-Saharan Africa had higher than expected age-standardised DALY rates in the most recent years (figure 7).

**Figure 8:**
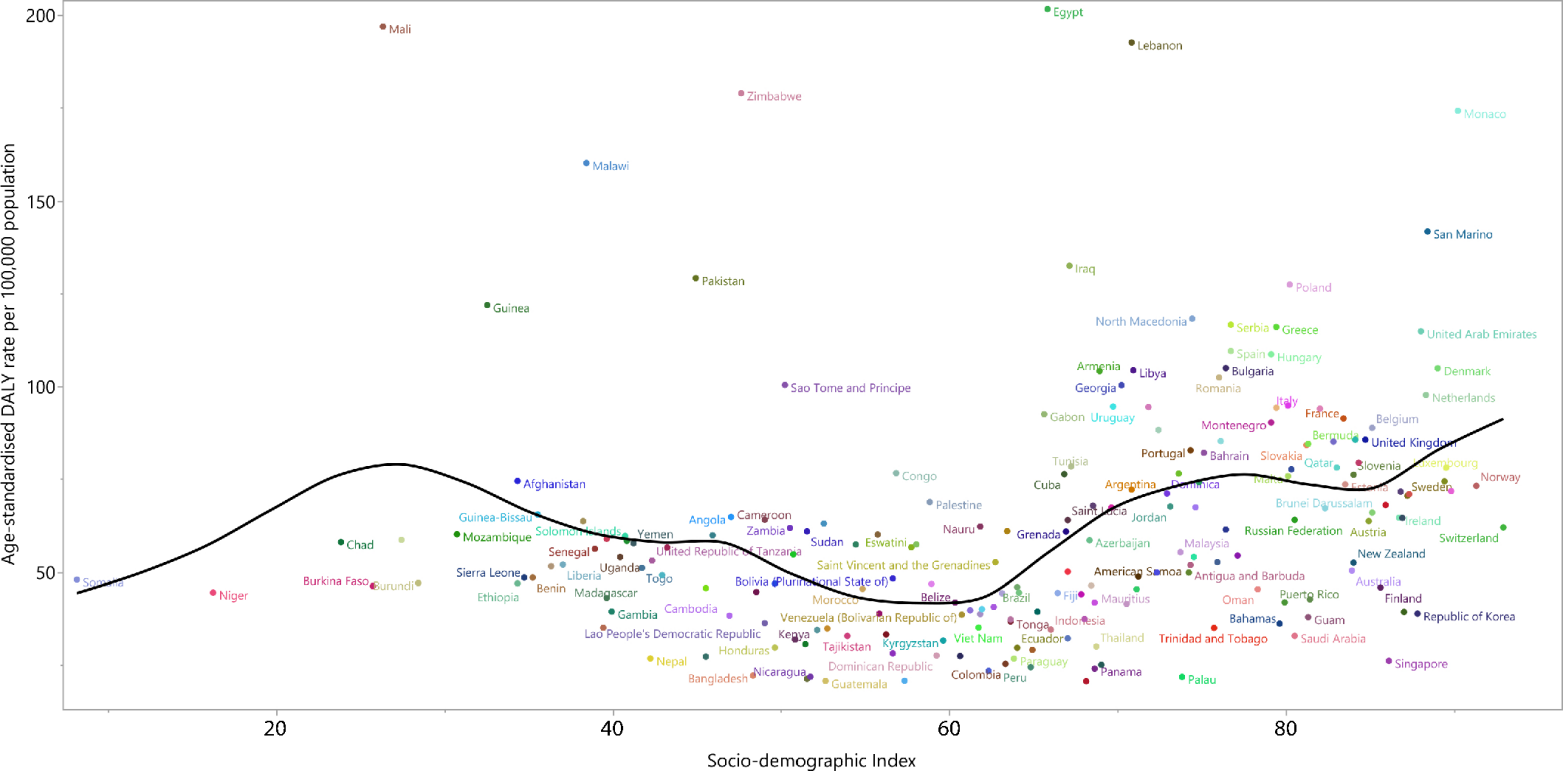
Age-standardised DALY rates of bladder cancer per 100 000 population for the 204 countries and territories and sociodemographic Index, 2019; expected values are shown as the black line. DALY, disability-adjusted life year.

National-level analysis in 2019 found there was a non-linear association between age-standardised DALY rates of bladder cancer per 100 000 and each country’s SDI. There were countries with much higher than expected levels of age-standardised DALY rates, based on SDI, in both higher and lower SDI regions. Egypt, Lebanon, Mali, Monaco, Zimbabwe, Malawi, Pakistan and many other countries had much higher than expected levels of age-standardised DALY rates of bladder cancer in 2019. In contrast, there were some countries, such as Singapore, Republic of Korea, Finland, Bangladesh, Peru and so on, which had much lower than expected age-standardised DALY rates of bladder cancer, based on SDI (figure 8).

### Risk factors

**Figure 9:**
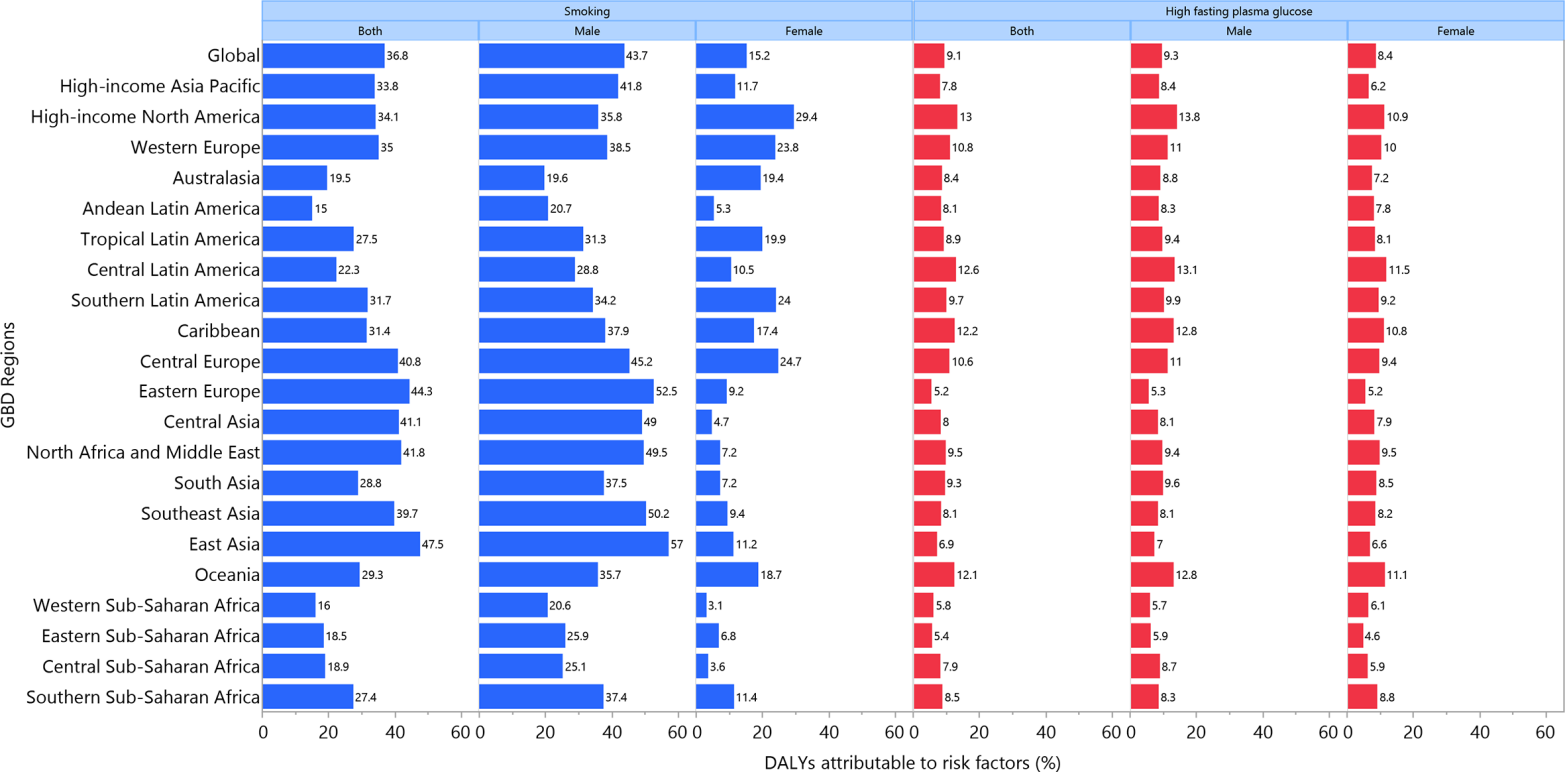
Percentage of age-standardised DALYs due to bladder cancer attributable to risk factors for 21 Global Burden of Disease regions, by sex, 2019. DALY, disability-adjusted life year.

**Figure 10:**
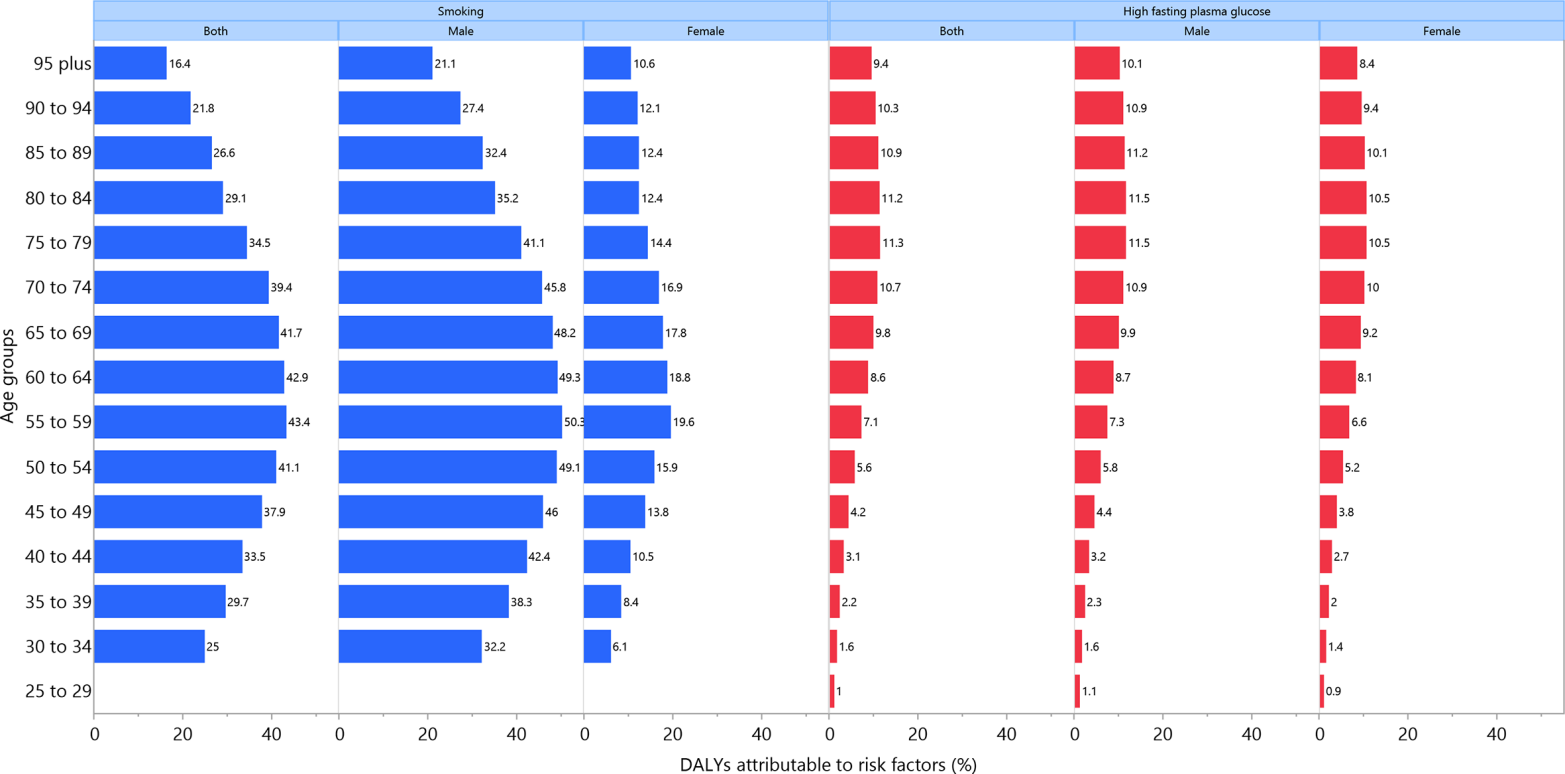
Percentage of DALYs due to bladder cancer attributable to risk factors by age and sex, 2019. DALY, disability-adjusted life year.

Globally in 2019, 36.8% (28.5 to 44.0) of bladder cancer DALYs were attributable to smoking, more so in males (43.7% (34.0 to 51.8) vs. 15.2% (10.9 to 19.4)). In addition, approximately 9.1% (1.9 to 19.6) of the DALYs were attributable to elevated FPG (males: 9.3% (1.6 to 20.9); females: 8.4% (1.6 to 19.2)). For both sexes combined, the percent of bladder cancer DALYs attributable to smoking were highest in east Asia and eastern Europe, whiles those attributable to high FPG were highest in central Latin America and the Caribbean (figure 9). The percent of DALYs attributable to these two risk factors varied between age groups: the highest per cent of bladder cancer DALYs attributable to smoking and high FPG were found in the 55–59 (43.4% (33.6 to 51.5)) and 75–79 (11.3% (2.4 to 24.0)) age groups, respectively, for both sexes (figure 10).

## Discussion

This is the first study to report the incidence, deaths, DALY counts and age-standardised rates for bladder cancer in 204 countries and territories during the period 1990–2019. Age-standardised death, and DALYs decreased significantly globally. While we would have liked to compare our findings with previous non-GBD research, no prior studies have comprehensively reported the country-specific burden of bladder cancer for all countries. In particular, the age-standardised rates found in the current study could not be compared with those reported by the GLOBOCAN, as the two projects have different standard populations and the reporting periods^3^ did not match with the present study. The global incidence and mortality rates of bladder cancer have also been examined elsewhere,^5–7 19^ with the articles having used the GLOBOCAN 2012,^6 7^GBD 2013^5^ and GBD 2016 data,^10^ while country-specific estimates were only provided in the latter two studies. The most up-to-date data on the epidemiology of cancers is GLOBOCAN 2020 study which reports incidence and mortality of 36 cancers, whereas it does not provide DALYs and its country-specific data for bladder cancer have not been used in the recent publication.^9^ The trends in age-standardised incidence and death rates were reported at the global or regional-level, but these were reported across different time intervals, which prevents comparison with the present study.^6 7^ There were two studies which reported changes in the age-standardised rates of developed and developing regions using GBD 2013^5^ and GBD 2016 data.^10^

Despite the differences between our study and previous research, some comparisons are possible. Previous research^7^ reported the highest incidence rates for bladder cancer to be in southern Europe, western Europe and North America, as well as in several countries in northern Africa and western Asia; which is relatively consistent with our findings (ie, that western Europe, central Europe and high-income North America had age-standardised incidence rates >7.5 per 100 000 population). These findings were also supported by another study, which indicated that the highest incidence rates were observed in southern Europe, western Europe and North America.^6^ The same study also reported that the highest death rates were found in western Asia and northern Africa, which concurred with our findings that central Europe, western Europe and north Africa and the Middle East had the highest age-standardised death rates.^6^ In our study, age-standardised incidence rates demonstrated increases from 1990 to 2019 in central Europe, Oceania, southeast Asia, north Africa and the Middle East, east Asia, eastern Europe, and the Caribbean. Previous research has also reported an increase in the age-standardised incidence of bladder cancer in central and eastern Europe, several countries in northern Europe, southern Europe, central and south America, and eastern Asia.^7^

At the country level, Monaco, Lebanon and San Marino had the highest age-standardised incidence rates in 2019, while the highest age-standardised death rates were found in Lebanon, Mali and Monaco. Our study suggests that national-level estimates should be used in national prevention programmes, as global or regional level patterns may be misleading. However, the national-level estimates need to be interpreted with caution in countries where and the quality of the data sources are not high. The variation between the countries and territories could be because of difference in the prevalence of risk factors, detection rate, oncology care and management of health resources between countries, although there could be more reasons for the between-country variations. The risk factors for bladder cancer have been reported in previous studies,^20 21^ but GBD estimated the attributable burden for those that showed a robust association with bladder cancer.^22^ Smoking is one of the most important risk factors, as shown in a meta-analysis of 83 studies, which found the risk of bladder cancer to be considerably higher in current (relative risk: 3.47) and former smokers (relative risk: 2.04), compared with those who had never smoked.^17^ Our study found that smoking accounted for approximately 36.8% (28.5 to 44.0) of bladder cancer DALYs and that removing exposure to smoking at the population-level may reduce the burden of bladder cancer by one-third. Furthermore, this contribution was found to be highest in the 55–59 age group, such that 43.4% (33.6 to 51.5) of bladder cancer DALYs were attributable to this risk factor in that age group. The global prevalence of smoking has decreased by 28.4% in men and 34.4% in women, from 1990 to 2015,^23^ but greater success can be achieved in countries through strategies such as sustainable educational programmes on the health effects of smoking,^24^ increasing taxes on smoking related products and smoking cessation clinics at the primary care level.^25^

Diabetes or high fasting plasma have also been found to be associated with an elevated risk of bladder cancer, with a meta-analysis reporting a 35% higher risk of bladder cancer (relative risk: 1.35).^26^ Our study estimated that 9.1% of the bladder cancer DALYs were attributable to high FPG and that the highest contribution was found in the 75–79 age group (11.3% (2.4 to 24.0)). This contribution may increase in the future, as recent estimates shows that high FPG increased by 37.7% from 1990 to 2017 across the world.^22^ Hence, educational programmes are urgently needed to increase awareness of the risks associated with diabetes and an unhealthy lifestyle, to help reduce the prevalence of high FPG.^27^

Several additional risk factors have also been examined in relation to bladder cancer, but there is no robust evidence for most of them.^20^ The association between alcohol consumption and bladder cancer has been extensively studied, but no clear association has emerged.^28 29^ Vitamins C, D and E, as well as antioxidant supplements, have also been studied in relation to bladder cancer, but meta-analyses have produced conflicting findings.^30 31^ The relationship that bladder cancer has with dietary fluid consumption, including coffee,^32^ tea,^33^ energy drinks^34^ and dairy products^35^ have also produced non-significant or inconsistent findings. Meta-analyses have also investigated the relationship that fruit and vegetable consumption has with bladder cancer risk, which have produced inconsistent results.^36–39^ Meat consumption and the risk of bladder cancer has also been investigated using meta-analysis, but again these have produced inconsistent results.^40 41^ Furthermore, exposure to a number of environmental carcinogens, such as arsenic, nitrates, selenium, cadmium, nuclear power plants, shale gas extraction and the routine use of personal hair dye have been assessed in relation to bladder cancer, but further evidence is needed for most of these risk factors.^20^ A meta-analysis indicated that arsenic in drinking water was associated with a higher risk of bladder cancer. More specifically, the research suggests that exposure to 10 µg/L of arsenic in drinking water may double the risk of bladder cancer, or at the very least, increase it by about 40%.^42^

The association of other factors, such as systemic lupus erythematosus, metabolic syndrome, spinal cord injury, recurrent urinary tract infections, radiotherapy to treat other cancers and physical activity have also been investigated, in relation to the risk of bladder cancer, but the findings were not robust and further research is needed.^20^ The relationship that bladder cancer has with the intake of thiazolidinedione, metformin, sulphonylurea, insulin, analgesics and statins also needs to be clarified.^20^ Based on a review of the available literature, there is currently only sufficient evidence to establish a convincing or probable association between bladder cancer and the risk factors evaluated in this study (smoking and high FPG). So, smoking and high plasma glucose need to be more strongly targeted in prevention programmes, as they are individually responsible for 36.8% and 9.1% of bladder cancer DALYs, respectively.

Finally, the association a country’s socio-demographic level has with bladder cancer’s incidence and death rate has been examined in several previous studies, but these should be interpreted with caution.^5 6^ One of the studies used the human development index (HDI) and assessed its linear association with the incidence and death rate of bladder cancer.^6^ Although HDI is used as a marker of a country’s development level in many studies, one of the components of HDI, life expectancy at birth, is health-related and hence HDI should not be used to compare health outcomes between countries, as it may lead to biased results and an overestimated association. To solve this issue, the GBD project developed the SDI, whose components do not contain health-related variables, and thus health outcomes can be compared more appropriately with socio-demographic level.

Examining the association between health outcomes and development level using linear parameters or categorising countries into different levels of SDI and then comparing the health outcome between these categories may not be valid, and more advanced methods must be used.^5 6^ To address these issues we examined the non-linear association SDI had with the incidence, death and DALY rates of the GBD regions and countries, comparing the observed level of burdens with their corresponding expected levels. These results are therefore more comprehensive than previous work in this area.

Our study has some strengths and limitations. To the best of our knowledge, the current research is the most up-to-date report on the level and trends associated with the burden of bladder cancer for 204 countries and territories from 1990 to 2019. The limitations of the study can be listed as the following: first, all incidence and mortality data may be susceptible to detection biases. GBD attempts to correct for ascertainment bias by adjusting single cause estimates to the all-cause mortality envelope. Some countries struggle with data quality, and in several countries, especially in low-income and middle-income countries, data was missing. To compensate for this problem, as much as possible, MIR-based estimation was used and garbage codes were re-distributed to allow for the inclusion of more data.^1 43^ Garbage codes designate all causes of death that are not useful in the analyses of public health and mortality. Finally, the estimates of bladder cancer in the GBD study were not made using histological data and the burden of bladder cancer attributable to *Schistosoma haematobium* infection could not be calculated.

## Conclusions

This study found considerable inter-country variation in the burden of bladder cancer across the period of study. Although the global age-standardised death, and DALY rates have decreased from 1990 to 2019, there were some countries which registered increases in these rates. Finally, national policy makers should consider the allocation of resources for addressing bladder cancer risk factors, as part of comprehensive prevention programmes based on their national estimates, rather than on global or regional estimates, which may be misleading.

10.1136/bmjgh-2020-004128.supp9 Supplementary data

## Data Availability

Data are available in a public, open access repository. The data used for the analyses are publicly available from the Institute of Health Metrics and Evaluation website (http://ghdx.healthdata.org/gbd-results-tool).
