# Supplementary material for: Global, regional and national burden of bladder cancer and its attributable risk factors in 204 countries and territories, 1990–2019: a systematic analysis for the Global Burden of Disease study 2019
Source: BMJ Glob Health. 2021 Nov 29;6(11):e004128. doi: 10.1136/bmjgh-2020-004128 (PMC8634015; doi:10.1136/bmjgh-2020-004128)
Supplement: Supplementary data [file bmjgh-2020-004128supp001.pdf]

Appendix Table 1: Sequelae for bladder cancer and corresponding disability weights in the GBD 2019 Study

**Sequelae**	**Health state name**	**Lay description**	**DW (95% CI)**
Diagnosis and primary therapy phase	Cancer, diagnosis and primary therapy	Has pain, nausea, fatigue, weight loss and high anxiety.	0.288 (0.193-0.399)
Controlled phase	Generic uncomplicated disease: worry and daily medication	Has a chronic disease that requires medication every day and causes some worry but minimal interference with daily activities.	0.049 (0.031-0.072)
Metastatic phase	Cancer, metastatic	Has severe pain, extreme fatigue, weight loss and high anxiety.	0.451 (0.307-0.600)
Terminal phase	Terminal phase, with medication (for cancers, end-stage kidney/liver disease)	Has lost a lot of weight and regularly uses strong medication to avoid constant pain. The person has no appetite, feels nauseous, and needs to spend most of the day in bed.	0.540 (0.377-0.687)
Urinary incontinence due to bladder cancer	Urinary incontinence	Cannot control urinating.	0.139 (0.094- 0.198)

GBD: Global Burden of Disease

DW: Disability weight
