# Supplementary material for: Global, regional and national burden of bladder cancer and its attributable risk factors in 204 countries and territories, 1990–2019: a systematic analysis for the Global Burden of Disease study 2019
Source: BMJ Glob Health. 2021 Nov 29;6(11):e004128. doi: 10.1136/bmjgh-2020-004128 (PMC8634015; doi:10.1136/bmjgh-2020-004128)
Supplement: Supplementary data [file bmjgh-2020-004128supp002.pdf]

Appendix Table 2: CODEm covariates used, level of covariate, and expected direction of covariate for bladder cancer by sex and age

Cause	Sex	Age Start	Age End	Direction	Level	Covariate Name
Bladder cancer	Male	15-19 years	95+ years	1	2	Alcohol (liters per capita)
Bladder cancer	Female	15-19 years	95+ years	1	2	Alcohol (liters per capita)
Bladder cancer	Male	15-19 years	95+ years	1	1	Cumulative Cigarettes (10 Years)
Bladder cancer	Female	15-19 years	95+ years	1	1	Cumulative Cigarettes (10 Years)
Bladder cancer	Male	15-19 years	95+ years	1	1	Cumulative Cigarettes (15 Years)
Bladder cancer	Female	15-19 years	95+ years	1	1	Cumulative Cigarettes (15 Years)
Bladder cancer	Male	15-19 years	95+ years	1	1	Cumulative Cigarettes (5 Years)
Bladder cancer	Female	15-19 years	95+ years	1	1	Cumulative Cigarettes (5 Years)
Bladder cancer	Male	15-19 years	95+ years	-1	3	Education (years per capita)
Bladder cancer	Female	15-19 years	95+ years	-1	3	Education (years per capita)
Bladder cancer	Male	15-19 years	95+ years	-1	2	Healthcare access and quality index
Bladder cancer	Female	15-19 years	95+ years	-1	2	Healthcare access and quality index
Bladder cancer	Male	15-19 years	95+ years	0	3	LDI (I$ per capita)
Bladder cancer	Female	15-19 years	95+ years	0	3	LDI (I$ per capita)
Bladder cancer	Male	15-19 years	95+ years	1	1	Log-transformed SEV scalar: Bladder C
Bladder cancer	Female	15-19 years	95+ years	1	1	Log-transformed SEV scalar: Bladder C
Bladder cancer	Male	15-19 years	95+ years	1	1	Schistosomiasis Prevalence (proportion)
Bladder cancer	Female	15-19 years	95+ years	1	1	Schistosomiasis Prevalence (proportion)
Bladder cancer	Male	15-19 years	95+ years	1	1	Smoking Prevalence
Bladder cancer	Female	15-19 years	95+ years	1	1	Smoking Prevalence
Bladder cancer	Male	15-19 years	95+ years	0	3	Socio-demographic Index

Bladder cancer	Female	15-19 years	95+ years	0	3	Socio-demographic Index
Bladder cancer	Male	15-19 years	95+ years	-1	2	fruits adjusted(g)
Bladder cancer	Female	15-19 years	95+ years	-1	2	fruits adjusted(g)
Bladder cancer	Male	15-19 years	95+ years	-1	2	vegetables adjusted(g)
Bladder cancer	Female	15-19 years	95+ years	-1	2	vegetables adjusted(g)
