# Supplementary material for: Global, regional and national burden of bladder cancer and its attributable risk factors in 204 countries and territories, 1990–2019: a systematic analysis for the Global Burden of Disease study 2019
Source: BMJ Glob Health. 2021 Nov 29;6(11):e004128. doi: 10.1136/bmjgh-2020-004128 (PMC8634015; doi:10.1136/bmjgh-2020-004128)
Supplement: Supplementary data [file bmjgh-2020-004128supp003.pdf]

Appendix Table 3: Incident cases of bladder cancer and percentage change in age-standardised rates by location, 1990–2019					
	1990		2019		Percentage change in age-standardized rates between 1990 and 2019
	Counts (95% UI)	Rate (95% UI)	Counts (95% UI)	Rate (95% UI)	
Global	234754 (225464 , 243075)	6.3 (6 , 6.5)	524305 (475952 , 569434)	6.5 (5.9 , 7.1)	4 (-4.3 , 13.5)
Canada	3630 (3444 , 3804)	11.2 (10.6 , 11.7)	7257 (5627 , 9199)	10.4 (8 , 13.2)	-7.1 (-29.1 , 17.2)
Greenland	3 (2 , 3)	8.5 (7.6 , 9.6)	5 (4 , 6)	7.1 (5.9 , 8.7)	-15.6 (-31.7 , 3.7)
United States of America	27175 (25994 , 27962)	8.4 (8.1 , 8.7)	49737 (42464 , 58339)	8.8 (7.5 , 10.3)	4.3 (-10.8 , 22)
Australasia	2467 (2349 , 2575)	10.4 (9.9 , 10.9)	4412 (3562 , 5519)	8.7 (7 , 10.9)	-16.9 (-32.5 , 3.2)
Australia	2089 (1987 , 2194)	10.6 (10.1 , 11.1)	3800 (2945 , 4872)	8.8 (6.8 , 11.3)	-16.6 (-35.2 , 5.9)
New Zealand	378 (348 , 407)	9.5 (8.8 , 10.2)	612 (494 , 747)	7.7 (6.2 , 9.4)	-19.1 (-35.4 , -1.3)
High-income Asia Pacific	13879 (13260 , 14375)	7.1 (6.8 , 7.4)	35419 (29617 , 41173)	7.5 (6.4 , 8.7)	5.3 (-8.2 , 20.8)
Brunei Darussalam	6 (5 , 8)	7.9 (6.5 , 10.2)	18 (15 , 21)	7.6 (6.6 , 8.9)	-3.4 (-28.7 , 25.9)
Japan	12358 (11777 , 12824)	7.4 (7 , 7.7)	28596 (23079 , 33974)	7.7 (6.3 , 9.2)	3.8 (-13 , 22.6)
Singapore	126 (116 , 138)	6 (5.5 , 6.6)	364 (279 , 468)	4.8 (3.7 , 6.1)	-20.8 (-39.3 , 2.4)
Republic of Korea	1389 (1300 , 1492)	5.1 (4.8 , 5.6)	6441 (5306 , 7742)	7.3 (6 , 8.7)	42 (18.5 , 71.7)
Western Europe	88072 (85223 , 90305)	15.1 (14.6 , 15.4)	138236 (119202 , 159261)	14.9 (12.8 , 17.3)	-0.9 (-14.5 , 14.5)
Andorra	7 (5 , 10)	13.4 (10.1 , 18.1)	19 (15 , 24)	13.6 (10.3 , 17.5)	1.6 (-30.2 , 47.5)
Austria	1513 (1425 , 1597)	12.6 (11.9 , 13.3)	2270 (1846 , 2805)	12.6 (10.2 , 15.6)	-0.2 (-20.7 , 23.9)
Belgium	2502 (2355 , 2639)	16.1 (15.2 , 17)	3545 (2755 , 4465)	15.3 (11.8 , 19.4)	-5.2 (-27.2 , 20.1)
Cyprus	78 (66 , 90)	10 (8.5 , 11.4)	328 (275 , 387)	16.6 (14 , 19.5)	66.3 (34 , 107.3)
Denmark	1163	14.3	2071	17.7	23.4

	(1098 , 1225)	(13.5 , 15.1)	(1605 , 2652)	(13.6 , 22.9)	(-5.1 , 60.9)
**Finland**	**632 (597 , 666)**	**8.8 (8.3 , 9.3)**	**1016 (795 , 1282)**	**8.1 (6.3 , 10.2)**	**-8.3 (-28.9 , 17)**
**France**	**12209 (11527 , 12872)**	**14.8 (14 , 15.6)**	**21724 (16895 , 27898)**	**16 (12.3 , 20.6)**	**7.9 (-17.2 , 40.1)**
**Germany**	**15666 (14873 , 16404)**	**12.1 (11.5 , 12.7)**	**21670 (16936 , 27585)**	**11 (8.5 , 14.1)**	**-9.1 (-29.8 , 16.1)**
**Greece**	**2988 (2809 , 3169)**	**19.3 (18.2 , 20.5)**	**4594 (3592 , 5775)**	**19.3 (15 , 24.4)**	**-0.4 (-22.7 , 27.2)**
**Iceland**	**39 (35 , 44)**	**13.7 (12.3 , 15.2)**	**69 (59 , 81)**	**12.3 (10.6 , 14.4)**	**-9.7 (-24.8 , 8.7)**
**Ireland**	**402 (376 , 431)**	**9.7 (9 , 10.3)**	**898 (667 , 1168)**	**11.9 (8.8 , 15.4)**	**22.3 (-9.1 , 56.6)**
**Israel**	**445 (410 , 484)**	**9.2 (8.5 , 10)**	**1409 (1098 , 1794)**	**12 (9.4 , 15.3)**	**30.5 (0.7 , 66)**
**Italy**	**19249 (18519 , 20031)**	**21.4 (20.6 , 22.3)**	**28608 (23147 , 34862)**	**19.5 (15.6 , 24.1)**	**-8.6 (-26.1 , 11.8)**
**Luxembourg**	**75 (68 , 82)**	**13.6 (12.5 , 15)**	**137 (111 , 168)**	**13.6 (11 , 16.7)**	**-0.1 (-19 , 22.9)**
**Malta**	**56 (50 , 62)**	**13.2 (11.7 , 14.5)**	**114 (92 , 137)**	**12.2 (9.9 , 14.8)**	**-7.5 (-26.3 , 14.3)**
**Monaco**	**15 (12 , 19)**	**22 (17.2 , 27.4)**	**30 (22 , 54)**	**31.9 (23.3 , 56.9)**	**45.3 (1.2 , 142.4)**
**Netherlands**	**3212 (3057 , 3372)**	**16.1 (15.3 , 16.9)**	**6205 (4844 , 7795)**	**18.1 (14.1 , 22.9)**	**12.6 (-12.4 , 41.5)**
**Norway**	**986 (930 , 1034)**	**14.3 (13.5 , 14.9)**	**1309 (1080 , 1585)**	**13.4 (11 , 16.3)**	**-6.2 (-22.9 , 13.5)**
**Portugal**	**1405 (1332 , 1481)**	**10.1 (9.6 , 10.7)**	**3463 (2708 , 4435)**	**14.7 (11.4 , 18.8)**	**45.2 (10.7 , 86.7)**
**San Marino**	**7 (6 , 9)**	**22.1 (18.9 , 26)**	**17 (13 , 22)**	**25.3 (18.9 , 33.9)**	**14.3 (-16.7 , 56.2)**
**Spain**	**10505 (9880 , 11058)**	**19.1 (18 , 20.1)**	**19459 (15158 , 24672)**	**20.3 (15.6 , 26.2)**	**6 (-18.3 , 36.4)**
**Sweden**	**1606 (1513 , 1696)**	**10.5 (10 , 11.1)**	**2766 (2306 , 3264)**	**12.8 (10.6 , 15.2)**	**21.7 (0.9 , 44.6)**
**Switzerland**	**709 (658 , 756)**	**6.7 (6.2 , 7.2)**	**1776 (1384 , 2275)**	**10 (7.8 , 12.9)**	**49.1 (15.8 , 95.8)**
**United Kingdom**	**12529 (12095 , 12796)**	**13.6 (13.2 , 13.9)**	**14616 (12156 , 17480)**	**11.3 (9.4 , 13.5)**	**-17.4 (-30.9 , -0.8)**
**Southern Latin**	**3281**	**7.2**	**5971**	**7.1**	**-1.1**

**America**	**(3139 , 3416)**	**(6.9 , 7.5)**	**(4739 , 7475)**	**(5.6 , 8.9)**	**(-21.8 , 24.3)**
**Argentina**	**2422 (2294 , 2547)**	**7.6 (7.2 , 7.9)**	**3884 (3064 , 4855)**	**7.1 (5.6 , 8.9)**	**-5.4 (-25.4 , 18.6)**
**Chile**	**451 (421 , 482)**	**4.7 (4.4 , 5)**	**1537 (1177 , 1983)**	**6.4 (4.9 , 8.2)**	**35.5 (2.3 , 75.2)**
**Uruguay**	**408 (381 , 434)**	**10.3 (9.7 , 11)**	**550 (436 , 690)**	**10 (7.9 , 12.7)**	**-2.9 (-25.1 , 25.1)**
**Eastern Europe**	**15492 (14672 , 16532)**	**5.5 (5.2 , 5.8)**	**24384 (21591 , 27362)**	**7.1 (6.3 , 7.9)**	**29 (14.4 , 44.8)**
**Belarus**	**728 (669 , 794)**	**5.6 (5.1 , 6.1)**	**987 (763 , 1277)**	**6.2 (4.8 , 8)**	**11.1 (-14.4 , 45.6)**
**Estonia**	**149 (138 , 161)**	**7.3 (6.8 , 7.8)**	**278 (216 , 350)**	**10.4 (8.1 , 13.3)**	**43.9 (10.2 , 88.4)**
**Latvia**	**240 (222 , 260)**	**6.7 (6.2 , 7.3)**	**421 (334 , 524)**	**10.5 (8.3 , 13.1)**	**56.5 (22.8 , 97)**
**Lithuania**	**296 (278 , 317)**	**6.6 (6.2 , 7.1)**	**417 (329 , 516)**	**7.1 (5.6 , 8.9)**	**8.2 (-15.8 , 35.8)**
**Republic of Moldova**	**227 (208 , 249)**	**5.1 (4.7 , 5.6)**	**350 (295 , 420)**	**6 (5.1 , 7.2)**	**18.1 (-1.2 , 39)**
**Russian Federation**	**10354 (10034 , 10740)**	**5.7 (5.5 , 5.9)**	**16548 (14031 , 19249)**	**7 (6 , 8.2)**	**24.3 (5.9 , 44.3)**
**Ukraine**	**3498 (2959 , 4256)**	**4.8 (4.1 , 5.8)**	**5384 (4444 , 6436)**	**7.1 (5.9 , 8.5)**	**48.1 (9.2 , 92.4)**
**Central Europe**	**12417 (12063 , 12735)**	**8.4 (8.1 , 8.6)**	**27057 (23605 , 30687)**	**12.6 (11 , 14.3)**	**50.3 (30.3 , 70.7)**
**Albania**	**37 (34 , 40)**	**1.8 (1.7 , 2)**	**100 (75 , 132)**	**2.4 (1.8 , 3.1)**	**30.3 (-4.2 , 71.1)**
**Bosnia and Herzegovina**	**206 (190 , 222)**	**5.2 (4.8 , 5.6)**	**605 (473 , 759)**	**10 (7.8 , 12.5)**	**91.3 (48.1 , 139.3)**
**Bulgaria**	**876 (813 , 939)**	**7 (6.5 , 7.5)**	**1723 (1335 , 2163)**	**12.2 (9.4 , 15.3)**	**74.9 (35.5 , 121.8)**
**Croatia**	**698 (645 , 756)**	**11 (10.2 , 11.9)**	**1328 (1038 , 1673)**	**15.1 (11.7 , 19)**	**36.6 (6 , 75.5)**
**Czechia**	**1741 (1644 , 1835)**	**12.6 (11.9 , 13.2)**	**3116 (2462 , 3802)**	**14.7 (11.7 , 18)**	**17 (-7.7 , 44.3)**
**Hungary**	**1483 (1408 , 1559)**	**10.1 (9.6 , 10.6)**	**2564 (2080 , 3106)**	**13.5 (11 , 16.4)**	**34.1 (7.8 , 63.9)**
**Montenegro**	**53 (45 , 61)**	**8.6 (7.2 , 9.9)**	**108 (89 , 132)**	**10.9 (9 , 13.2)**	**27.5 (-2.3 , 69.1)**

**North Macedonia**	**143 (126 , 160)**	**7.7 (6.8 , 8.6)**	**410 (322 , 523)**	**12.7 (10 , 16)**	**64.2 (27.7 , 112.6)**
**Poland**	**3555 (3428 , 3674)**	**8.1 (7.8 , 8.4)**	**9055 (7438 , 10938)**	**12.9 (10.5 , 15.6)**	**59.2 (29.6 , 93.5)**
**Romania**	**1881 (1784 , 1979)**	**6.6 (6.3 , 6.9)**	**4194 (3383 , 5207)**	**11.5 (9.4 , 14.4)**	**74.8 (41.9 , 118.4)**
**Serbia**	**983 (817 , 1143)**	**8.7 (7.2 , 10.1)**	**2302 (1792 , 2901)**	**14.3 (11.2 , 18)**	**64.8 (24.3 , 119.3)**
**Slovakia**	**574 (537 , 614)**	**9.5 (8.9 , 10.2)**	**1152 (909 , 1453)**	**12.3 (9.7 , 15.6)**	**29.3 (1.3 , 64.8)**
**Slovenia**	**188 (144 , 239)**	**7.7 (5.9 , 9.8)**	**400 (305 , 513)**	**9.1 (6.9 , 11.8)**	**18.4 (-18.1 , 67.4)**
**Central Asia**	**1643 (1439 , 1878)**	**3.5 (3.1 , 4)**	**3258 (2931 , 3600)**	**4.6 (4.1 , 5)**	**30.5 (13.8 , 56.5)**
**Armenia**	**215 (183 , 243)**	**8.2 (7 , 9.3)**	**417 (345 , 500)**	**10 (8.3 , 12)**	**22.3 (-5.4 , 55.3)**
**Azerbaijan**	**179 (153 , 207)**	**3.4 (2.9 , 3.9)**	**447 (362 , 551)**	**4.7 (3.9 , 5.7)**	**37.3 (6.8 , 74.3)**
**Georgia**	**353 (291 , 412)**	**5.6 (4.7 , 6.5)**	**503 (419 , 594)**	**8.6 (7.1 , 10.2)**	**52.7 (22 , 91.5)**
**Kazakhstan**	**487 (395 , 630)**	**3.8 (3.1 , 4.9)**	**792 (670 , 937)**	**4.6 (3.9 , 5.4)**	**19.8 (-7.5 , 55.5)**
**Kyrgyzstan**	**78 (69 , 88)**	**2.6 (2.3 , 2.9)**	**115 (97 , 136)**	**2.5 (2.1 , 3)**	**-1.4 (-19.4 , 20.5)**
**Mongolia**	**31 (25 , 37)**	**2.9 (2.4 , 3.4)**	**44 (34 , 56)**	**2 (1.6 , 2.5)**	**-31.1 (-48.6 , -8.7)**
**Tajikistan**	**48 (35 , 59)**	**1.7 (1.2 , 2.2)**	**101 (82 , 125)**	**2.3 (1.9 , 2.8)**	**35.3 (-0.7 , 95.8)**
**Turkmenistan**	**25 (21 , 28)**	**1.3 (1.2 , 1.5)**	**102 (80 , 132)**	**2.5 (2 , 3.2)**	**88.8 (45 , 143)**
**Uzbekistan**	**228 (170 , 319)**	**2 (1.4 , 2.8)**	**735 (602 , 886)**	**3.6 (3 , 4.2)**	**81 (25.5 , 159.8)**
**Central Latin America**	**1826 (1746 , 1898)**	**2.3 (2.2 , 2.4)**	**6232 (5333 , 7280)**	**2.7 (2.3 , 3.1)**	**16.3 (0.2 , 35)**
**Colombia**	**479 (448 , 512)**	**2.9 (2.7 , 3.1)**	**1355 (1041 , 1755)**	**2.6 (2 , 3.3)**	**-11.3 (-32.5 , 14.2)**
**Costa Rica**	**62 (57 , 68)**	**3.7 (3.4 , 4)**	**210 (160 , 267)**	**4.1 (3.2 , 5.3)**	**12.4 (-14.1 , 45.5)**
**El Salvador**	**35 (32 , 38)**	**1.2 (1.1 , 1.3)**	**111 (85 , 142)**	**1.8 (1.4 , 2.4)**	**50.5 (12.3 , 94.7)**

**Guatemala**	**43**<br>**(37 , 49)**	**1.4**<br>**(1.2 , 1.6)**	**166**<br>**(129 , 210)**	**1.5**<br>**(1.2 , 1.9)**	**12**<br>**(-14.5 , 45.8)**
**Honduras**	**25**<br>**(20 , 30)**	**1.3**<br>**(1 , 1.5)**	**123**<br>**(93 , 177)**	**2.2**<br>**(1.7 , 3.2)**	**69**<br>**(24.4 , 143.3)**
**Mexico**	**889**<br>**(849 , 924)**	**2.2**<br>**(2.1 , 2.3)**	**3071**<br>**(2633 , 3597)**	**2.7**<br>**(2.3 , 3.1)**	**22.1**<br>**(4.4 , 43.7)**
**Nicaragua**	**20**<br>**(17 , 22)**	**1.3**<br>**(1.1 , 1.5)**	**82**<br>**(67 , 99)**	**2**<br>**(1.6 , 2.4)**	**45.7**<br>**(16.1 , 88.8)**
**Panama**	**34**<br>**(31 , 37)**	**2.3**<br>**(2.1 , 2.6)**	**99**<br>**(75 , 127)**	**2.4**<br>**(1.8 , 3.1)**	**2.3**<br>**(-23.7 , 33.7)**
**Venezuela (Bolivarian Republic of)**	**240**<br>**(224 , 257)**	**2.6**<br>**(2.4 , 2.8)**	**1016**<br>**(764 , 1308)**	**3.6**<br>**(2.7 , 4.6)**	**37.6**<br>**(2.8 , 79.2)**
**Andean Latin America**	**402**<br>**(362 , 447)**	**2.1**<br>**(1.9 , 2.3)**	**1384**<br>**(1125 , 1679)**	**2.5**<br>**(2.1 , 3.1)**	**21**<br>**(-3.4 , 50.8)**
**Bolivia (Plurinational State of)**	**79**<br>**(63 , 97)**	**2.7**<br>**(2.2 , 3.3)**	**285**<br>**(222 , 359)**	**3.5**<br>**(2.7 , 4.3)**	**28.3**<br>**(-2.4 , 69.5)**
**Ecuador**	**95**<br>**(87 , 102)**	**1.9**<br>**(1.7 , 2)**	**377**<br>**(296 , 475)**	**2.6**<br>**(2 , 3.3)**	**37.5**<br>**(6.7 , 73.4)**
**Peru**	**228**<br>**(196 , 267)**	**2**<br>**(1.7 , 2.3)**	**722**<br>**(542 , 956)**	**2.3**<br>**(1.7 , 3)**	**12.2**<br>**(-17.8 , 54.1)**
**Caribbean**	**1148**<br>**(1083 , 1208)**	**4.5**<br>**(4.3 , 4.8)**	**2821**<br>**(2424 , 3303)**	**5.5**<br>**(4.7 , 6.4)**	**20.7**<br>**(2.5 , 41)**
**Antigua and Barbuda**	**2**<br>**(2 , 2)**	**3.8**<br>**(3.4 , 4.1)**	**5**<br>**(4 , 6)**	**4.9**<br>**(4.1 , 5.8)**	**30.2**<br>**(6.9 , 55.9)**
**Barbados**	**11**<br>**(10 , 12)**	**3.7**<br>**(3.3 , 4)**	**24**<br>**(20 , 29)**	**4.8**<br>**(4 , 5.8)**	**32.4**<br>**(6 , 60.7)**
**Belize**	**2**<br>**(2 , 2)**	**2.2**<br>**(2 , 2.5)**	**9**<br>**(7 , 10)**	**3.3**<br>**(2.8 , 3.8)**	**46**<br>**(20.7 , 74.1)**
**Bermuda**	**7**<br>**(6 , 8)**	**11.6**<br>**(10.5 , 12.8)**	**15**<br>**(12 , 18)**	**11.5**<br>**(9.5 , 13.9)**	**-0.9**<br>**(-19.3 , 22.6)**
**Bahamas**	**4**<br>**(4 , 5)**	**2.8**<br>**(2.6 , 3.2)**	**12**<br>**(9 , 14)**	**3.1**<br>**(2.5 , 3.7)**	**7.8**<br>**(-13.7 , 33.8)**
**Cuba**	**654**<br>**(615 , 691)**	**6.4**<br>**(6 , 6.7)**	**1627**<br>**(1314 , 2030)**	**8.5**<br>**(6.8 , 10.7)**	**33.7**<br>**(7.1 , 66.9)**
**Dominica**	**4**<br>**(3 , 4)**	**4.8**<br>**(4.2 , 5.4)**	**5**<br>**(4 , 6)**	**5.6**<br>**(4.6 , 6.9)**	**16.1**<br>**(-6.1 , 45)**
**Dominican**	**47**	**1.4**	**198**	**2.2**	**61.4**

**Republic**	**(40 , 54)**	**(1.2 , 1.6)**	**(145 , 268)**	**(1.6 , 2.9)**	**(14.6 , 126.2)**
**Grenada**	**3<br>(2 , 3)**	**3.7<br>(3.3 , 4.2)**	**5<br>(5 , 6)**	**5<br>(4.4 , 5.7)**	**35.5<br>(12.1 , 60.6)**
**Guyana**	**9<br>(7 , 10)**	**2.4<br>(2.1 , 2.7)**	**16<br>(12 , 21)**	**2.7<br>(2.1 , 3.4)**	**12.2<br>(-15.8 , 47.9)**
**Haiti**	**100<br>(68 , 132)**	**3.4<br>(2.3 , 4.5)**	**207<br>(128 , 305)**	**3.3<br>(2.1 , 4.8)**	**-2.1<br>(-26.7 , 29.4)**
**Jamaica**	**70<br>(63 , 77)**	**3.9<br>(3.5 , 4.3)**	**120<br>(94 , 151)**	**4<br>(3.1 , 5.1)**	**3.2<br>(-20.6 , 31)**
**Puerto Rico**	**156<br>(143 , 168)**	**4.3<br>(4 , 4.7)**	**384<br>(298 , 493)**	**5.3<br>(4.1 , 6.9)**	**22.6<br>(-6.9 , 59.3)**
**Saint Kitts and Nevis**	**2<br>(2 , 3)**	**6.1<br>(5.5 , 6.7)**	**4<br>(3 , 5)**	**6.6<br>(5.5 , 7.7)**	**7<br>(-12.4 , 30.7)**
**Saint Lucia**	**4<br>(4 , 5)**	**5.2<br>(4.8 , 5.7)**	**12<br>(10 , 14)**	**5.6<br>(4.7 , 6.6)**	**6.9<br>(-12.2 , 28.3)**
**Saint Vincent and the Grenadines**	**2<br>(2 , 3)**	**3.4<br>(3.1 , 3.8)**	**6<br>(5 , 6)**	**4.2<br>(3.6 , 4.9)**	**22.2<br>(4 , 43.2)**
**Suriname**	**5<br>(5 , 6)**	**2.2<br>(1.9 , 2.4)**	**16<br>(13 , 19)**	**2.8<br>(2.3 , 3.3)**	**28.4<br>(4.6 , 56.5)**
**Trinidad and Tobago**	**25<br>(23 , 27)**	**3<br>(2.8 , 3.3)**	**54<br>(40 , 71)**	**2.9<br>(2.2 , 3.9)**	**-3.3<br>(-30.2 , 28.6)**
**United States Virgin Islands**	**2<br>(2 , 2)**	**2.6<br>(2.1 , 3.1)**	**7<br>(6 , 9)**	**3.9<br>(3.3 , 4.7)**	**53.6<br>(17.3 , 107.4)**
**Tropical Latin America**	**3241<br>(3107 , 3355)**	**3.8<br>(3.6 , 4)**	**9713<br>(8975 , 10358)**	**4.1<br>(3.8 , 4.4)**	**7.3<br>(-0.3 , 14.6)**
**Brazil**	**3208<br>(3075 , 3320)**	**3.9<br>(3.7 , 4)**	**9589<br>(8850 , 10230)**	**4.1<br>(3.8 , 4.4)**	**6.6<br>(-1 , 13.8)**
**Paraguay**	**34<br>(29 , 39)**	**1.6<br>(1.3 , 1.8)**	**124<br>(94 , 162)**	**2.3<br>(1.8 , 3)**	**46.6<br>(5.2 , 103.3)**
**East Asia**	**27180<br>(23878 , 30628)**	**3.4<br>(3 , 3.8)**	**105424<br>(88439 , 124569)**	**5.3<br>(4.4 , 6.2)**	**55.6<br>(26.1 , 95.8)**
**China**	**25549<br>(22268 , 29008)**	**3.3<br>(2.9 , 3.7)**	**100020<br>(83242 , 118654)**	**5.2<br>(4.3 , 6.1)**	**56.2<br>(25.7 , 98.3)**
**Democratic People's Republic of Korea**	**507<br>(374 , 666)**	**3.2<br>(2.4 , 4.1)**	**1022<br>(840 , 1237)**	**3.2<br>(2.6 , 3.9)**	**-0.1<br>(-25.9 , 38.7)**
**Taiwan (Province of China)**	**1125<br>(1071 , 1180)**	**7.2<br>(6.9 , 7.6)**	**4382<br>(3373 , 5782)**	**11.2<br>(8.6 , 14.7)**	**54.5<br>(18.5 , 101.7)**

**Southeast Asia**	**5350 (4774 , 5911)**	**2.3 (2 , 2.5)**	**16340 (14192 , 19136)**	**2.8 (2.5 , 3.3)**	**26.1 (7.1 , 50.5)**
**Cambodia**	**90 (68 , 116)**	**2.2 (1.6 , 2.8)**	**304 (230 , 378)**	**2.7 (2.1 , 3.4)**	**24.7 (-7.1 , 60.9)**
**Indonesia**	**1641 (1357 , 1979)**	**1.8 (1.5 , 2.2)**	**5021 (3738 , 7241)**	**2.6 (1.9 , 3.6)**	**39.3 (6.7 , 80.8)**
**Lao People's Democratic Republic**	**46 (32 , 61)**	**2.4 (1.6 , 3.1)**	**95 (69 , 121)**	**2.4 (1.7 , 2.9)**	**-0.1 (-24.4 , 34.6)**
**Malaysia**	**330 (270 , 382)**	**3.9 (3.2 , 4.6)**	**1390 (1083 , 1748)**	**5.6 (4.4 , 6.9)**	**42.4 (3 , 103.7)**
**Maldives**	**2 (2 , 3)**	**3.2 (2.6 , 4.1)**	**11 (9 , 13)**	**3.8 (3.1 , 4.6)**	**19.4 (-15 , 64.6)**
**Mauritius**	**38 (35 , 41)**	**5.2 (4.8 , 5.6)**	**72 (58 , 91)**	**4.2 (3.4 , 5.2)**	**-20.1 (-37 , 1.2)**
**Myanmar**	**513 (357 , 692)**	**2.4 (1.7 , 3.1)**	**1053 (848 , 1312)**	**2.4 (2 , 3)**	**1.1 (-24.1 , 40.1)**
**Philippines**	**522 (452 , 598)**	**1.8 (1.6 , 2)**	**1356 (1083 , 1683)**	**1.8 (1.4 , 2.2)**	**-1.9 (-23.8 , 27.6)**
**Sri Lanka**	**152 (134 , 172)**	**1.5 (1.4 , 1.8)**	**679 (500 , 902)**	**2.7 (2 , 3.6)**	**76.6 (29.6 , 141)**
**Seychelles**	**4 (3 , 4)**	**6.6 (5.7 , 7.7)**	**9 (7 , 10)**	**8.3 (7.1 , 9.6)**	**25.8 (4.3 , 48.7)**
**Thailand**	**1286 (1115 , 1467)**	**3.9 (3.4 , 4.4)**	**3273 (2439 , 4263)**	**3.3 (2.4 , 4.3)**	**-15.3 (-38.9 , 14.2)**
**Timor-Leste**	**4 (3 , 6)**	**1.6 (1.1 , 2.3)**	**16 (11 , 21)**	**2.1 (1.5 , 2.8)**	**27.9 (-7.6 , 89.6)**
**Viet Nam**	**716 (574 , 861)**	**1.8 (1.5 , 2.2)**	**3040 (2367 , 3782)**	**3.4 (2.6 , 4.1)**	**81.7 (38.1 , 146.2)**
**Oceania**	**53 (42 , 66)**	**1.9 (1.5 , 2.3)**	**170 (134 , 218)**	**2.5 (2 , 3.1)**	**32 (8.8 , 61.3)**
**American Samoa**	**1 (0 , 1)**	**2.6 (2.3 , 2.9)**	**2 (2 , 2)**	**3.9 (3.3 , 4.5)**	**50.2 (20.4 , 84.5)**
**Cook Islands**	**1 (1 , 1)**	**5.7 (4.7 , 6.7)**	**2 (1 , 2)**	**6.7 (5.4 , 8.1)**	**18.1 (-11.9 , 51.9)**
**Micronesia (Federated States of)**	**1 (1 , 2)**	**2.8 (2.2 , 3.5)**	**3 (2 , 4)**	**3.9 (2.9 , 5.1)**	**41.1 (-0.6 , 94.4)**
**Fiji**	**8 (6 , 10)**	**2.3 (1.9 , 2.8)**	**23 (18 , 29)**	**3.1 (2.5 , 3.9)**	**37.9 (0.9 , 89.3)**

**Guam**	**2**<br>**(2 , 3)**	**3.1**<br>**(2.7 , 3.6)**	**7**<br>**(6 , 8)**	**3.6**<br>**(3 , 4.4)**	**17.5**<br>**(-7.1 , 48.8)**
**Kiribati**	**1**<br>**(1 , 1)**	**1.9**<br>**(1.6 , 2.3)**	**1**<br>**(1 , 2)**	**2**<br>**(1.6 , 2.4)**	**4.8**<br>**(-18.6 , 31.8)**
**Marshall Islands**	**0**<br>**(0 , 1)**	**2.9**<br>**(2.2 , 3.6)**	**1**<br>**(1 , 2)**	**3.6**<br>**(2.8 , 4.6)**	**26.3**<br>**(-3 , 66.6)**
**Nauru**	**0**<br>**(0 , 0)**	**3.8**<br>**(2.9 , 4.9)**	**0**<br>**(0 , 0)**	**4.9**<br>**(3.7 , 6.3)**	**27.1**<br>**(0.9 , 62.8)**
**Niue**	**0**<br>**(0 , 0)**	**3.1**<br>**(2.5 , 3.8)**	**0**<br>**(0 , 0)**	**4.4**<br>**(3.4 , 5.5)**	**44.3**<br>**(9.5 , 83.9)**
**Northern Mariana Islands**	**0**<br>**(0 , 1)**	**2.6**<br>**(2.2 , 3.1)**	**3**<br>**(2 , 3)**	**5.6**<br>**(4.7 , 6.7)**	**119.8**<br>**(71.9 , 169.9)**
**Palau**	**0**<br>**(0 , 0)**	**1.7**<br>**(1.4 , 2.1)**	**0**<br>**(0 , 1)**	**2.2**<br>**(1.7 , 2.7)**	**27.1**<br>**(-6.7 , 68.5)**
**Papua New Guinea**	**27**<br>**(19 , 36)**	**1.5**<br>**(1.1 , 2)**	**96**<br>**(68 , 134)**	**2.1**<br>**(1.5 , 2.8)**	**37.2**<br>**(4.8 , 76.6)**
**Samoa**	**2**<br>**(2 , 3)**	**3**<br>**(2.4 , 3.6)**	**5**<br>**(4 , 6)**	**3.4**<br>**(2.7 , 4.3)**	**15.3**<br>**(-10.6 , 52.5)**
**Solomon Islands**	**4**<br>**(2 , 5)**	**2.7**<br>**(1.9 , 3.8)**	**12**<br>**(8 , 17)**	**3.8**<br>**(2.8 , 5.1)**	**40**<br>**(5.2 , 87.7)**
**Tokelau**	**0**<br>**(0 , 0)**	**2.4**<br>**(1.9 , 3.1)**	**0**<br>**(0 , 0)**	**3.3**<br>**(2.5 , 4.4)**	**39.6**<br>**(3.6 , 86.8)**
**Tonga**	**1**<br>**(1 , 1)**	**2**<br>**(1.5 , 2.5)**	**2**<br>**(2 , 3)**	**2.8**<br>**(2 , 3.7)**	**40.5**<br>**(8.3 , 83.7)**
**Tuvalu**	**0**<br>**(0 , 0)**	**2.6**<br>**(2.1 , 3.3)**	**0**<br>**(0 , 0)**	**3.3**<br>**(2.6 , 4.3)**	**27.5**<br>**(-4 , 70.3)**
**Vanuatu**	**1**<br>**(1 , 2)**	**1.9**<br>**(1.4 , 2.6)**	**5**<br>**(3 , 6)**	**2.7**<br>**(2 , 3.6)**	**44.9**<br>**(6.4 , 98.2)**
**North Africa and Middle East**	**10647**<br>**(9192 , 12043)**	**6.3**<br>**(5.3 , 7.1)**	**41289**<br>**(34669 , 49951)**	**9.6**<br>**(8.1 , 11.4)**	**52.5**<br>**(21.3 , 107.1)**
**Afghanistan**	**310**<br>**(192 , 456)**	**4.7**<br>**(2.9 , 6.7)**	**511**<br>**(354 , 696)**	**4.6**<br>**(3.2 , 6)**	**-2.5**<br>**(-29.2 , 33.2)**
**Algeria**	**413**<br>**(322 , 524)**	**4.1**<br>**(3.3 , 5.1)**	**1391**<br>**(1097 , 1736)**	**4.5**<br>**(3.6 , 5.7)**	**11.5**<br>**(-20.8 , 52.2)**
**Bahrain**	**17**<br>**(14 , 20)**	**12.3**<br>**(10.2 , 14.6)**	**82**<br>**(60 , 107)**	**11.3**<br>**(8.7 , 14.2)**	**-7.8**<br>**(-33 , 25.8)**
**Egypt**	**3864**<br>**(3531 , 4278)**	**11.8**<br>**(10.7 , 12.9)**	**13736**<br>**(8925 , 20261)**	**19.1**<br>**(12.5 , 28)**	**62.7**<br>**(4.6 , 142.6)**
**Iran (Islamic**	**997**	**4.1**	**4585**	**6.4**	**56.8**

**Republic of)**	**(791 , 1173)**	**(3.2 , 4.9)**	**(4189 , 4998)**	**(5.8 , 7)**	**(28.4 , 108.4)**
**Iraq**	**581 (418 , 757)**	**7.8 (5.6 , 10.2)**	**2940 (2249 , 3646)**	**13.8 (10.8 , 16.7)**	**76.6 (22.5 , 167.1)**
**Jordan**	**72 (59 , 88)**	**6.1 (4.9 , 7.5)**	**532 (419 , 673)**	**8.7 (6.9 , 11)**	**43.5 (4.7 , 103.8)**
**Kuwait**	**48 (42 , 54)**	**8.5 (7.3 , 9.6)**	**245 (195 , 307)**	**10.7 (8.6 , 13.3)**	**26.9 (-1.2 , 61.9)**
**Lebanon**	**407 (327 , 504)**	**19.3 (15.6 , 23.6)**	**1574 (1194 , 2111)**	**30.2 (23 , 40.4)**	**56 (13.5 , 133)**
**Libya**	**149 (105 , 196)**	**8.5 (6 , 11.4)**	**546 (401 , 711)**	**11.4 (8.4 , 14.8)**	**33.4 (-13.1 , 123.5)**
**Morocco**	**330 (251 , 406)**	**2.6 (1.9 , 3.2)**	**1257 (931 , 1620)**	**4.3 (3.2 , 5.5)**	**64 (17.6 , 126.6)**
**Palestine**	**51 (35 , 68)**	**6.2 (4.3 , 8.3)**	**165 (138 , 198)**	**7.5 (6.3 , 9)**	**20.4 (-14.9 , 90.5)**
**Oman**	**23 (15 , 30)**	**3.7 (2.5 , 5)**	**96 (75 , 125)**	**6.3 (5.2 , 7.6)**	**67.1 (13.6 , 167.3)**
**Qatar**	**4 (3 , 5)**	**5.4 (3.9 , 7)**	**62 (43 , 88)**	**12.2 (9.1 , 16.2)**	**126.5 (51.5 , 243.7)**
**Saudi Arabia**	**144 (97 , 194)**	**2.7 (1.8 , 3.7)**	**818 (618 , 1083)**	**4.6 (3.6 , 5.8)**	**66.8 (12.4 , 190.6)**
**Sudan**	**381 (186 , 910)**	**4.4 (2.1 , 10.7)**	**864 (567 , 1538)**	**5 (3.3 , 8.9)**	**13 (-29.7 , 110.9)**
**Syrian Arab Republic**	**145 (109 , 184)**	**2.9 (2.1 , 3.7)**	**539 (397 , 729)**	**4.6 (3.4 , 6.1)**	**59.2 (6.2 , 154.2)**
**Tunisia**	**333 (261 , 416)**	**7.2 (5.7 , 8.9)**	**1309 (927 , 1833)**	**10.7 (7.6 , 14.8)**	**48.4 (-1.8 , 130.9)**
**Turkey**	**2159 (1699 , 2663)**	**6.3 (4.9 , 7.7)**	**8884 (7048 , 10927)**	**10.1 (8.1 , 12.4)**	**61.9 (19.6 , 132)**
**United Arab Emirates**	**49 (24 , 115)**	**11.6 (5.4 , 29.5)**	**576 (312 , 983)**	**12.3 (6.5 , 21.2)**	**5.6 (-42.3 , 96.5)**
**Yemen**	**164 (86 , 262)**	**3.6 (1.9 , 5.8)**	**536 (382 , 725)**	**4.4 (3.1 , 5.8)**	**20.5 (-19.4 , 102.9)**
**South Asia**	**10260 (8782 , 11707)**	**2.2 (1.8 , 2.5)**	**31553 (27900 , 35688)**	**2.4 (2.1 , 2.7)**	**9.4 (-7.7 , 33.2)**
**Bangladesh**	**631 (487 , 780)**	**1.5 (1.1 , 1.8)**	**1964 (1377 , 2635)**	**1.6 (1.1 , 2.1)**	**8.5 (-22.9 , 42.5)**
**Bhutan**	**3 (2 , 4)**	**1.3 (0.8 , 1.8)**	**11 (7 , 14)**	**2.1 (1.4 , 2.7)**	**58.8 (15.6 , 118.9)**

India	6256<br>(5431 , 7212)	1.6<br>(1.4 , 1.9)	21058<br>(18039 , 24411)	2<br>(1.7 , 2.3)	19.6<br>(-1.2 , 45.1)
Nepal	108<br>(70 , 148)	1.3<br>(0.8 , 1.8)	367<br>(261 , 473)	1.8<br>(1.3 , 2.3)	40<br>(4.2 , 88.1)
Pakistan	3263<br>(2637 , 3905)	6.1<br>(4.9 , 7.3)	8153<br>(6395 , 10336)	8<br>(6.3 , 10.2)	32.2<br>(-1.7 , 81.9)
Southern Sub-Saharan Africa	1082<br>(965 , 1196)	4<br>(3.5 , 4.4)	2200<br>(1959 , 2449)	4<br>(3.6 , 4.5)	1.5<br>(-13.4 , 20)
Botswana	19<br>(14 , 24)	3.5<br>(2.7 , 4.4)	60<br>(43 , 79)	4.4<br>(3.3 , 5.7)	27.6<br>(-9 , 76.4)
Lesotho	20<br>(16 , 27)	2.2<br>(1.7 , 2.8)	37<br>(25 , 49)	3.1<br>(2.2 , 4)	40.7<br>(3.9 , 93.3)
Namibia	14<br>(11 , 17)	2<br>(1.6 , 2.5)	36<br>(28 , 46)	2.7<br>(2.1 , 3.4)	33<br>(0 , 75.1)
South Africa	637<br>(556 , 715)	3<br>(2.6 , 3.4)	1365<br>(1232 , 1493)	3.2<br>(2.9 , 3.5)	4.6<br>(-7.3 , 20.8)
Eswatini	8<br>(7 , 11)	3<br>(2.4 , 4)	19<br>(14 , 25)	3.4<br>(2.6 , 4.4)	13.6<br>(-16.2 , 54.4)
Zimbabwe	384<br>(334 , 439)	10.1<br>(8.8 , 11.5)	684<br>(447 , 888)	10.5<br>(6.9 , 13.5)	4.4<br>(-32.3 , 40.4)
Western Sub-Saharan Africa	2462<br>(2060 , 2935)	3<br>(2.6 , 3.6)	5067<br>(3497 , 6105)	2.9<br>(2.1 , 3.5)	-3.7<br>(-39.3 , 24.9)
Benin	80<br>(63 , 118)	4.2<br>(3.4 , 6.2)	132<br>(100 , 169)	2.9<br>(2.2 , 3.6)	-31.9<br>(-61.5 , -5.3)
Burkina Faso	162<br>(112 , 271)	4.1<br>(2.9 , 6.9)	232<br>(152 , 317)	2.7<br>(1.8 , 3.7)	-34.1<br>(-71.1 , -3.6)
Cameroon	180<br>(146 , 223)	4.5<br>(3.7 , 5.5)	446<br>(266 , 612)	3.9<br>(2.4 , 5.3)	-12.5<br>(-42.3 , 18.4)
Cabo Verde	3<br>(3 , 4)	1.3<br>(1.2 , 1.5)	22<br>(18 , 26)	5.1<br>(4.3 , 6)	284.2<br>(214.1 , 362.8)
Chad	93<br>(70 , 118)	3.5<br>(2.6 , 4.4)	167<br>(120 , 219)	3.2<br>(2.3 , 4.2)	-7.2<br>(-27.6 , 18.1)
CÃ´te d'Ivoire	173<br>(130 , 232)	5<br>(3.8 , 6.6)	341<br>(256 , 443)	3.5<br>(2.7 , 4.4)	-30.3<br>(-55.6 , -4.5)
Gambia	6<br>(5 , 8)	1.9<br>(1.5 , 2.4)	21<br>(16 , 29)	2.4<br>(1.8 , 3.2)	26.9<br>(-12.5 , 80.1)
Ghana	255<br>(200 , 393)	4.2<br>(3.3 , 6.6)	634<br>(336 , 841)	3.8<br>(2.1 , 4.9)	-9.7<br>(-51 , 29.9)
Guinea	186	6	355	7	15.3

	(153 , 221)	(5 , 7.1)	(238 , 493)	(4.7 , 9.6)	(-23.1 , 71.9)
**Guinea-Bissau**	18<br>(13 , 26)	4.8<br>(3.6 , 6.6)	25<br>(19 , 33)	3.6<br>(2.8 , 4.7)	-25.2<br>(-45.9 , 1.5)
**Liberia**	51<br>(35 , 100)	4.9<br>(3.4 , 9.6)	60<br>(43 , 90)	3.2<br>(2.3 , 4.7)	-36.1<br>(-60.5 , -3.3)
**Mali**	407<br>(338 , 474)	10.8<br>(9.1 , 12.5)	909<br>(400 , 1254)	11.6<br>(5 , 15.8)	8.2<br>(-53.4 , 53.6)
**Mauritania**	44<br>(35 , 55)	4.6<br>(3.6 , 5.8)	61<br>(43 , 88)	3.1<br>(2.2 , 4.4)	-31.7<br>(-51.6 , -5)
**Niger**	88<br>(62 , 114)	3.6<br>(2.5 , 4.5)	178<br>(110 , 253)	2.6<br>(1.6 , 3.6)	-27.8<br>(-53.4 , -2.2)
**Nigeria**	445<br>(324 , 578)	1.1<br>(0.8 , 1.5)	1033<br>(813 , 1312)	1.4<br>(1.1 , 1.7)	22.8<br>(-15.5 , 80.7)
**Sao Tome and Principe**	3<br>(2 , 3)	4.4<br>(3.5 , 5.1)	7<br>(5 , 9)	6.8<br>(5.2 , 8.9)	56.4<br>(17.8 , 108.5)
**Senegal**	132<br>(102 , 164)	4.4<br>(3.5 , 5.5)	240<br>(153 , 320)	3.4<br>(2.2 , 4.5)	-23.2<br>(-47 , 3.9)
**Sierra Leone**	82<br>(54 , 163)	4.5<br>(3 , 9)	97<br>(62 , 132)	2.9<br>(1.8 , 3.8)	-36.9<br>(-77.2 , 3)
**Togo**	53<br>(42 , 72)	4.7<br>(3.7 , 6.4)	106<br>(62 , 145)	3.1<br>(1.9 , 4.1)	-34.2<br>(-67.9 , -5.2)
**Eastern Sub-Saharan Africa**	2184<br>(1709 , 2781)	3.2<br>(2.5 , 4.1)	4624<br>(3952 , 5438)	3.2<br>(2.7 , 3.7)	-1.6<br>(-24.9 , 21.7)
**Burundi**	78<br>(56 , 112)	3.5<br>(2.6 , 5)	107<br>(76 , 149)	2.6<br>(1.9 , 3.6)	-25.5<br>(-57.6 , 10.8)
**Comoros**	5<br>(3 , 8)	2.6<br>(1.5 , 3.7)	13<br>(8 , 17)	2.7<br>(1.8 , 3.7)	3.1<br>(-22.9 , 61.4)
**Djibouti**	4<br>(3 , 5)	3.1<br>(2.3 , 4)	19<br>(13 , 28)	3.7<br>(2.7 , 5.1)	20.7<br>(-9 , 62.8)
**Eritrea**	24<br>(17 , 34)	2.7<br>(1.9 , 4)	77<br>(58 , 103)	3.3<br>(2.4 , 4.3)	20.3<br>(-15.2 , 68.6)
**Ethiopia**	533<br>(341 , 793)	2.9<br>(1.8 , 4.3)	1058<br>(740 , 1382)	2.9<br>(2 , 3.7)	-2.8<br>(-30.9 , 29.9)
**Kenya**	102<br>(70 , 132)	1.4<br>(0.9 , 1.8)	375<br>(304 , 459)	1.9<br>(1.6 , 2.3)	40.9<br>(-2.8 , 89.7)
**Madagascar**	141<br>(100 , 265)	3<br>(2.1 , 5.6)	234<br>(175 , 312)	2.4<br>(1.9 , 3.2)	-18.6<br>(-56.1 , 23.7)
**Malawi**	320<br>(268 , 378)	9.1<br>(7.7 , 10.6)	625<br>(361 , 840)	9.3<br>(5.5 , 12.3)	2.3<br>(-43.9 , 40)

**Mozambique**	**171 (106 , 318)**	**3.3 (2.1 , 6.1)**	**336 (252 , 430)**	**3.4 (2.6 , 4.3)**	**4.6 (-48.4 , 67)**
**Rwanda**	**95 (75 , 119)**	**3.6 (2.8 , 4.5)**	**163 (126 , 209)**	**3 (2.4 , 3.8)**	**-15.5 (-39 , 16.4)**
**Somalia**	**60 (40 , 92)**	**2.7 (1.8 , 4.2)**	**143 (84 , 243)**	**2.4 (1.5 , 4.1)**	**-10.6 (-36.4 , 19.6)**
**South Sudan**	**68 (48 , 95)**	**3.1 (2.2 , 4.4)**	**95 (63 , 146)**	**2.9 (2 , 4.3)**	**-6.9 (-31.4 , 25.6)**
**United Republic of Tanzania**	**329 (248 , 448)**	**3.3 (2.5 , 4.5)**	**729 (556 , 961)**	**3.2 (2.5 , 4.2)**	**-2.2 (-34.9 , 29.5)**
**Uganda**	**161 (132 , 190)**	**2.8 (2.3 , 3.2)**	**415 (318 , 515)**	**3.3 (2.5 , 4)**	**19.5 (-11.3 , 60.2)**
**Zambia**	**92 (75 , 113)**	**3.6 (2.9 , 4.4)**	**230 (173 , 305)**	**3.8 (2.9 , 4.9)**	**4.2 (-21 , 39.5)**
**Central Sub-Saharan Africa**	**860 (477 , 1275)**	**4.5 (2.4 , 6.8)**	**1749 (1111 , 2580)**	**3.8 (2.4 , 5.6)**	**-16.7 (-36.3 , 13.9)**
**Angola**	**123 (74 , 228)**	**3.7 (2.2 , 7.1)**	**368 (253 , 643)**	**3.9 (2.6 , 6.8)**	**3.5 (-20.3 , 47.4)**
**Central African Republic**	**38 (27 , 52)**	**3.7 (2.5 , 4.9)**	**55 (38 , 77)**	**2.9 (2 , 3.9)**	**-20.8 (-39.4 , 3.6)**
**Congo**	**47 (34 , 63)**	**4.9 (3.5 , 6.9)**	**106 (75 , 160)**	**4.7 (3.4 , 7)**	**-5.2 (-29.6 , 28.8)**
**Democratic Republic of the Congo**	**615 (310 , 921)**	**4.8 (2.3 , 7.2)**	**1142 (662 , 1642)**	**3.6 (2.1 , 5.3)**	**-23.4 (-43.6 , 8.7)**
**Equatorial Guinea**	**5 (3 , 8)**	**3.1 (1.9 , 4.3)**	**20 (13 , 28)**	**4.7 (3.1 , 6.5)**	**52.3 (3.2 , 147.1)**
**Gabon**	**30 (19 , 53)**	**5.8 (3.6 , 10.2)**	**58 (39 , 106)**	**6.1 (4.2 , 11.1)**	**5.8 (-27.4 , 53.5)**
**SDI: Socio-demographic Index**					
