# Supplementary material for: Global, regional and national burden of bladder cancer and its attributable risk factors in 204 countries and territories, 1990–2019: a systematic analysis for the Global Burden of Disease study 2019
Source: BMJ Glob Health. 2021 Nov 29;6(11):e004128. doi: 10.1136/bmjgh-2020-004128 (PMC8634015; doi:10.1136/bmjgh-2020-004128)
Supplement: Supplementary data [file bmjgh-2020-004128supp004.pdf]

Appendix Table 4: Deaths of bladder cancer and percentage change in age-standardised rates by location, 1990–2019					
	1990		2019		Percentage change in age-standardized rates between 1990 and 2019
	Counts (95% UI)	Rate (95% UI)	Counts (95% UI)	Rate (95% UI)	
Global	121500 (114751 , 127171)	3.5 (3.3 , 3.7)	228735 (210743 , 243193)	2.9 (2.7 , 3.1)	-15.7 (-21 , -8.6)
High-income North America	13773 (12942 , 14275)	3.7 (3.5 , 3.9)	25758 (23429 , 27451)	3.8 (3.5 , 4)	2.1 (-2.3 , 6.6)
Canada	1646 (1550 , 1721)	5.1 (4.8 , 5.3)	2988 (2641 , 3333)	4 (3.6 , 4.4)	-21.2 (-28.1 , -13.3)
Greenland	2 (1 , 2)	6.1 (5.5 , 6.9)	3 (2 , 3)	4.6 (3.8 , 5.7)	-24.1 (-39.2 , -6.4)
United States of America	12125 (11384 , 12576)	3.6 (3.4 , 3.7)	22767 (20719 , 24317)	3.8 (3.5 , 4)	5.2 (0.6 , 10)
Australasia	916 (862 , 959)	3.9 (3.7 , 4.1)	1626 (1425 , 1813)	3 (2.6 , 3.3)	-24.6 (-31.4 , -17.1)
Australia	756 (714 , 793)	3.9 (3.7 , 4.1)	1373 (1198 , 1546)	3 (2.6 , 3.3)	-24.3 (-31.9 , -15.7)
New Zealand	159 (147 , 172)	4 (3.7 , 4.4)	253 (218 , 284)	3 (2.6 , 3.3)	-26.1 (-34.2 , -16.8)
High-income Asia Pacific	5033 (4724 , 5207)	2.8 (2.6 , 2.9)	12876 (10678 , 14233)	2.3 (1.9 , 2.5)	-18.6 (-26.2 , -13.3)
Brunei Darussalam	3 (3 , 4)	4.9 (4 , 6.4)	7 (6 , 9)	4.1 (3.6 , 4.8)	-15.5 (-38.2 , 8.7)
Japan	4319 (4022 , 4477)	2.7 (2.5 , 2.8)	10789 (8798 , 12043)	2.3 (1.9 , 2.5)	-17.2 (-25.2 , -11.4)
Singapore	49 (45 , 53)	2.7 (2.4 , 2.9)	105 (89 , 122)	1.5 (1.2 , 1.7)	-44.9 (-53.5 , -35.8)
Republic of Korea	662 (619 , 728)	2.9 (2.7 , 3.2)	1974 (1724 , 2226)	2.3 (2 , 2.6)	-19.5 (-29 , -8.1)
Western Europe	36844 (35128 , 37885)	6.1 (5.8 , 6.3)	50511 (45163 , 54465)	4.8 (4.3 , 5.1)	-21.6 (-26.1 , -17.1)
Andorra	2 (2 , 3)	5.2 (4 , 6.8)	6 (5 , 7)	4 (3.1 , 5)	-21.8 (-44.9 , 10.1)
Austria	586 (552 , 618)	4.7 (4.4 , 4.9)	716 (632 , 800)	3.5 (3.1 , 3.9)	-25 (-31.8 , -17.4)
Belgium	1079 (1013 , 1137)	6.8 (6.3 , 7.1)	1295 (1144 , 1438)	4.9 (4.4 , 5.4)	-27.5 (-34.7 , -19.7)
Cyprus	36	5.2	92	4.8	-6.9

	(30 , 41)	(4.4 , 5.9)	(78 , 106)	(4.1 , 5.5)	(-24.9 , 13.8)
**Denmark**	488<br>(461 , 512)	5.7<br>(5.4 , 6)	717<br>(631 , 809)	5.7<br>(5 , 6.4)	0.5<br>(-10.5 , 14.1)
**Finland**	267<br>(252 , 280)	3.7<br>(3.4 , 3.9)	371<br>(320 , 420)	2.6<br>(2.3 , 3)	-28.2<br>(-36.6 , -18.3)
**France**	5359<br>(5018 , 5641)	6.1<br>(5.7 , 6.4)	7768<br>(6671 , 8851)	4.8<br>(4.2 , 5.5)	-21.1<br>(-29 , -11.6)
**Germany**	7237<br>(6800 , 7608)	5.4<br>(5.1 , 5.7)	8836<br>(7808 , 9856)	4<br>(3.6 , 4.4)	-26.3<br>(-33.7 , -18.7)
**Greece**	1175<br>(1098 , 1240)	7.7<br>(7.2 , 8.1)	1745<br>(1564 , 1920)	6.2<br>(5.7 , 6.8)	-19.2<br>(-25.8 , -12.1)
**Iceland**	15<br>(13 , 16)	4.9<br>(4.4 , 5.4)	21<br>(18 , 25)	3.5<br>(3 , 4)	-27.7<br>(-38.8 , -15.2)
**Ireland**	176<br>(164 , 187)	4.3<br>(4 , 4.5)	288<br>(246 , 335)	3.7<br>(3.1 , 4.3)	-13.9<br>(-26.5 , 1)
**Israel**	229<br>(210 , 249)	4.9<br>(4.5 , 5.3)	555<br>(485 , 622)	4.5<br>(3.9 , 5)	-8.8<br>(-18.4 , 2.5)
**Italy**	6504<br>(6271 , 6720)	7.1<br>(6.8 , 7.3)	8838<br>(7840 , 9641)	5.2<br>(4.6 , 5.6)	-27.2<br>(-32.3 , -22.9)
**Luxembourg**	32<br>(29 , 35)	5.8<br>(5.3 , 6.3)	47<br>(39 , 55)	4.3<br>(3.6 , 5.1)	-26<br>(-37.2 , -14.1)
**Malta**	25<br>(23 , 28)	6.1<br>(5.5 , 6.8)	41<br>(34 , 48)	4.1<br>(3.4 , 4.8)	-33.5<br>(-44.1 , -20.2)
**Monaco**	6<br>(5 , 7)	7.6<br>(6 , 9.4)	10<br>(8 , 19)	9.4<br>(6.9 , 16.9)	24.7<br>(-9.6 , 104.3)
**Netherlands**	1213<br>(1142 , 1273)	5.9<br>(5.6 , 6.2)	1984<br>(1760 , 2203)	5.4<br>(4.8 , 5.9)	-9<br>(-17.5 , 0.8)
**Norway**	410<br>(387 , 428)	5.5<br>(5.2 , 5.7)	444<br>(390 , 491)	4.2<br>(3.7 , 4.6)	-24.6<br>(-31 , -18)
**Portugal**	685<br>(644 , 722)	5.1<br>(4.8 , 5.4)	1229<br>(1083 , 1369)	4.4<br>(4 , 4.9)	-12.9<br>(-22.1 , -3.3)
**San Marino**	3<br>(2 , 3)	8.5<br>(7.2 , 9.9)	6<br>(4 , 8)	8<br>(5.5 , 11.1)	-6<br>(-36.5 , 33)
**Spain**	4060<br>(3815 , 4264)	7.3<br>(6.9 , 7.7)	6580<br>(5750 , 7400)	5.9<br>(5.2 , 6.5)	-19.7<br>(-27.2 , -10.5)
**Sweden**	647<br>(601 , 684)	3.9<br>(3.6 , 4.1)	964<br>(858 , 1059)	4<br>(3.6 , 4.3)	1.4<br>(-6.7 , 9.8)
**Switzerland**	315<br>(293 , 335)	2.8<br>(2.6 , 3)	694<br>(597 , 780)	3.5<br>(3 , 3.9)	23.5<br>(10.3 , 38.9)

**United Kingdom**	**6264 (5985 , 6425)**	**6.6 (6.3 , 6.7)**	**7220 (6546 , 7650)**	**5.1 (4.6 , 5.4)**	**-22.5 (-26.5 , -19.2)**
**Southern Latin America**	**2035 (1943 , 2118)**	**4.6 (4.4 , 4.8)**	**3068 (2812 , 3304)**	**3.6 (3.3 , 3.9)**	**-22.4 (-28.5 , -15.9)**
**Argentina**	**1528 (1448 , 1609)**	**4.9 (4.6 , 5.2)**	**2076 (1894 , 2275)**	**3.7 (3.4 , 4.1)**	**-23.8 (-30.5 , -16.1)**
**Chile**	**268 (248 , 287)**	**3 (2.7 , 3.2)**	**695 (611 , 769)**	**2.9 (2.5 , 3.2)**	**-3.2 (-14.7 , 9.3)**
**Uruguay**	**239 (223 , 255)**	**6 (5.7 , 6.4)**	**297 (265 , 331)**	**5 (4.5 , 5.5)**	**-17.4 (-27 , -8)**
**Eastern Europe**	**8581 (8134 , 9129)**	**3.1 (2.9 , 3.3)**	**10932 (9698 , 12205)**	**3.1 (2.8 , 3.5)**	**-0.1 (-10.6 , 12.4)**
**Belarus**	**403 (370 , 441)**	**3.1 (2.9 , 3.4)**	**421 (329 , 536)**	**2.6 (2 , 3.3)**	**-17.8 (-35.3 , 5.7)**
**Estonia**	**77 (72 , 83)**	**3.8 (3.5 , 4.1)**	**114 (90 , 142)**	**3.8 (3 , 4.8)**	**1.2 (-20.6 , 29.9)**
**Latvia**	**137 (126 , 147)**	**3.8 (3.5 , 4.1)**	**207 (166 , 256)**	**4.7 (3.8 , 5.9)**	**24.1 (-1.9 , 54.9)**
**Lithuania**	**191 (179 , 205)**	**4.3 (4 , 4.6)**	**262 (211 , 320)**	**4.1 (3.3 , 5.1)**	**-3.5 (-23.6 , 18.7)**
**Republic of Moldova**	**135 (123 , 148)**	**3.2 (2.9 , 3.5)**	**172 (145 , 206)**	**2.9 (2.5 , 3.5)**	**-7.7 (-22.1 , 8)**
**Russian Federation**	**5631 (5453 , 5835)**	**3.2 (3.1 , 3.3)**	**7184 (6116 , 8254)**	**3 (2.6 , 3.5)**	**-4.8 (-18 , 8.9)**
**Ukraine**	**2007 (1707 , 2413)**	**2.8 (2.4 , 3.4)**	**2571 (2114 , 3080)**	**3.3 (2.7 , 4)**	**17.9 (-11.1 , 53.1)**
**Central Europe**	**6804 (6573 , 6983)**	**4.7 (4.6 , 4.9)**	**11877 (10466 , 13441)**	**5.3 (4.7 , 6)**	**11.6 (-1.1 , 25.4)**
**Albania**	**22 (20 , 24)**	**1.2 (1.1 , 1.3)**	**46 (35 , 60)**	**1.1 (0.8 , 1.4)**	**-12.2 (-34.4 , 14)**
**Bosnia and Herzegovina**	**119 (109 , 127)**	**3.3 (3 , 3.5)**	**293 (232 , 362)**	**4.8 (3.9 , 6)**	**46.9 (16 , 81.7)**
**Bulgaria**	**425 (396 , 455)**	**3.5 (3.3 , 3.7)**	**713 (565 , 878)**	**4.7 (3.8 , 5.8)**	**35.5 (7.3 , 68)**
**Croatia**	**292 (271 , 314)**	**4.9 (4.5 , 5.2)**	**477 (380 , 588)**	**5 (4 , 6.2)**	**2.8 (-18.9 , 28.5)**
**Czechia**	**727 (687 , 762)**	**5.2 (5 , 5.5)**	**977 (791 , 1173)**	**4.4 (3.5 , 5.2)**	**-16.6 (-33.3 , 1.5)**
**Hungary**	**790**	**5.5**	**1055**	**5.2**	**-4.7**

	(750 , 829)	(5.2 , 5.7)	(872 , 1263)	(4.3 , 6.2)	(-21.4 , 14)
Montenegro	24<br>(21 , 28)	4.1<br>(3.5 , 4.8)	44<br>(36 , 53)	4.5<br>(3.7 , 5.4)	9.2<br>(-15.7 , 44.5)
North Macedonia	81<br>(71 , 90)	4.7<br>(4.1 , 5.2)	176<br>(140 , 219)	5.7<br>(4.6 , 7.1)	23.1<br>(-4.5 , 57)
Poland	2372<br>(2275 , 2444)	5.5<br>(5.2 , 5.7)	4694<br>(3946 , 5633)	6.4<br>(5.4 , 7.7)	16.6<br>(-2.5 , 40.1)
Romania	1037<br>(986 , 1089)	3.8<br>(3.6 , 3.9)	1849<br>(1514 , 2256)	4.8<br>(3.9 , 5.8)	26.2<br>(3.9 , 53.9)
Serbia	539<br>(449 , 623)	5.1<br>(4.3 , 5.9)	954<br>(762 , 1184)	5.9<br>(4.8 , 7.3)	15.3<br>(-11 , 50.1)
Slovakia	270<br>(253 , 288)	4.5<br>(4.2 , 4.8)	396<br>(317 , 493)	4.2<br>(3.4 , 5.3)	-6.5<br>(-25.2 , 16.4)
Slovenia	107<br>(84 , 134)	4.4<br>(3.5 , 5.5)	202<br>(157 , 257)	4.2<br>(3.2 , 5.3)	-5.3<br>(-31.7 , 28.6)
Central Asia	964<br>(838 , 1116)	2.2<br>(1.9 , 2.5)	1605<br>(1443 , 1773)	2.6<br>(2.3 , 2.8)	17.9<br>(1.8 , 42.7)
Armenia	129<br>(109 , 145)	5.3<br>(4.5 , 6)	215<br>(178 , 257)	5.2<br>(4.3 , 6.2)	-1.2<br>(-22.2 , 27.2)
Azerbaijan	108<br>(92 , 124)	2.2<br>(1.9 , 2.5)	218<br>(178 , 269)	2.7<br>(2.2 , 3.3)	22.8<br>(-6.7 , 56.7)
Georgia	187<br>(156 , 218)	3.1<br>(2.6 , 3.6)	277<br>(231 , 324)	4.5<br>(3.8 , 5.3)	45.5<br>(17.2 , 81.9)
Kazakhstan	282<br>(230 , 360)	2.4<br>(1.9 , 3)	364<br>(305 , 429)	2.3<br>(1.9 , 2.7)	-2.1<br>(-23.7 , 27.7)
Kyrgyzstan	51<br>(45 , 57)	1.7<br>(1.5 , 1.9)	61<br>(51 , 71)	1.5<br>(1.3 , 1.7)	-12.9<br>(-28.4 , 5.4)
Mongolia	22<br>(18 , 26)	2.3<br>(1.9 , 2.6)	25<br>(19 , 31)	1.4<br>(1.1 , 1.7)	-39.8<br>(-53.7 , -22.2)
Tajikistan	34<br>(23 , 44)	1.3<br>(0.9 , 1.7)	60<br>(49 , 73)	1.7<br>(1.4 , 2.1)	35.7<br>(-2.6 , 106.5)
Turkmenistan	16<br>(14 , 18)	1<br>(0.8 , 1.1)	50<br>(39 , 63)	1.4<br>(1.1 , 1.7)	42.5<br>(11.3 , 83.5)
Uzbekistan	136<br>(98 , 200)	1.3<br>(0.9 , 1.9)	335<br>(276 , 400)	2.1<br>(1.7 , 2.4)	64.7<br>(11.2 , 142)
Central Latin America	1241<br>(1171 , 1294)	1.7<br>(1.6 , 1.8)	3399<br>(2928 , 3953)	1.5<br>(1.3 , 1.8)	-11.7<br>(-24 , 2.6)
Colombia	318<br>(295 , 342)	2.1<br>(1.9 , 2.3)	722<br>(553 , 915)	1.3<br>(1 , 1.7)	-36.2<br>(-51 , -19.3)

**Costa Rica**	**36 (33 , 39)**	**2.2 (2 , 2.4)**	**106 (82 , 134)**	**2.1 (1.6 , 2.7)**	**-5.9 (-27.2 , 19.6)**
**El Salvador**	**27 (25 , 30)**	**1 (0.9 , 1.1)**	**69 (53 , 88)**	**1.1 (0.9 , 1.4)**	**12.9 (-13.7 , 45.2)**
**Guatemala**	**35 (31 , 40)**	**1.3 (1.1 , 1.4)**	**113 (89 , 141)**	**1.1 (0.9 , 1.4)**	**-10.7 (-31.1 , 13.7)**
**Honduras**	**20 (16 , 24)**	**1.1 (0.9 , 1.3)**	**86 (65 , 124)**	**1.7 (1.3 , 2.4)**	**46.6 (8.6 , 111.2)**
**Mexico**	**609 (576 , 634)**	**1.6 (1.5 , 1.7)**	**1665 (1417 , 1929)**	**1.5 (1.3 , 1.8)**	**-7.6 (-20.2 , 7.1)**
**Nicaragua**	**14 (12 , 16)**	**1.1 (0.9 , 1.2)**	**45 (38 , 53)**	**1.2 (1 , 1.4)**	**16.2 (-6.6 , 50.3)**
**Panama**	**22 (20 , 25)**	**1.6 (1.4 , 1.8)**	**55 (41 , 70)**	**1.3 (1 , 1.7)**	**-18.4 (-38.2 , 5)**
**Venezuela (Bolivarian Republic of)**	**159 (147 , 172)**	**1.9 (1.7 , 2)**	**537 (409 , 682)**	**2 (1.5 , 2.5)**	**5.9 (-18.9 , 35.3)**
**Andean Latin America**	**324 (290 , 361)**	**1.8 (1.6 , 2)**	**874 (716 , 1054)**	**1.6 (1.3 , 2)**	**-8.4 (-25.8 , 12.7)**
**Bolivia (Plurinational State of)**	**69 (55 , 84)**	**2.5 (2 , 3)**	**207 (163 , 257)**	**2.7 (2.2 , 3.3)**	**7.8 (-16.9 , 41.4)**
**Ecuador**	**76 (70 , 83)**	**1.6 (1.5 , 1.8)**	**237 (189 , 295)**	**1.7 (1.4 , 2.2)**	**5.5 (-17.2 , 30.7)**
**Peru**	**179 (153 , 208)**	**1.7 (1.4 , 1.9)**	**431 (321 , 570)**	**1.3 (1 , 1.8)**	**-19.2 (-41 , 10.9)**
**Caribbean**	**727 (674 , 774)**	**3 (2.7 , 3.2)**	**1519 (1313 , 1745)**	**2.9 (2.5 , 3.4)**	**-1.5 (-14.8 , 13.5)**
**Antigua and Barbuda**	**1 (1 , 2)**	**2.5 (2.3 , 2.8)**	**3 (2 , 3)**	**2.9 (2.5 , 3.4)**	**17.2 (-2.4 , 39.2)**
**Barbados**	**8 (7 , 8)**	**2.4 (2.2 , 2.7)**	**14 (11 , 16)**	**2.7 (2.3 , 3.2)**	**14 (-6.4 , 35.8)**
**Belize**	**2 (1 , 2)**	**1.7 (1.5 , 1.9)**	**5 (5 , 6)**	**2.1 (1.8 , 2.5)**	**25.1 (4.6 , 48.9)**
**Bermuda**	**4 (3 , 4)**	**6.7 (6 , 7.4)**	**7 (5 , 8)**	**4.7 (3.9 , 5.6)**	**-29.5 (-42.4 , -13.1)**
**Bahamas**	**3 (2 , 3)**	**1.9 (1.7 , 2.1)**	**6 (5 , 8)**	**1.8 (1.5 , 2.2)**	**-1.6 (-20.1 , 21.4)**
**Cuba**	**383**	**3.8**	**798**	**4**	**6.2**

	(357 , 407)	(3.5 , 4)	(652 , 982)	(3.3 , 5)	(-14.2 , 29.8)
Dominica	3<br>(2 , 3)	3.4<br>(3.1 , 3.8)	4<br>(3 , 4)	3.9<br>(3.3 , 4.8)	14.5<br>(-6.3 , 42)
Dominican Republic	36<br>(30 , 41)	1.2<br>(1 , 1.3)	129<br>(97 , 169)	1.5<br>(1.1 , 1.9)	29.8<br>(-5.3 , 75.8)
Grenada	2<br>(2 , 2)	2.7<br>(2.4 , 3.1)	3<br>(3 , 4)	3.2<br>(2.8 , 3.6)	15.9<br>(-3.1 , 37.1)
Guyana	6<br>(5 , 7)	1.9<br>(1.7 , 2.2)	10<br>(8 , 13)	1.9<br>(1.5 , 2.4)	-0.6<br>(-24 , 28.4)
Haiti	86<br>(58 , 114)	3.2<br>(2.1 , 4.2)	166<br>(105 , 243)	2.9<br>(1.9 , 4.2)	-7.8<br>(-30.6 , 21.3)
Jamaica	48<br>(43 , 54)	2.6<br>(2.4 , 2.9)	72<br>(57 , 89)	2.3<br>(1.8 , 2.9)	-11.6<br>(-30.8 , 10.5)
Puerto Rico	93<br>(84 , 101)	2.7<br>(2.4 , 2.9)	193<br>(149 , 245)	2.4<br>(1.8 , 3)	-10.7<br>(-31.2 , 13.9)
Saint Kitts and Nevis	2<br>(1 , 2)	4.1<br>(3.7 , 4.5)	2<br>(2 , 2)	3.6<br>(3.1 , 4.2)	-12.7<br>(-26.4 , 5.3)
Saint Lucia	3<br>(3 , 3)	3.9<br>(3.6 , 4.3)	7<br>(6 , 8)	3.5<br>(2.9 , 4.1)	-11.5<br>(-26.5 , 5.7)
Saint Vincent and the Grenadines	2<br>(2 , 2)	2.5<br>(2.3 , 2.8)	4<br>(3 , 4)	2.8<br>(2.5 , 3.3)	13.4<br>(-2.9 , 32.9)
Suriname	4<br>(4 , 4)	1.7<br>(1.5 , 1.9)	11<br>(9 , 13)	1.9<br>(1.6 , 2.3)	12.3<br>(-7.8 , 36.4)
Trinidad and Tobago	16<br>(15 , 18)	2.1<br>(1.9 , 2.3)	30<br>(23 , 39)	1.7<br>(1.3 , 2.2)	-21.6<br>(-42.2 , 3.1)
United States Virgin Islands	1<br>(1 , 1)	1.7<br>(1.3 , 2)	4<br>(3 , 5)	2.2<br>(1.8 , 2.6)	29.9<br>(-0.7 , 73.6)
Tropical Latin America	2190<br>(2072 , 2275)	2.8<br>(2.6 , 3)	5639<br>(5121 , 6032)	2.4<br>(2.2 , 2.6)	-13.6<br>(-19.6 , -7.7)
Brazil	2166<br>(2048 , 2251)	2.9<br>(2.7 , 3)	5566<br>(5057 , 5951)	2.5<br>(2.2 , 2.6)	-14.3<br>(-20.1 , -8.3)
Paraguay	24<br>(21 , 28)	1.2<br>(1 , 1.4)	73<br>(56 , 93)	1.4<br>(1.1 , 1.8)	17.3<br>(-14.1 , 59)
East Asia	18073<br>(15872 , 20271)	2.6<br>(2.3 , 2.9)	42159<br>(36023 , 49346)	2.3<br>(1.9 , 2.6)	-12.3<br>(-27.9 , 7.3)
China	17294<br>(15094 , 19447)	2.6<br>(2.3 , 2.9)	40094<br>(33977 , 47183)	2.2<br>(1.9 , 2.6)	-13.3<br>(-29.1 , 7.3)
Democratic People's Republic	274	2	542	1.8	-12.3

of Korea	(203 , 351)	(1.5 , 2.6)	(458 , 639)	(1.5 , 2.1)	(-31.7 , 17.9)
Taiwan (Province of China)	506 (481 , 530)	3.7 (3.5 , 3.9)	1523 (1190 , 1965)	3.8 (3 , 4.9)	2.4 (-20.2 , 31.4)
Southeast Asia	3871 (3412 , 4277)	1.8 (1.6 , 2)	9027 (7875 , 10499)	1.8 (1.5 , 2)	-3.9 (-17.9 , 13.5)
Cambodia	74 (56 , 96)	2 (1.5 , 2.5)	198 (150 , 245)	2 (1.5 , 2.4)	-0.5 (-24.9 , 26.9)
Indonesia	1247 (1010 , 1494)	1.6 (1.3 , 1.9)	3157 (2377 , 4469)	1.8 (1.4 , 2.6)	17.5 (-9.3 , 47.6)
Lao People's Democratic Republic	39 (27 , 52)	2.2 (1.5 , 2.9)	66 (49 , 82)	1.9 (1.4 , 2.3)	-14.9 (-34.5 , 12.4)
Malaysia	233 (187 , 274)	3 (2.4 , 3.6)	686 (545 , 859)	3.1 (2.5 , 3.9)	2.6 (-25 , 49.4)
Maldives	2 (1 , 2)	2.8 (2.3 , 3.6)	5 (4 , 6)	2.1 (1.7 , 2.6)	-26.5 (-47 , 1.3)
Mauritius	21 (20 , 23)	3.3 (3 , 3.5)	33 (27 , 41)	2 (1.6 , 2.5)	-37.6 (-50.5 , -22.4)
Myanmar	423 (290 , 558)	2.1 (1.5 , 2.8)	704 (580 , 864)	1.8 (1.5 , 2.1)	-17.4 (-37.3 , 14.4)
Philippines	333 (286 , 377)	1.4 (1.2 , 1.5)	757 (624 , 906)	1.1 (0.9 , 1.3)	-16.7 (-33.9 , 4.6)
Sri Lanka	103 (91 , 117)	1.2 (1.1 , 1.4)	308 (233 , 399)	1.4 (1 , 1.7)	13.3 (-14.9 , 51.6)
Seychelles	3 (2 , 3)	5 (4.3 , 5.7)	5 (4 , 5)	4.8 (4.2 , 5.6)	-2.2 (-17.7 , 15.8)
Thailand	834 (728 , 954)	2.9 (2.5 , 3.3)	1625 (1231 , 2090)	1.7 (1.3 , 2.1)	-42.1 (-57.7 , -22.7)
Timor-Leste	3 (2 , 5)	1.5 (1 , 2.1)	11 (8 , 15)	1.6 (1.2 , 2.1)	8.3 (-21.9 , 61.4)
Viet Nam	550 (443 , 656)	1.5 (1.2 , 1.8)	1461 (1160 , 1776)	1.8 (1.4 , 2.2)	19.6 (-7.5 , 61.3)
Oceania	37 (29 , 46)	1.5 (1.2 , 1.9)	105 (81 , 134)	1.8 (1.4 , 2.3)	19.1 (-1.1 , 42.8)
American Samoa	0 (0 , 0)	1.8 (1.6 , 2)	1 (1 , 1)	2.3 (2 , 2.7)	30.8 (7.4 , 56.7)
Cook Islands	0 (0 , 0)	3.3 (2.8 , 3.9)	1 (1 , 1)	3 (2.5 , 3.6)	-8.3 (-29.3 , 14.8)
Micronesia (Federated States	1	2.3	2	2.7	16.9

**of)**	**(1 , 1)**	**(1.8 , 2.9)**	**(1 , 2)**	**(2.1 , 3.4)**	**(-14.3 , 56.5)**
**Fiji**	**5<br>(4 , 6)**	**1.8<br>(1.5 , 2.1)**	**13<br>(10 , 16)**	**2.2<br>(1.7 , 2.6)**	**21.9<br>(-9.3 , 63.7)**
**Guam**	**1<br>(1 , 1)**	**1.8<br>(1.6 , 2.1)**	**3<br>(3 , 4)**	**1.7<br>(1.4 , 2)**	**-9.2<br>(-26.4 , 13.5)**
**Kiribati**	**1<br>(0 , 1)**	**1.8<br>(1.5 , 2.1)**	**1<br>(1 , 1)**	**1.8<br>(1.4 , 2.1)**	**-0.5<br>(-21.2 , 22.9)**
**Marshall Islands**	**0<br>(0 , 0)**	**2.4<br>(1.8 , 3)**	**1<br>(1 , 1)**	**2.7<br>(2.1 , 3.4)**	**12<br>(-13.4 , 46.2)**
**Nauru**	**0<br>(0 , 0)**	**2.7<br>(2.1 , 3.3)**	**0<br>(0 , 0)**	**2.9<br>(2.3 , 3.6)**	**8.7<br>(-12.5 , 36.1)**
**Niue**	**0<br>(0 , 0)**	**2<br>(1.7 , 2.4)**	**0<br>(0 , 0)**	**2.2<br>(1.8 , 2.6)**	**8.7<br>(-14.3 , 35.4)**
**Northern Mariana Islands**	**0<br>(0 , 0)**	**1.5<br>(1.3 , 1.8)**	**1<br>(1 , 1)**	**2.8<br>(2.3 , 3.3)**	**81.8<br>(45.3 , 120.9)**
**Palau**	**0<br>(0 , 0)**	**1<br>(0.8 , 1.3)**	**0<br>(0 , 0)**	**1<br>(0.8 , 1.3)**	**-0.1<br>(-24.2 , 28.4)**
**Papua New Guinea**	**20<br>(14 , 27)**	**1.3<br>(0.9 , 1.8)**	**63<br>(44 , 87)**	**1.7<br>(1.2 , 2.2)**	**25.9<br>(-1.8 , 56.9)**
**Samoa**	**2<br>(1 , 2)**	**2.2<br>(1.9 , 2.7)**	**3<br>(2 , 4)**	**2.2<br>(1.8 , 2.7)**	**-1.2<br>(-20.9 , 26.1)**
**Solomon Islands**	**2<br>(2 , 4)**	**2.1<br>(1.5 , 3)**	**7<br>(5 , 9)**	**2.6<br>(1.9 , 3.3)**	**19.5<br>(-8.8 , 56.9)**
**Tokelau**	**0<br>(0 , 0)**	**1.9<br>(1.5 , 2.4)**	**0<br>(0 , 0)**	**1.9<br>(1.5 , 2.5)**	**4.8<br>(-20.6 , 37)**
**Tonga**	**1<br>(1 , 1)**	**1.5<br>(1.2 , 1.9)**	**1<br>(1 , 2)**	**1.8<br>(1.4 , 2.3)**	**20.3<br>(-4.8 , 53.7)**
**Tuvalu**	**0<br>(0 , 0)**	**2.1<br>(1.7 , 2.7)**	**0<br>(0 , 0)**	**2.2<br>(1.7 , 2.8)**	**5.8<br>(-20 , 39.1)**
**Vanuatu**	**1<br>(1 , 1)**	**1.6<br>(1.2 , 2.2)**	**3<br>(2 , 4)**	**2.1<br>(1.6 , 2.8)**	**30.7<br>(-2.8 , 76.1)**
**North Africa and Middle East**	**6306<br>(5359 , 7209)**	**4.2<br>(3.5 , 4.8)**	**15463<br>(13175 , 18420)**	**4<br>(3.5 , 4.8)**	**-2.5<br>(-22.4 , 32.6)**
**Afghanistan**	**263<br>(161 , 379)**	**4.3<br>(2.6 , 6)**	**366<br>(254 , 492)**	**3.8<br>(2.6 , 4.9)**	**-12.2<br>(-35.3 , 18.6)**
**Algeria**	**253<br>(201 , 320)**	**3<br>(2.5 , 3.7)**	**601<br>(478 , 745)**	**2.3<br>(1.9 , 2.8)**	**-24<br>(-44.5 , 2.1)**
**Bahrain**	**9<br>(8 , 11)**	**8.4<br>(7.1 , 9.9)**	**25<br>(19 , 32)**	**5.1<br>(4.1 , 6.3)**	**-38.7<br>(-53.8 , -18.5)**

**Egypt**	**2109 (1920 , 2315)**	**7.2 (6.5 , 7.8)**	**4845 (3186 , 7103)**	**7.8 (5.2 , 11.4)**	**9.4 (-28.8 , 61.1)**
**Iran (Islamic Republic of)**	**510 (403 , 596)**	**2.5 (2 , 3)**	**1672 (1523 , 1828)**	**2.6 (2.3 , 2.8)**	**1.9 (-15.9 , 37.2)**
**Iraq**	**367 (265 , 473)**	**5.3 (3.8 , 6.8)**	**1265 (996 , 1527)**	**6.9 (5.5 , 8.1)**	**29.3 (-6.4 , 94.4)**
**Jordan**	**41 (33 , 50)**	**4 (3.3 , 4.9)**	**179 (143 , 224)**	**3.6 (2.9 , 4.5)**	**-9.8 (-32.2 , 27)**
**Kuwait**	**18 (15 , 20)**	**4 (3.4 , 4.5)**	**70 (57 , 85)**	**3.7 (3 , 4.5)**	**-6 (-24.7 , 19.4)**
**Lebanon**	**231 (189 , 281)**	**12.2 (10.1 , 14.7)**	**536 (418 , 707)**	**10.4 (8.1 , 13.7)**	**-15.2 (-37.8 , 24.5)**
**Libya**	**87 (62 , 116)**	**5.4 (3.8 , 7.1)**	**233 (172 , 300)**	**5.4 (4 , 6.9)**	**-0.8 (-34.4 , 62.9)**
**Morocco**	**229 (171 , 280)**	**2 (1.4 , 2.4)**	**630 (482 , 795)**	**2.4 (1.8 , 3)**	**21.4 (-10.7 , 66.4)**
**Palestine**	**31 (21 , 41)**	**4.2 (2.9 , 5.5)**	**67 (56 , 79)**	**3.6 (3 , 4.3)**	**-14 (-39 , 36.3)**
**Oman**	**11 (7 , 15)**	**2.3 (1.5 , 3)**	**27 (22 , 32)**	**2.5 (2.1 , 2.9)**	**9.1 (-24.7 , 76.9)**
**Qatar**	**2 (1 , 3)**	**3.8 (2.7 , 5)**	**14 (10 , 19)**	**5.7 (4.3 , 7.5)**	**52 (6.1 , 130.3)**
**Saudi Arabia**	**90 (58 , 121)**	**1.9 (1.2 , 2.6)**	**203 (161 , 261)**	**1.7 (1.3 , 2.1)**	**-14.7 (-42.2 , 51.2)**
**Sudan**	**292 (140 , 699)**	**3.7 (1.7 , 8.9)**	**493 (327 , 880)**	**3.2 (2.1 , 5.8)**	**-13.1 (-45.4 , 59)**
**Syrian Arab Republic**	**86 (64 , 109)**	**1.9 (1.4 , 2.4)**	**204 (151 , 269)**	**2.1 (1.6 , 2.7)**	**8.8 (-25 , 73.5)**
**Tunisia**	**191 (149 , 236)**	**4.6 (3.6 , 5.7)**	**480 (346 , 662)**	**4.2 (3 , 5.8)**	**-8.1 (-38.7 , 43.3)**
**Turkey**	**1344 (1044 , 1638)**	**4.2 (3.2 , 5)**	**3068 (2460 , 3717)**	**3.7 (2.9 , 4.4)**	**-12.4 (-34.8 , 24.8)**
**United Arab Emirates**	**22 (11 , 53)**	**7.7 (3.5 , 19.7)**	**152 (82 , 263)**	**5.7 (3 , 9.8)**	**-25.8 (-59 , 35.1)**
**Yemen**	**117 (61 , 187)**	**2.9 (1.5 , 4.7)**	**318 (227 , 420)**	**2.9 (2.1 , 3.9)**	**2 (-30.6 , 67.7)**
**South Asia**	**8345 (7111 , 9458)**	**2 (1.7 , 2.3)**	**21990 (19474 , 24707)**	**1.8 (1.6 , 2)**	**-10.7 (-24.9 , 9.3)**
**Bangladesh**	**538**	**1.4**	**1383**	**1.2**	**-10.9**

	(414 , 663)	(1 , 1.7)	(978 , 1858)	(0.9 , 1.6)	(-35.9 , 15.5)
**Bhutan**	**2**<br>**(1 , 3)**	**1.2**<br>**(0.8 , 1.7)**	**7**<br>**(5 , 10)**	**1.5**<br>**(1.1 , 2)**	**29.5**<br>**(-3.9 , 75.8)**
**India**	**4908**<br>**(4193 , 5652)**	**1.5**<br>**(1.3 , 1.7)**	**14507**<br>**(12469 , 16972)**	**1.5**<br>**(1.3 , 1.7)**	**-0.9**<br>**(-17.6 , 20.7)**
**Nepal**	**91**<br>**(58 , 124)**	**1.2**<br>**(0.8 , 1.7)**	**275**<br>**(195 , 357)**	**1.5**<br>**(1 , 1.9)**	**22.1**<br>**(-8.5 , 64.8)**
**Pakistan**	**2806**<br>**(2277 , 3393)**	**5.5**<br>**(4.4 , 6.6)**	**5818**<br>**(4576 , 7412)**	**6.4**<br>**(5 , 8.1)**	**17.1**<br>**(-12.5 , 62.4)**
**Southern Sub-Saharan Africa**	**761**<br>**(676 , 843)**	**3.1**<br>**(2.7 , 3.4)**	**1490**<br>**(1310 , 1660)**	**3**<br>**(2.6 , 3.3)**	**-2.3**<br>**(-17.5 , 15.8)**
**Botswana**	**14**<br>**(10 , 17)**	**2.8**<br>**(2.2 , 3.5)**	**33**<br>**(25 , 43)**	**2.9**<br>**(2.2 , 3.7)**	**4.3**<br>**(-24 , 41.7)**
**Lesotho**	**17**<br>**(13 , 21)**	**1.9**<br>**(1.4 , 2.4)**	**28**<br>**(20 , 37)**	**2.6**<br>**(1.9 , 3.3)**	**37**<br>**(2.6 , 84.7)**
**Namibia**	**12**<br>**(9 , 14)**	**1.8**<br>**(1.5 , 2.1)**	**25**<br>**(20 , 31)**	**2**<br>**(1.6 , 2.4)**	**12.7**<br>**(-14.4 , 46.2)**
**South Africa**	**437**<br>**(377 , 493)**	**2.3**<br>**(2 , 2.6)**	**883**<br>**(795 , 964)**	**2.2**<br>**(2 , 2.4)**	**-2**<br>**(-13 , 13.6)**
**Eswatini**	**6**<br>**(5 , 8)**	**2.5**<br>**(2 , 3.3)**	**13**<br>**(10 , 17)**	**2.7**<br>**(2.1 , 3.5)**	**6.7**<br>**(-19.8 , 41.9)**
**Zimbabwe**	**276**<br>**(240 , 315)**	**8.1**<br>**(7 , 9.1)**	**508**<br>**(331 , 657)**	**8.7**<br>**(5.7 , 11.3)**	**8.3**<br>**(-30.6 , 44.7)**
**Western Sub-Saharan Africa**	**2073**<br>**(1740 , 2489)**	**2.8**<br>**(2.3 , 3.3)**	**3815**<br>**(2655 , 4520)**	**2.5**<br>**(1.7 , 2.9)**	**-10.6**<br>**(-43.4 , 14.3)**
**Benin**	**70**<br>**(55 , 103)**	**3.9**<br>**(3.1 , 5.7)**	**101**<br>**(77 , 126)**	**2.5**<br>**(1.9 , 3)**	**-37.1**<br>**(-64 , -13.5)**
**Burkina Faso**	**138**<br>**(96 , 234)**	**3.8**<br>**(2.7 , 6.5)**	**176**<br>**(118 , 238)**	**2.3**<br>**(1.6 , 3.1)**	**-39.6**<br>**(-73.1 , -12.8)**
**Cameroon**	**150**<br>**(122 , 183)**	**4.1**<br>**(3.4 , 5)**	**314**<br>**(191 , 422)**	**3.2**<br>**(2 , 4.2)**	**-22.6**<br>**(-47.7 , 3.4)**
**Cabo Verde**	**3**<br>**(2 , 3)**	**1.1**<br>**(1 , 1.2)**	**13**<br>**(11 , 15)**	**3.2**<br>**(2.7 , 3.7)**	**190.3**<br>**(139.3 , 251.1)**
**Chad**	**83**<br>**(63 , 105)**	**3.2**<br>**(2.4 , 4.1)**	**137**<br>**(99 , 178)**	**2.9**<br>**(2.1 , 3.7)**	**-10.6**<br>**(-29.6 , 12.5)**
**CÃ´te d'Ivoire**	**135**<br>**(103 , 181)**	**4.5**<br>**(3.5 , 5.9)**	**249**<br>**(189 , 320)**	**2.9**<br>**(2.3 , 3.6)**	**-34.3**<br>**(-58.3 , -10.4)**
**Gambia**	**5**<br>**(4 , 6)**	**1.7**<br>**(1.4 , 2.1)**	**17**<br>**(13 , 22)**	**2**<br>**(1.5 , 2.6)**	**20.3**<br>**(-15.1 , 66.2)**

**Ghana**	**188 (149 , 297)**	**3.5 (2.8 , 5.6)**	**393 (218 , 514)**	**2.7 (1.6 , 3.5)**	**-21.5 (-56.8 , 11.2)**
**Guinea**	**168 (139 , 201)**	**5.8 (4.8 , 6.8)**	**300 (204 , 415)**	**6.2 (4.3 , 8.6)**	**7.9 (-28 , 59.9)**
**Guinea-Bissau**	**15 (11 , 21)**	**4.4 (3.3 , 5.9)**	**19 (14 , 24)**	**3.1 (2.4 , 3.9)**	**-29.8 (-48.6 , -6)**
**Liberia**	**45 (31 , 88)**	**4.6 (3.2 , 9)**	**45 (33 , 67)**	**2.7 (2 , 4)**	**-41.8 (-63.6 , -13.9)**
**Mali**	**350 (294 , 409)**	**10 (8.4 , 11.5)**	**722 (313 , 979)**	**10.1 (4.4 , 13.5)**	**1 (-56.5 , 41.5)**
**Mauritania**	**37 (29 , 47)**	**4.1 (3.3 , 5.2)**	**45 (32 , 63)**	**2.5 (1.8 , 3.5)**	**-38.8 (-55.7 , -16.2)**
**Niger**	**76 (52 , 97)**	**3.4 (2.3 , 4.2)**	**140 (89 , 199)**	**2.3 (1.5 , 3.2)**	**-31.4 (-55.6 , -7.8)**
**Nigeria**	**380 (277 , 495)**	**1 (0.8 , 1.3)**	**805 (643 , 1019)**	**1.2 (1 , 1.5)**	**15.9 (-19.8 , 72.7)**
**Sao Tome and Principe**	**2 (2 , 3)**	**3.8 (3.1 , 4.4)**	**5 (4 , 6)**	**5.2 (4.1 , 6.7)**	**36.2 (3.9 , 76)**
**Senegal**	**113 (88 , 139)**	**4.1 (3.2 , 5)**	**185 (120 , 243)**	**2.9 (1.9 , 3.7)**	**-28.9 (-50 , -5.8)**
**Sierra Leone**	**73 (48 , 144)**	**4.2 (2.8 , 8.3)**	**75 (49 , 100)**	**2.4 (1.6 , 3.2)**	**-42.1 (-78.8 , -7)**
**Togo**	**42 (33 , 57)**	**4.1 (3.3 , 5.6)**	**74 (45 , 100)**	**2.5 (1.5 , 3.3)**	**-39.3 (-69.7 , -14.5)**
**Eastern Sub-Saharan Africa**	**1870 (1455 , 2390)**	**3 (2.3 , 3.8)**	**3628 (3107 , 4238)**	**2.7 (2.3 , 3.2)**	**-8.5 (-29.9 , 12.1)**
**Burundi**	**69 (50 , 99)**	**3.3 (2.4 , 4.7)**	**85 (62 , 118)**	**2.3 (1.7 , 3.2)**	**-29.5 (-59.3 , 3.5)**
**Comoros**	**5 (3 , 7)**	**2.5 (1.5 , 3.4)**	**10 (7 , 14)**	**2.3 (1.6 , 3.1)**	**-6.6 (-28.8 , 36.3)**
**Djibouti**	**3 (2 , 4)**	**2.8 (2.1 , 3.6)**	**14 (10 , 19)**	**3.1 (2.3 , 4.1)**	**9.3 (-15.3 , 44.3)**
**Eritrea**	**20 (14 , 29)**	**2.6 (1.8 , 4)**	**60 (45 , 80)**	**2.9 (2.1 , 3.8)**	**11.1 (-22.1 , 57.2)**
**Ethiopia**	**462 (292 , 685)**	**2.8 (1.7 , 4)**	**853 (574 , 1111)**	**2.5 (1.7 , 3.3)**	**-9.8 (-35.9 , 20.5)**
**Kenya**	**83 (58 , 108)**	**1.2 (0.8 , 1.6)**	**289 (238 , 351)**	**1.7 (1.4 , 2)**	**37 (-5.6 , 82.5)**
**Madagascar**	**122**	**2.8**	**181**	**2.1**	**-23**

	(86 , 227)	(2 , 5.2)	(137 , 240)	(1.7 , 2.8)	(-58 , 16)
**Malawi**	264<br>(222 , 310)	8.3<br>(7 , 9.6)	490<br>(288 , 645)	8<br>(4.8 , 10.4)	-3.8<br>(-45.7 , 30.3)
**Mozambique**	154<br>(96 , 289)	3.2<br>(2 , 5.9)	269<br>(206 , 339)	3<br>(2.3 , 3.8)	-5.9<br>(-53.1 , 48.1)
**Rwanda**	83<br>(66 , 104)	3.4<br>(2.7 , 4.2)	123<br>(97 , 154)	2.5<br>(2 , 3.1)	-24.6<br>(-44.9 , 2.1)
**Somalia**	52<br>(34 , 79)	2.6<br>(1.7 , 4.1)	121<br>(71 , 207)	2.3<br>(1.4 , 3.9)	-11.5<br>(-35.7 , 17.4)
**South Sudan**	60<br>(43 , 84)	2.9<br>(2.1 , 4.1)	82<br>(55 , 124)	2.7<br>(1.8 , 4)	-7.5<br>(-32 , 24.5)
**United Republic of Tanzania**	274<br>(209 , 370)	3<br>(2.3 , 4)	562<br>(437 , 735)	2.7<br>(2.1 , 3.5)	-9.5<br>(-39.4 , 18.3)
**Uganda**	138<br>(114 , 163)	2.5<br>(2.1 , 3)	319<br>(246 , 391)	2.8<br>(2.2 , 3.4)	10<br>(-17.8 , 46.3)
**Zambia**	79<br>(64 , 96)	3.4<br>(2.8 , 4.1)	167<br>(127 , 217)	3.1<br>(2.4 , 4)	-9<br>(-30.1 , 20.1)
**Central Sub-Saharan Africa**	733<br>(404 , 1077)	4.4<br>(2.3 , 6.6)	1375<br>(862 , 2026)	3.3<br>(2.1 , 4.9)	-23.1<br>(-40.1 , 5.8)
**Angola**	107<br>(64 , 202)	3.6<br>(2 , 7)	279<br>(191 , 491)	3.4<br>(2.3 , 6)	-6.2<br>(-28.3 , 35.2)
**Central African Republic**	33<br>(23 , 45)	3.5<br>(2.3 , 4.6)	47<br>(32 , 65)	2.8<br>(1.9 , 3.7)	-20.8<br>(-38.8 , 2.4)
**Congo**	39<br>(29 , 54)	4.6<br>(3.2 , 6.5)	80<br>(58 , 120)	4.1<br>(3 , 6.1)	-11.5<br>(-33.4 , 17.6)
**Democratic Republic of the Congo**	524<br>(258 , 787)	4.6<br>(2.2 , 7)	913<br>(523 , 1313)	3.3<br>(1.8 , 4.7)	-29.2<br>(-47.2 , -0.1)
**Equatorial Guinea**	5<br>(3 , 7)	3<br>(1.8 , 4.1)	14<br>(9 , 19)	3.8<br>(2.6 , 5.2)	27.4<br>(-12.2 , 106)
**Gabon**	25<br>(15 , 44)	5<br>(3.1 , 9)	42<br>(28 , 75)	4.8<br>(3.3 , 8.7)	-4.3<br>(-32.5 , 36.1)
