# Supplementary material for: Global, regional and national burden of bladder cancer and its attributable risk factors in 204 countries and territories, 1990–2019: a systematic analysis for the Global Burden of Disease study 2019
Source: BMJ Glob Health. 2021 Nov 29;6(11):e004128. doi: 10.1136/bmjgh-2020-004128 (PMC8634015; doi:10.1136/bmjgh-2020-004128)
Supplement: Supplementary data [file bmjgh-2020-004128supp005.pdf]

Appendix Table 5: DALYs of bladder cancer and percentage change in age-standardised rates by location, 1990–2019					
	1990		2019		Percentage change in age-standardized rates between 1990 and 2019
	Counts (95% UI)	Rate (95% UI)	Counts (95% UI)	Rate (95% UI)	
Global	2567058 (2429467 , 2691105)	66.6 (63 , 69.7)	4392583 (4090438 , 4702733)	54.2 (50.4 , 58)	-18.6 (-24.3 , -11.2)
High-income North America	255118 (244625 , 263663)	72 (69.3 , 74.4)	433838 (405142 , 458840)	68.5 (64.3 , 72.2)	-4.9 (-8.8 , -1)
Canada	32012 (30494 , 33468)	98.4 (93.8 , 102.9)	49537 (44459 , 54895)	71.1 (64 , 78.5)	-27.8 (-34.1 , -20.6)
Greenland	37 (32 , 41)	116.4 (103.9 , 130.9)	57 (46 , 70)	85.4 (69.5 , 105.4)	-26.7 (-41.7 , -8.9)
United States of America	223064 (213715 , 230600)	69.3 (66.6 , 71.6)	384237 (359035 , 406940)	68.1 (64 , 72.1)	-1.7 (-5.8 , 2.5)
Australasia	17586 (16714 , 18381)	74.2 (70.5 , 77.5)	25849 (23069 , 28721)	50.8 (45.6 , 56.4)	-31.6 (-37.7 , -25)
Australia	14584 (13866 , 15277)	74 (70.2 , 77.4)	21680 (19143 , 24469)	50.5 (44.8 , 56.7)	-31.8 (-38.6 , -24.1)
New Zealand	3002 (2787 , 3212)	75.6 (70.2 , 80.8)	4169 (3690 , 4648)	52.6 (46.7 , 58.4)	-30.4 (-38.2 , -22.2)
High-income Asia Pacific	95954 (91672 , 99795)	48.9 (46.5 , 50.9)	185460 (162084 , 202861)	38.7 (34.7 , 42.3)	-20.8 (-26.3 , -16.1)
Brunei Darussalam	72 (60 , 93)	86 (71.2 , 111.8)	160 (137 , 188)	67.3 (57.9 , 78.6)	-21.8 (-42.5 , 0.8)
Japan	80136 (76244 , 83634)	48 (45.5 , 50.1)	148852 (128232 , 164037)	39.4 (34.9 , 43.1)	-18 (-24.2 , -12.8)
Singapore	1061 (972 , 1148)	50.4 (46.2 , 54.7)	1989 (1691 , 2323)	26.2 (22.3 , 30.7)	-48.1 (-56.4 , -39)
Republic of Korea	14685 (13804 , 15812)	53 (49.6 , 58)	34458 (30427 , 38806)	38.9 (34.3 , 43.7)	-26.6 (-35.2 , -16.2)
Western Europe	689660 (666293 , 710902)	118 (114 , 121.6)	805534 (736598 , 871055)	86.8 (80.2 , 93.9)	-26.4 (-30.7 , -21.9)
Andorra	51 (38 , 69)	94.5 (71.3 , 126.7)	105 (80 , 133)	74.5 (56.7 , 94.8)	-21.2 (-45.6 , 13.5)
Austria	10601 (10000 , 11182)	88 (83.2 , 92.9)	11619 (10477 , 13021)	63.8 (57.5 , 71.3)	-27.5 (-34.1 , -20)
Belgium	19757 (18746 , 20806)	127.1 (120.8 , 133.9)	20682 (18422 , 23139)	88.9 (79 , 99.2)	-30.1 (-37.2 , -22.1)
Cyprus	702	88.2	1691	85.7	-2.8

	(591 , 808)	(75.8 , 100.3)	(1457 , 1953)	(73.8 , 99)	(-20.8 , 20.1)
**Denmark**	**9357**<br>**(8850 , 9813)**	**115.6**<br>**(109.2 , 121.3)**	**12310**<br>**(10814 , 13891)**	**105**<br>**(92.6 , 117.9)**	**-9.2**<br>**(-19.7 , 2.8)**
**Finland**	**5075**<br>**(4819 , 5322)**	**70.6**<br>**(67.1 , 74.1)**	**5805**<br>**(5095 , 6583)**	**46**<br>**(40.5 , 51.9)**	**-34.9**<br>**(-42.7 , -26)**
**France**	**98463**<br>**(93331 , 103397)**	**119.7**<br>**(113.7 , 125.5)**	**124735**<br>**(110193 , 141735)**	**91.5**<br>**(81.1 , 103.8)**	**-23.6**<br>**(-31.4 , -13.7)**
**Germany**	**132536**<br>**(125838 , 138742)**	**103.2**<br>**(98.3 , 107.6)**	**139986**<br>**(125539 , 154722)**	**71.9**<br>**(64.6 , 79.2)**	**-30.4**<br>**(-37.4 , -23.2)**
**Greece**	**22148**<br>**(20863 , 23432)**	**143**<br>**(134.9 , 151.3)**	**27755**<br>**(25169 , 30595)**	**116.1**<br>**(105.5 , 128)**	**-18.8**<br>**(-25.8 , -10.8)**
**Iceland**	**267**<br>**(241 , 293)**	**92.9**<br>**(84 , 101.9)**	**363**<br>**(313 , 416)**	**64.6**<br>**(55.8 , 74.1)**	**-30.4**<br>**(-40.8 , -18.2)**
**Ireland**	**3296**<br>**(3087 , 3510)**	**79.2**<br>**(74.4 , 84.3)**	**4907**<br>**(4179 , 5698)**	**64.7**<br>**(55.2 , 74.9)**	**-18.3**<br>**(-31.5 , -4.7)**
**Israel**	**4140**<br>**(3832 , 4526)**	**85.1**<br>**(78.8 , 92.8)**	**9117**<br>**(8094 , 10179)**	**77.7**<br>**(69.3 , 86.7)**	**-8.7**<br>**(-18.1 , 2.6)**
**Italy**	**127921**<br>**(123394 , 132893)**	**141.4**<br>**(136.4 , 147)**	**140259**<br>**(126586 , 154546)**	**94.9**<br>**(87.1 , 104.6)**	**-32.9**<br>**(-36.9 , -28.3)**
**Luxembourg**	**608**<br>**(556 , 662)**	**110.6**<br>**(101.7 , 119.9)**	**790**<br>**(666 , 932)**	**78.2**<br>**(65.9 , 92.5)**	**-29.3**<br>**(-40.1 , -17.3)**
**Malta**	**486**<br>**(436 , 532)**	**113.3**<br>**(102.2 , 124)**	**710**<br>**(596 , 837)**	**75.9**<br>**(63.8 , 88.8)**	**-33**<br>**(-44.2 , -20.2)**
**Monaco**	**103**<br>**(81 , 129)**	**146.7**<br>**(115.4 , 183.5)**	**167**<br>**(123 , 298)**	**174.3**<br>**(126.7 , 311.6)**	**18.9**<br>**(-16.6 , 99.1)**
**Netherlands**	**22777**<br>**(21671 , 23877)**	**113.7**<br>**(108.4 , 119.2)**	**33610**<br>**(29943 , 37588)**	**97.8**<br>**(87.4 , 109.2)**	**-14**<br>**(-22.5 , -4.2)**
**Norway**	**7263**<br>**(6942 , 7581)**	**105**<br>**(100.5 , 109.4)**	**7184**<br>**(6445 , 7982)**	**73.3**<br>**(66 , 81.4)**	**-30.2**<br>**(-36.5 , -23.3)**
**Portugal**	**12827**<br>**(12147 , 13498)**	**91.8**<br>**(87 , 96.4)**	**19974**<br>**(17952 , 22142)**	**82.9**<br>**(74.6 , 92.1)**	**-9.8**<br>**(-19.2 , 0.1)**
**San Marino**	**51**<br>**(43 , 61)**	**151.1**<br>**(127.5 , 179.3)**	**94**<br>**(64 , 132)**	**141.8**<br>**(94.7 , 202.9)**	**-6.1**<br>**(-38.9 , 38.1)**
**Spain**	**79610**<br>**(75178 , 83580)**	**144**<br>**(136.2 , 151)**	**106160**<br>**(95077 , 119590)**	**109.6**<br>**(98 , 123.3)**	**-23.9**<br>**(-31 , -14.1)**
**Sweden**	**11258**<br>**(10608 , 11886)**	**73.6**<br>**(69.6 , 77.7)**	**15332**<br>**(13911 , 16768)**	**70.6**<br>**(64.6 , 77)**	**-4**<br>**(-11.5 , 3.9)**
**Switzerland**	**5703**<br>**(5338 , 6082)**	**54.2**<br>**(50.8 , 57.8)**	**11007**<br>**(9797 , 12398)**	**62.1**<br>**(55.6 , 70)**	**14.6**<br>**(2 , 30.5)**

**United Kingdom**	**114088 (110188 , 116973)**	**124.7 (120.6 , 127.9)**	**110467 (102544 , 116768)**	**85.7 (80.2 , 90.4)**	**-31.2 (-34.5 , -28.1)**
**Southern Latin America**	**41113 (39423 , 42817)**	**89.1 (85.4 , 92.8)**	**56963 (52392 , 61511)**	**68 (62.6 , 73.5)**	**-23.7 (-29.7 , -17.7)**
**Argentina**	**30958 (29491 , 32553)**	**95.6 (91 , 100.5)**	**39122 (35795 , 42561)**	**72.3 (66.3 , 78.6)**	**-24.3 (-31.2 , -16.8)**
**Chile**	**5474 (5101 , 5838)**	**56 (52.2 , 59.7)**	**12717 (11287 , 14083)**	**52.7 (46.8 , 58.4)**	**-5.9 (-17.7 , 6.3)**
**Uruguay**	**4680 (4388 , 4978)**	**118.7 (111.3 , 126.3)**	**5121 (4584 , 5680)**	**94.7 (84.9 , 105.3)**	**-20.2 (-29.4 , -10.2)**
**Eastern Europe**	**197055 (185829 , 211715)**	**69.1 (65.1 , 74.2)**	**231527 (203468 , 260692)**	**67.1 (59.1 , 75.5)**	**-2.9 (-13.9 , 10.2)**
**Belarus**	**8992 (8253 , 9803)**	**68.5 (63 , 74.9)**	**8660 (6682 , 11244)**	**54.1 (41.8 , 70.6)**	**-21 (-38.8 , 3.3)**
**Estonia**	**1637 (1514 , 1766)**	**79.5 (73.5 , 85.7)**	**1977 (1534 , 2500)**	**73.7 (56.7 , 93.5)**	**-7.3 (-28.8 , 20.5)**
**Latvia**	**2939 (2728 , 3175)**	**82.1 (76.2 , 88.5)**	**3726 (2954 , 4657)**	**94.1 (74.4 , 117.7)**	**14.6 (-10 , 45)**
**Lithuania**	**3991 (3757 , 4272)**	**88.8 (83.3 , 95.1)**	**4591 (3615 , 5708)**	**79.5 (62.2 , 99.7)**	**-10.4 (-30.1 , 12.4)**
**Republic of Moldova**	**3255 (2989 , 3566)**	**72 (66.1 , 78.8)**	**3924 (3291 , 4720)**	**67.4 (56.8 , 81)**	**-6.4 (-21.6 , 10.2)**
**Russian Federation**	**130930 (126618 , 136148)**	**71 (68.6 , 73.8)**	**150967 (127550 , 175536)**	**64.1 (54.2 , 74.5)**	**-9.6 (-22.6 , 4.2)**
**Ukraine**	**45309 (37852 , 55541)**	**61.9 (51.9 , 75.5)**	**57683 (47051 , 69365)**	**76.6 (62.6 , 92.4)**	**23.7 (-9.3 , 64)**
**Central Europe**	**148764 (144434 , 152664)**	**99.7 (96.8 , 102.3)**	**230525 (201856 , 262129)**	**107.4 (94 , 122.1)**	**7.7 (-5.8 , 22.1)**
**Albania**	**485 (449 , 525)**	**23.6 (21.7 , 25.7)**	**879 (662 , 1154)**	**20.7 (15.6 , 27.1)**	**-12.5 (-34.9 , 14.3)**
**Bosnia and Herzegovina**	**2772 (2566 , 2973)**	**68.1 (63 , 72.9)**	**5767 (4492 , 7216)**	**94.5 (73.8 , 118)**	**38.8 (7.4 , 75)**
**Bulgaria**	**9912 (9234 , 10631)**	**77.9 (72.6 , 83.4)**	**14805 (11510 , 18500)**	**105 (81.3 , 131.2)**	**34.8 (4.6 , 69.6)**
**Croatia**	**6022 (5576 , 6486)**	**94.6 (87.7 , 101.7)**	**8446 (6643 , 10527)**	**94.4 (74.2 , 118.4)**	**-0.3 (-22.1 , 28)**
**Czechia**	**15239 (14409 , 16003)**	**109.6 (103.7 , 114.9)**	**18223 (14535 , 22188)**	**85.2 (67.6 , 104.2)**	**-22.2 (-38.9 , -3.8)**
**Hungary**	**16260**	**110.1**	**20702**	**108.7**	**-1.3**

	(15492 , 17067)	(104.8 , 115.4)	(16865 , 25169)	(89 , 132.4)	(-20.4 , 19.5)
Montenegro	528<br>(442 , 615)	84.7<br>(70.5 , 98.5)	900<br>(736 , 1098)	90.4<br>(74.2 , 109.6)	6.7<br>(-18.2 , 42.1)
North Macedonia	1876<br>(1656 , 2101)	99.6<br>(87.8 , 111.5)	3860<br>(3033 , 4906)	118.4<br>(93.9 , 149.4)	18.8<br>(-8.2 , 51.6)
Poland	51367<br>(49694 , 52843)	116.2<br>(112.3 , 119.5)	89631<br>(74506 , 108322)	127.6<br>(105.8 , 154.4)	9.7<br>(-9.3 , 32.8)
Romania	24539<br>(23326 , 25705)	85.3<br>(81.3 , 89.3)	37281<br>(30289 , 46138)	102.5<br>(83.4 , 127.3)	20.1<br>(-2.1 , 48.8)
Serbia	11873<br>(9884 , 13751)	104.1<br>(86.8 , 119.6)	18790<br>(14811 , 23532)	116.7<br>(92.2 , 146.3)	12.1<br>(-14.9 , 48.3)
Slovakia	5773<br>(5389 , 6162)	95.6<br>(89.3 , 102.1)	7885<br>(6266 , 9985)	84.3<br>(66.7 , 106.4)	-11.8<br>(-30.6 , 12.2)
Slovenia	2118<br>(1630 , 2707)	86.4<br>(66.5 , 110.6)	3356<br>(2562 , 4288)	76.3<br>(57.8 , 98.6)	-11.7<br>(-38.7 , 25.6)
Central Asia	23643<br>(20738 , 27078)	49.5<br>(43.3 , 56.9)	39201<br>(35199 , 43678)	53.6<br>(48.2 , 59.4)	8.2<br>(-5.6 , 29)
Armenia	2979<br>(2535 , 3373)	110.9<br>(94.2 , 124.7)	4351<br>(3581 , 5213)	104.3<br>(85.8 , 124.2)	-6<br>(-26.6 , 19.6)
Azerbaijan	2889<br>(2458 , 3358)	54.2<br>(46.6 , 62.3)	5744<br>(4656 , 7086)	58.6<br>(48.2 , 71.3)	8.2<br>(-15.4 , 37.8)
Georgia	4459<br>(3701 , 5209)	70.2<br>(58.5 , 81.4)	5870<br>(4899 , 6928)	100.4<br>(83.4 , 118.8)	43.1<br>(14.4 , 78.1)
Kazakhstan	6945<br>(5616 , 8960)	53.8<br>(43.8 , 69.3)	8721<br>(7368 , 10360)	49.9<br>(42.1 , 59.1)	-7.2<br>(-28.8 , 21)
Kyrgyzstan	1200<br>(1069 , 1343)	39.2<br>(34.9 , 43.9)	1463<br>(1228 , 1730)	31.7<br>(26.6 , 37.1)	-19.1<br>(-33.5 , -0.6)
Mongolia	560<br>(460 , 667)	50.3<br>(41.8 , 59.4)	637<br>(491 , 817)	27.5<br>(21.5 , 34.4)	-45.4<br>(-59 , -26.8)
Tajikistan	766<br>(575 , 941)	27.1<br>(19.9 , 33.9)	1548<br>(1254 , 1925)	32.9<br>(27.1 , 40.1)	21.3<br>(-8.9 , 73.2)
Turkmenistan	390<br>(340 , 441)	20.3<br>(17.6 , 22.9)	1333<br>(1037 , 1720)	32.2<br>(24.8 , 41.2)	59.1<br>(22.7 , 104.4)
Uzbekistan	3456<br>(2627 , 4774)	29.4<br>(21.9 , 41.5)	9533<br>(7780 , 11488)	44.4<br>(36.8 , 52.8)	50.9<br>(6.3 , 113.9)
Central Latin America	26530<br>(25466 , 27571)	32.4<br>(30.9 , 33.8)	67318<br>(57815 , 78818)	28.8<br>(24.7 , 33.7)	-11.1<br>(-24.1 , 4)
Colombia	6883<br>(6462 , 7353)	40.1<br>(37.5 , 43.1)	13398<br>(10250 , 17367)	25.4<br>(19.4 , 32.8)	-36.8<br>(-51.9 , -18.5)

**Costa Rica**	**703 (645 , 761)**	**41.2 (37.7 , 44.7)**	**1900 (1454 , 2440)**	**37.4 (28.6 , 48.1)**	**-9.1 (-31 , 16.6)**
**El Salvador**	**554 (514 , 599)**	**18.9 (17.5 , 20.5)**	**1243 (945 , 1603)**	**20.8 (15.8 , 26.9)**	**9.9 (-18.5 , 43.4)**
**Guatemala**	**791 (696 , 900)**	**22.9 (20.1 , 26)**	**2305 (1790 , 2931)**	**20.8 (16.3 , 26.3)**	**-9.4 (-31.2 , 20.2)**
**Honduras**	**431 (350 , 511)**	**21.3 (17.3 , 25.2)**	**1717 (1304 , 2470)**	**29.8 (22.7 , 42.9)**	**39.5 (3.6 , 99.3)**
**Mexico**	**12929 (12414 , 13413)**	**30.8 (29.4 , 32)**	**33727 (28734 , 39187)**	**29.2 (24.9 , 33.8)**	**-5.4 (-18.7 , 10.3)**
**Nicaragua**	**313 (271 , 351)**	**20.6 (17.6 , 23.2)**	**922 (759 , 1113)**	**21.9 (18.1 , 26.1)**	**6.3 (-15 , 37)**
**Panama**	**445 (404 , 485)**	**30.2 (27.3 , 33.1)**	**998 (755 , 1279)**	**24.1 (18.2 , 30.9)**	**-20.2 (-40.4 , 4.1)**
**Venezuela (Bolivarian Republic of)**	**3480 (3262 , 3735)**	**36.6 (34.2 , 39.4)**	**11109 (8293 , 14353)**	**38.6 (29.1 , 49.8)**	**5.4 (-21.5 , 38.3)**
**Andean Latin America**	**6730 (6035 , 7524)**	**33.5 (30.1 , 37.4)**	**16303 (13241 , 19799)**	**29.5 (24 , 35.8)**	**-12 (-29.6 , 9.3)**
**Bolivia (Plurinational State of)**	**1512 (1205 , 1844)**	**48.4 (38.8 , 59.2)**	**4110 (3182 , 5198)**	**48.3 (37.7 , 60.7)**	**-0.1 (-24.4 , 33.7)**
**Ecuador**	**1537 (1429 , 1655)**	**29.4 (27.2 , 31.8)**	**4372 (3415 , 5549)**	**29.7 (23.4 , 37.6)**	**0.9 (-21 , 27.6)**
**Peru**	**3681 (3148 , 4318)**	**31.4 (26.9 , 36.8)**	**7822 (5853 , 10398)**	**24.5 (18.4 , 32.5)**	**-22 (-43.1 , 7.9)**
**Caribbean**	**14302 (13359 , 15206)**	**55.5 (51.8 , 59)**	**28597 (24789 , 33112)**	**55.3 (47.9 , 64)**	**-0.5 (-14.3 , 15.5)**
**Antigua and Barbuda**	**26 (23 , 28)**	**47.8 (43 , 52.7)**	**51 (42 , 60)**	**52 (43.4 , 61.2)**	**8.7 (-10.8 , 30.9)**
**Barbados**	**134 (122 , 147)**	**45.3 (41.4 , 49.3)**	**247 (201 , 295)**	**49.9 (40.7 , 59.6)**	**10.3 (-12.1 , 33.5)**
**Belize**	**30 (26 , 32)**	**32.1 (28.6 , 35.4)**	**114 (98 , 132)**	**41.9 (35.9 , 48.5)**	**30.6 (8 , 56.2)**
**Bermuda**	**77 (70 , 85)**	**125.1 (112.7 , 138.2)**	**112 (92 , 135)**	**84.6 (70 , 102.1)**	**-32.4 (-45 , -16.1)**
**Bahamas**	**58 (53 , 65)**	**38.2 (34.3 , 42.4)**	**139 (112 , 171)**	**36.2 (29.3 , 44)**	**-5.1 (-24.4 , 19)**
**Cuba**	**7299**	**70.8**	**14577**	**76.5**	**7.9**

	(6888 , 7706)	(66.8 , 74.7)	(11626 , 18061)	(60.9 , 94.7)	(-13 , 34.5)
**Dominica**	**47 (41 , 53)**	**64.5 (56.9 , 72.5)**	**64 (53 , 80)**	**71.2 (58.8 , 88)**	**10.4 (-11.4 , 38)**
**Dominican Republic**	**757 (657 , 865)**	**20.7 (17.8 , 23.6)**	**2532 (1839 , 3447)**	**27.6 (20.2 , 37.5)**	**33.3 (-5.9 , 88.3)**
**Grenada**	**39 (34 , 44)**	**53.6 (47 , 60.9)**	**67 (59 , 76)**	**61 (53.5 , 69.1)**	**13.7 (-5.3 , 34.9)**
**Guyana**	**145 (123 , 167)**	**38.7 (33.2 , 44.5)**	**242 (186 , 312)**	**38.8 (30 , 49.5)**	**0.3 (-24.9 , 32.7)**
**Haiti**	**2066 (1384 , 2757)**	**64.5 (43.4 , 85.2)**	**3821 (2354 , 5625)**	**56.7 (35.3 , 82.8)**	**-12.2 (-34.5 , 19.6)**
**Jamaica**	**891 (807 , 980)**	**49.7 (45.2 , 54.6)**	**1384 (1075 , 1739)**	**46.5 (36 , 58.6)**	**-6.5 (-27.8 , 18.3)**
**Puerto Rico**	**1661 (1531 , 1787)**	**45.9 (42.3 , 49.4)**	**3076 (2392 , 3959)**	**42.8 (33 , 55.2)**	**-6.9 (-29.3 , 21.1)**
**Saint Kitts and Nevis**	**29 (27 , 33)**	**78.2 (70.8 , 86.4)**	**43 (35 , 51)**	**67.5 (56.9 , 79.7)**	**-13.7 (-29.7 , 5.2)**
**Saint Lucia**	**63 (57 , 68)**	**72.7 (66.1 , 78.7)**	**137 (114 , 162)**	**64.1 (53.6 , 76.1)**	**-11.8 (-27.5 , 6.1)**
**Saint Vincent and the Grenadines**	**34 (31 , 38)**	**47.2 (42.6 , 52.1)**	**70 (61 , 82)**	**52.7 (45.8 , 61.1)**	**11.8 (-5.5 , 32.2)**
**Suriname**	**84 (76 , 92)**	**32.9 (29.9 , 36.4)**	**222 (183 , 267)**	**37.3 (30.8 , 44.8)**	**13.4 (-8.1 , 38.8)**
**Trinidad and Tobago**	**360 (337 , 389)**	**42.6 (39.8 , 45.9)**	**650 (479 , 865)**	**35.1 (26 , 46.5)**	**-17.7 (-40.2 , 9.3)**
**United States Virgin Islands**	**27 (21 , 32)**	**32.3 (26 , 38.7)**	**79 (66 , 96)**	**42 (34.9 , 50.7)**	**30.1 (-1.7 , 75.3)**
**Tropical Latin America**	**47982 (46040 , 49696)**	**54.2 (51.6 , 56.3)**	**109218 (101213 , 116267)**	**45.6 (42.2 , 48.6)**	**-15.9 (-21.4 , -10.3)**
**Brazil**	**47485 (45584 , 49182)**	**55 (52.4 , 57.1)**	**107757 (99750 , 114563)**	**46 (42.5 , 49)**	**-16.3 (-21.9 , -10.8)**
**Paraguay**	**496 (426 , 574)**	**22.7 (19.4 , 26.2)**	**1461 (1098 , 1895)**	**26.8 (20.3 , 34.7)**	**18.2 (-15.4 , 65.6)**
**East Asia**	**424273 (371323 , 477145)**	**49.8 (43.7 , 55.8)**	**856972 (729753 , 1009067)**	**42.4 (36.3 , 49.6)**	**-14.7 (-30 , 5.4)**
**China**	**405561 (353228 , 459123)**	**49.4 (43.2 , 55.6)**	**816119 (691348 , 967379)**	**41.9 (35.6 , 49.3)**	**-15.2 (-31.2 , 5.6)**
**Democratic People's Republic**	**7070**	**43.7**	**12525**	**38.9**	**-11**

of Korea	(5175 , 9240)	(32.5 , 56.4)	(10308 , 15175)	(32.3 , 46.8)	(-33.7 , 23.5)
Taiwan (Province of China)	11642 (11140 , 12204)	74.4 (71.1 , 78)	28328 (21748 , 36869)	71.7 (55.1 , 93.5)	-3.6 (-25.3 , 26)
Southeast Asia	90055 (79438 , 99699)	36.1 (31.9 , 39.9)	194862 (169280 , 229270)	33.2 (28.9 , 38.8)	-8.1 (-21.7 , 8.9)
Cambodia	1801 (1364 , 2362)	40.9 (30.9 , 53.1)	4419 (3336 , 5524)	38.4 (29.1 , 47.7)	-6.1 (-30.2 , 22.3)
Indonesia	30236 (24440 , 36387)	31.5 (25.5 , 37.8)	70994 (53035 , 101431)	34.6 (26.1 , 48.9)	9.9 (-16.6 , 39.3)
Lao People's Democratic Republic	971 (676 , 1301)	46.6 (32.3 , 62.1)	1543 (1104 , 1977)	36.4 (26.4 , 45.4)	-22 (-42.6 , 6.8)
Malaysia	4910 (4073 , 5660)	56.6 (46.2 , 65.5)	13939 (10799 , 17612)	55.4 (43.4 , 69.4)	-2 (-27.8 , 39)
Maldives	36 (29 , 47)	48 (38.1 , 61.2)	91 (74 , 110)	33.3 (26.7 , 40.1)	-30.7 (-50.8 , -4.6)
Mauritius	505 (465 , 543)	68.7 (63.3 , 73.7)	725 (578 , 906)	41.5 (33.5 , 51.6)	-39.6 (-52.3 , -23.6)
Myanmar	10301 (7152 , 13979)	44.6 (31 , 59.5)	15602 (12531 , 19486)	34.5 (28 , 42.6)	-22.7 (-42.2 , 7.2)
Philippines	8291 (7163 , 9392)	27.2 (23.5 , 30.9)	18504 (15168 , 22342)	23.5 (19.5 , 28.1)	-13.8 (-32.4 , 10.1)
Sri Lanka	2193 (1941 , 2461)	21.5 (19 , 24.3)	6284 (4663 , 8355)	25.1 (18.8 , 32.9)	16.7 (-14.8 , 57.4)
Seychelles	53 (46 , 62)	94.7 (82 , 109.1)	94 (80 , 109)	88.4 (76.4 , 102.8)	-6.7 (-22.6 , 10.6)
Thailand	18898 (16437 , 21589)	54.9 (47.8 , 62.7)	30182 (22571 , 39287)	30.1 (22.6 , 39)	-45.2 (-60.4 , -26)
Timor-Leste	83 (57 , 120)	30.2 (20.6 , 43)	241 (171 , 328)	30.7 (22.1 , 41.5)	1.6 (-27.1 , 50.5)
Viet Nam	11656 (9281 , 14034)	29.4 (23.6 , 35.3)	31991 (24834 , 39949)	35.2 (27.6 , 43.3)	19.5 (-10.5 , 62.5)
Oceania	979 (781 , 1233)	32.3 (25.7 , 40.3)	2817 (2177 , 3679)	38.5 (30 , 49.2)	19.5 (-3.2 , 47.7)
American Samoa	9 (7 , 10)	37.1 (32.4 , 41.9)	23 (20 , 28)	48.9 (41.1 , 57.7)	31.9 (5.8 , 62.4)
Cook Islands	9 (7 , 11)	69.4 (57.6 , 82.4)	15 (12 , 18)	61.5 (50.3 , 74.3)	-11.4 (-33.4 , 13.9)
Micronesia (Federated States	24	49.7	42	57.5	15.8

**of)**	**(19 , 30)**	**(39 , 61.8)**	**(29 , 57)**	**(42.4 , 75.3)**	**(-18.7 , 60.6)**
**Fiji**	**134 (108 , 167)**	**36.4 (29.8 , 44.3)**	**336 (264 , 427)**	**44.5 (35.2 , 55.4)**	**22.2 (-11 , 69.5)**
**Guam**	**28 (24 , 33)**	**36.1 (31.3 , 42)**	**72 (59 , 88)**	**38 (31.1 , 46.2)**	**5.3 (-16.4 , 32.5)**
**Kiribati**	**14 (11 , 17)**	**37.3 (31 , 44.7)**	**24 (18 , 30)**	**34.9 (28 , 42.3)**	**-6.5 (-27.8 , 19.2)**
**Marshall Islands**	**8 (7 , 11)**	**50.7 (39.4 , 63.8)**	**21 (15 , 28)**	**57.5 (44 , 74.3)**	**13.4 (-14 , 50.1)**
**Nauru**	**2 (2 , 3)**	**57.5 (43.4 , 73.5)**	**3 (2 , 4)**	**62.3 (47.5 , 79.7)**	**8.4 (-14.2 , 38.2)**
**Niue**	**1 (1 , 1)**	**42.1 (34.6 , 51.6)**	**1 (1 , 1)**	**45.5 (35.5 , 56.3)**	**8 (-17.2 , 37.7)**
**Northern Mariana Islands**	**5 (4 , 7)**	**29.4 (24.9 , 35.4)**	**28 (23 , 34)**	**54.5 (45.2 , 65)**	**85.7 (45.9 , 128.2)**
**Palau**	**2 (2 , 3)**	**21.6 (17.4 , 27.4)**	**5 (4 , 6)**	**21.8 (17.6 , 27.4)**	**1 (-26.4 , 34.2)**
**Papua New Guinea**	**535 (378 , 724)**	**28.1 (19.9 , 37.5)**	**1732 (1221 , 2447)**	**35.1 (24.8 , 48.3)**	**24.8 (-5.9 , 62.9)**
**Samoa**	**39 (32 , 48)**	**45.4 (37.4 , 55.4)**	**64 (51 , 82)**	**44.5 (35.9 , 56)**	**-2 (-24.9 , 29.7)**
**Solomon Islands**	**73 (49 , 108)**	**49.8 (34 , 71.7)**	**205 (138 , 288)**	**59.8 (43 , 80.9)**	**20 (-11.3 , 61.9)**
**Tokelau**	**1 (0 , 1)**	**38.9 (30.4 , 52.3)**	**1 (0 , 1)**	**40.7 (31.3 , 54.4)**	**4.6 (-22.6 , 41.4)**
**Tonga**	**17 (13 , 21)**	**30.3 (23.2 , 38.5)**	**29 (21 , 39)**	**36.8 (27.3 , 48.7)**	**21.3 (-7.1 , 59.2)**
**Tuvalu**	**3 (3 , 4)**	**45.9 (36.6 , 58.6)**	**5 (4 , 6)**	**46.9 (36.3 , 61.2)**	**2.2 (-24 , 38.4)**
**Vanuatu**	**22 (15 , 30)**	**33 (23.6 , 45.6)**	**79 (58 , 105)**	**44.7 (32.7 , 59.5)**	**35.4 (-1.9 , 86.3)**
**North Africa and Middle East**	**161349 (139841 , 182742)**	**90.9 (77.9 , 103.1)**	**378666 (314896 , 461347)**	**86.2 (72.3 , 103.6)**	**-5.2 (-25.2 , 28.7)**
**Afghanistan**	**6077 (3793 , 9060)**	**86.6 (53.7 , 126.7)**	**9146 (6286 , 12732)**	**74.6 (51.3 , 100.3)**	**-13.8 (-37.9 , 18.3)**
**Algeria**	**5491 (4288 , 6915)**	**50.9 (40.9 , 63.4)**	**12204 (9606 , 15230)**	**39.4 (31.3 , 49)**	**-22.5 (-45.3 , 4.9)**
**Bahrain**	**207 (171 , 250)**	**145 (120.9 , 172.7)**	**589 (443 , 768)**	**82.2 (63.7 , 103)**	**-43.3 (-58.3 , -22.7)**

**Egypt**	**65552 (59542 , 72491)**	**192.8 (176.1 , 211.9)**	**146006 (94856 , 213427)**	**201.8 (132.1 , 294.4)**	**4.6 (-32 , 57)**
**Iran (Islamic Republic of)**	**12576 (10087 , 14634)**	**49.8 (39.5 , 58.3)**	**35675 (33013 , 38856)**	**50.2 (46.2 , 54.7)**	**0.7 (-16.3 , 33.2)**
**Iraq**	**8189 (5921 , 10616)**	**107.5 (77.2 , 139.4)**	**28576 (21821 , 35369)**	**132.6 (103.1 , 161.3)**	**23.4 (-14.8 , 86.7)**
**Jordan**	**950 (769 , 1158)**	**76.4 (62.2 , 93.2)**	**4150 (3290 , 5242)**	**67.7 (54.1 , 84.7)**	**-11.3 (-34.9 , 25.1)**
**Kuwait**	**442 (387 , 499)**	**77.2 (66.5 , 87.5)**	**1487 (1185 , 1857)**	**66.1 (53.2 , 82.2)**	**-14.4 (-32.8 , 8.9)**
**Lebanon**	**4867 (3924 , 5980)**	**225.5 (184.8 , 274.4)**	**10052 (7740 , 13264)**	**192.7 (147.9 , 254.9)**	**-14.5 (-37.9 , 25.5)**
**Libya**	**1849 (1312 , 2432)**	**103.8 (73.7 , 137.9)**	**5047 (3708 , 6581)**	**104.5 (77.3 , 135.2)**	**0.6 (-34.2 , 67.8)**
**Morocco**	**5074 (3874 , 6230)**	**38.4 (28.9 , 46.7)**	**13633 (10170 , 17523)**	**45.6 (34.7 , 58.1)**	**18.6 (-15 , 63.9)**
**Palestine**	**638 (437 , 855)**	**77.2 (52.8 , 102.7)**	**1522 (1284 , 1825)**	**68.9 (58.1 , 82.7)**	**-10.7 (-37.3 , 41.1)**
**Oman**	**283 (185 , 384)**	**45.5 (30 , 60.9)**	**682 (534 , 866)**	**45.5 (37.8 , 54.4)**	**0.1 (-31 , 60.3)**
**Qatar**	**49 (37 , 63)**	**61.4 (45.3 , 79.3)**	**376 (264 , 521)**	**78.2 (58.2 , 102.6)**	**27.4 (-13.8 , 92.3)**
**Saudi Arabia**	**2051 (1354 , 2798)**	**37.1 (24.4 , 50.2)**	**5812 (4424 , 7718)**	**32.9 (26.2 , 41.7)**	**-11.3 (-40.6 , 55.5)**
**Sudan**	**6490 (3206 , 15184)**	**71.7 (34.7 , 168.7)**	**10810 (7066 , 19114)**	**61 (40.2 , 108.1)**	**-14.9 (-47.2 , 56.3)**
**Syrian Arab Republic**	**2015 (1514 , 2562)**	**38.5 (28.9 , 49.1)**	**4790 (3513 , 6460)**	**40.1 (29.7 , 53.1)**	**4.1 (-30.7 , 65.2)**
**Tunisia**	**3954 (3091 , 4907)**	**82.9 (65.3 , 102.3)**	**9648 (6896 , 13456)**	**78.5 (56.4 , 108.7)**	**-5.3 (-37.7 , 47.9)**
**Turkey**	**30937 (24466 , 38155)**	**87.3 (68.4 , 107.1)**	**65174 (51656 , 79851)**	**74.3 (59.2 , 90.8)**	**-14.9 (-36.4 , 20)**
**United Arab Emirates**	**678 (339 , 1600)**	**149.3 (69.6 , 378.7)**	**5505 (2983 , 9346)**	**115 (61.3 , 197.5)**	**-23 (-57.9 , 42.1)**
**Yemen**	**2868 (1509 , 4563)**	**59.6 (31 , 94.8)**	**7400 (5264 , 10052)**	**57.8 (41.3 , 77.8)**	**-3.1 (-35.2 , 61.4)**
**South Asia**	**196582 (170590 , 223318)**	**37.4 (32 , 42.4)**	**487701 (432733 , 550474)**	**35.3 (31.1 , 39.7)**	**-5.8 (-20.9 , 14.6)**
**Bangladesh**	**12254**	**26.5**	**28324**	**22.2**	**-16.2**

	(9495 , 15344)	(20.4 , 32.9)	(19773 , 38143)	(15.6 , 29.8)	(-41.6 , 12.3)
**Bhutan**	**57**<br>**(36 , 82)**	**23.8**<br>**(14.9 , 33.4)**	**147**<br>**(102 , 194)**	**27.3**<br>**(19.1 , 35.8)**	**15.1**<br>**(-17.7 , 61.1)**
**India**	**120905**<br>**(104241 , 139333)**	**28.4**<br>**(24.3 , 32.8)**	**313586**<br>**(268642 , 366203)**	**28.2**<br>**(24.2 , 32.9)**	**-0.9**<br>**(-17.9 , 21.4)**
**Nepal**	**2227**<br>**(1462 , 3073)**	**24.3**<br>**(15.6 , 33.2)**	**5708**<br>**(4042 , 7332)**	**26.8**<br>**(19.1 , 34.6)**	**10.6**<br>**(-17.9 , 49.3)**
**Pakistan**	**61139**<br>**(49708 , 73023)**	**109.5**<br>**(88.4 , 131.2)**	**139936**<br>**(110285 , 178027)**	**129.3**<br>**(102.2 , 164.5)**	**18.1**<br>**(-11.8 , 62.6)**
**Southern Sub-Saharan Africa**	**18346**<br>**(16393 , 20142)**	**64.6**<br>**(57.5 , 71.2)**	**34673**<br>**(30365 , 39313)**	**61.1**<br>**(53.8 , 68.9)**	**-5.5**<br>**(-19.9 , 12)**
**Botswana**	**339**<br>**(255 , 442)**	**58.6**<br>**(44.9 , 75.4)**	**865**<br>**(614 , 1145)**	**61.1**<br>**(44.9 , 79.3)**	**4.3**<br>**(-26.8 , 44.6)**
**Lesotho**	**392**<br>**(298 , 513)**	**39.7**<br>**(30.2 , 51.4)**	**702**<br>**(480 , 951)**	**54.9**<br>**(38.3 , 72.9)**	**38.3**<br>**(1 , 93.5)**
**Namibia**	**266**<br>**(211 , 326)**	**36.7**<br>**(29.4 , 44.8)**	**549**<br>**(423 , 702)**	**39.8**<br>**(31.2 , 50.2)**	**8.5**<br>**(-19.4 , 46.4)**
**South Africa**	**10579**<br>**(9263 , 11811)**	**48.1**<br>**(41.8 , 54)**	**19654**<br>**(17652 , 21639)**	**44.1**<br>**(39.7 , 48.4)**	**-8.4**<br>**(-18.7 , 5.6)**
**Eswatini**	**160**<br>**(124 , 211)**	**53.5**<br>**(42 , 70.2)**	**334**<br>**(243 , 449)**	**56.7**<br>**(42.3 , 74.4)**	**6.1**<br>**(-22.8 , 45.3)**
**Zimbabwe**	**6611**<br>**(5735 , 7559)**	**163.3**<br>**(141.9 , 185.7)**	**12569**<br>**(8118 , 16513)**	**179.1**<br>**(116.7 , 231.9)**	**9.6**<br>**(-29.3 , 48.7)**
**Western Sub-Saharan Africa**	**47888**<br>**(39916 , 57251)**	**55.4**<br>**(46.4 , 66.4)**	**89312**<br>**(61202 , 107240)**	**48.2**<br>**(33.4 , 57.2)**	**-13**<br>**(-45.6 , 12.3)**
**Benin**	**1512**<br>**(1192 , 2223)**	**76.7**<br>**(60.5 , 112.6)**	**2396**<br>**(1800 , 3102)**	**48.6**<br>**(36.7 , 61.4)**	**-36.6**<br>**(-64.2 , -10.9)**
**Burkina Faso**	**3156**<br>**(2181 , 5300)**	**74.6**<br>**(51.6 , 125.1)**	**4255**<br>**(2749 , 5839)**	**46.3**<br>**(30.7 , 62.7)**	**-37.9**<br>**(-72.8 , -8)**
**Cameroon**	**3497**<br>**(2831 , 4313)**	**80.8**<br>**(65.6 , 98.8)**	**7819**<br>**(4597 , 10862)**	**64.2**<br>**(38.4 , 87.3)**	**-20.6**<br>**(-48 , 8.8)**
**Cabo Verde**	**48**<br>**(42 , 54)**	**20.6**<br>**(18 , 23.2)**	**273**<br>**(228 , 324)**	**63.1**<br>**(53.2 , 74.5)**	**206.7**<br>**(152.4 , 272.8)**
**Chad**	**1820**<br>**(1357 , 2307)**	**64.9**<br>**(48.5 , 82.2)**	**3269**<br>**(2323 , 4312)**	**58.1**<br>**(41.7 , 76.3)**	**-10.4**<br>**(-30.8 , 16.3)**
**CÃ´te d'Ivoire**	**3457**<br>**(2571 , 4653)**	**88.6**<br>**(67.9 , 118.5)**	**6347**<br>**(4692 , 8357)**	**58.5**<br>**(44.5 , 75.2)**	**-34**<br>**(-57.8 , -8.2)**
**Gambia**	**113**<br>**(85 , 145)**	**33.1**<br>**(25.6 , 42.1)**	**367**<br>**(265 , 493)**	**39.5**<br>**(28.9 , 52.6)**	**19.2**<br>**(-19.6 , 74.7)**

**Ghana**	**5028 (3901 , 7709)**	**75.3 (59.6 , 117.6)**	**10775 (5662 , 14501)**	**60.2 (32.9 , 79.4)**	**-20.1 (-56.8 , 17.3)**
**Guinea**	**3610 (2997 , 4342)**	**111.2 (92.1 , 133.3)**	**6536 (4341 , 9183)**	**122 (81.7 , 169.5)**	**9.8 (-28.2 , 63.6)**
**Guinea-Bissau**	**382 (270 , 541)**	**93.7 (67.6 , 129.8)**	**493 (365 , 658)**	**65.6 (50 , 85.7)**	**-30 (-50.4 , -3.6)**
**Liberia**	**992 (671 , 1919)**	**90.2 (61.5 , 174.7)**	**1087 (755 , 1635)**	**52.1 (37.5 , 77.1)**	**-42.2 (-64.4 , -10.9)**
**Mali**	**8256 (6885 , 9688)**	**202.4 (170.3 , 235.8)**	**16402 (7230 , 22689)**	**197 (86.5 , 270.6)**	**-2.7 (-57.8 , 40.4)**
**Mauritania**	**834 (659 , 1062)**	**83.6 (66.1 , 106.6)**	**961 (656 , 1412)**	**47 (32.5 , 67.1)**	**-43.8 (-60.5 , -20.5)**
**Niger**	**1868 (1297 , 2429)**	**68 (47.2 , 87.4)**	**3394 (2092 , 4849)**	**44.5 (28.2 , 63.4)**	**-34.5 (-57.9 , -9.7)**
**Nigeria**	**8177 (5893 , 10823)**	**19.5 (14.2 , 25.6)**	**16934 (13192 , 22063)**	**21.4 (16.9 , 27.3)**	**9.3 (-25.3 , 65.9)**
**Sao Tome and Principe**	**48 (37 , 58)**	**75.1 (58.9 , 91.2)**	**108 (81 , 145)**	**100.5 (77 , 132.9)**	**33.7 (-0.6 , 81.1)**
**Senegal**	**2536 (1915 , 3156)**	**80 (61.4 , 99)**	**4200 (2643 , 5646)**	**56.3 (36 , 74.6)**	**-29.5 (-51.5 , -2.4)**
**Sierra Leone**	**1554 (1016 , 3051)**	**82.1 (53.8 , 161.4)**	**1768 (1125 , 2444)**	**48.6 (31.2 , 66.1)**	**-40.8 (-78.6 , -1.3)**
**Togo**	**999 (791 , 1365)**	**81.7 (65.5 , 111.4)**	**1924 (1112 , 2641)**	**51.2 (30.6 , 69.1)**	**-37.3 (-69.6 , -8.6)**
**Eastern Sub-Saharan Africa**	**45241 (35091 , 57788)**	**61.2 (47.6 , 78.2)**	**84041 (71428 , 99339)**	**53.5 (45.8 , 62.7)**	**-12.5 (-32.9 , 8.2)**
**Burundi**	**1622 (1168 , 2325)**	**69.4 (50.4 , 99.6)**	**2103 (1483 , 2945)**	**47.1 (33.9 , 65.1)**	**-32.1 (-61.4 , 3.1)**
**Comoros**	**109 (57 , 154)**	**49.8 (27.3 , 69.5)**	**218 (145 , 302)**	**45.8 (30.7 , 62.8)**	**-8.1 (-32.6 , 57.6)**
**Djibouti**	**76 (54 , 105)**	**56.1 (41.8 , 74.5)**	**344 (229 , 500)**	**60 (42.4 , 83.6)**	**6.9 (-20.9 , 46.5)**
**Eritrea**	**563 (397 , 801)**	**56.3 (39.5 , 81.8)**	**1558 (1151 , 2101)**	**59 (44.1 , 78.4)**	**4.9 (-25.6 , 47.3)**
**Ethiopia**	**11838 (7672 , 17801)**	**59.2 (37.4 , 87.5)**	**18468 (12518 , 24100)**	**47 (31.7 , 61.5)**	**-20.5 (-45.4 , 9.9)**
**Kenya**	**1882 (1304 , 2435)**	**23.5 (16.2 , 30.3)**	**6750 (5490 , 8281)**	**32 (26.3 , 38.8)**	**36.2 (-7.3 , 82.1)**
**Madagascar**	**2871**	**56.2**	**4635**	**43.1**	**-23.3**

	(2035 , 5352)	(39.7 , 105.8)	(3443 , 6175)	(32.7 , 57.1)	(-58.4 , 17.5)
**Malawi**	6488<br>(5406 , 7713)	169<br>(142.3 , 198.2)	11463<br>(6575 , 15537)	160.2<br>(93.7 , 213.5)	-5.2<br>(-48.3 , 32.6)
**Mozambique**	3623<br>(2235 , 6689)	62.6<br>(39 , 116.3)	6442<br>(4742 , 8342)	60.2<br>(45.7 , 76.6)	-3.8<br>(-53.6 , 56.7)
**Rwanda**	2035<br>(1604 , 2554)	70.4<br>(55.8 , 87.8)	2858<br>(2206 , 3692)	49.3<br>(38.7 , 62.1)	-30<br>(-49.3 , -1)
**Somalia**	1369<br>(897 , 2090)	54.8<br>(36.3 , 83.7)	3166<br>(1854 , 5439)	48<br>(28.3 , 81.7)	-12.3<br>(-37.6 , 18.7)
**South Sudan**	1353<br>(948 , 1917)	58.1<br>(41.3 , 81.9)	1876<br>(1200 , 2925)	51.7<br>(34 , 78.2)	-11<br>(-35.4 , 23.5)
**United Republic of Tanzania**	6357<br>(4746 , 8688)	59.4<br>(45 , 80.3)	12690<br>(9639 , 16954)	53.2<br>(40.9 , 70.1)	-10.5<br>(-40.9 , 18.7)
**Uganda**	3121<br>(2538 , 3718)	49.8<br>(40.9 , 58.8)	7318<br>(5543 , 9180)	54.1<br>(41.5 , 66.8)	8.7<br>(-20.7 , 47.3)
**Zambia**	1900<br>(1535 , 2332)	67.9<br>(55 , 83)	4084<br>(3024 , 5498)	61.9<br>(47.1 , 81.2)	-8.8<br>(-32.4 , 23.4)
**Central Sub-Saharan Africa**	17907<br>(10158 , 26224)	83.9<br>(46.2 , 123.6)	33208<br>(21205 , 48532)	65.1<br>(40.9 , 95.8)	-22.4<br>(-40.9 , 5.9)
**Angola**	2722<br>(1693 , 5119)	72.7<br>(43.5 , 137)	6864<br>(4695 , 11876)	64.9<br>(44.2 , 114.1)	-10.7<br>(-33.9 , 30.9)
**Central African Republic**	870<br>(616 , 1208)	75.2<br>(51.9 , 100.4)	1260<br>(857 , 1798)	58.7<br>(40.3 , 79.9)	-21.9<br>(-40.9 , 2.9)
**Congo**	958<br>(704 , 1262)	92.4<br>(67.5 , 124.5)	1904<br>(1358 , 2882)	76.7<br>(55.2 , 115.4)	-16.9<br>(-40.3 , 14.8)
**Democratic Republic of the Congo**	12675<br>(6492 , 18938)	86.7<br>(42.9 , 130.2)	21940<br>(12866 , 31462)	63.8<br>(36.6 , 91.9)	-26.4<br>(-46.5 , 5.4)
**Equatorial Guinea**	121<br>(74 , 173)	61.8<br>(37.9 , 87.1)	306<br>(197 , 442)	68<br>(45.1 , 94.7)	10<br>(-27 , 78.9)
**Gabon**	561<br>(353 , 972)	101.5<br>(63.8 , 176.2)	934<br>(618 , 1703)	92.6<br>(62.5 , 167.8)	-8.8<br>(-38.2 , 35.4)
