# Supplementary figures and images for: Global, regional and national burden of bladder cancer and its attributable risk factors in 204 countries and territories, 1990–2019: a systematic analysis for the Global Burden of Disease study 2019
Source: BMJ Glob Health. 2021 Nov 29;6(11):e004128. doi: 10.1136/bmjgh-2020-004128 (PMC8634015; doi:10.1136/bmjgh-2020-004128)

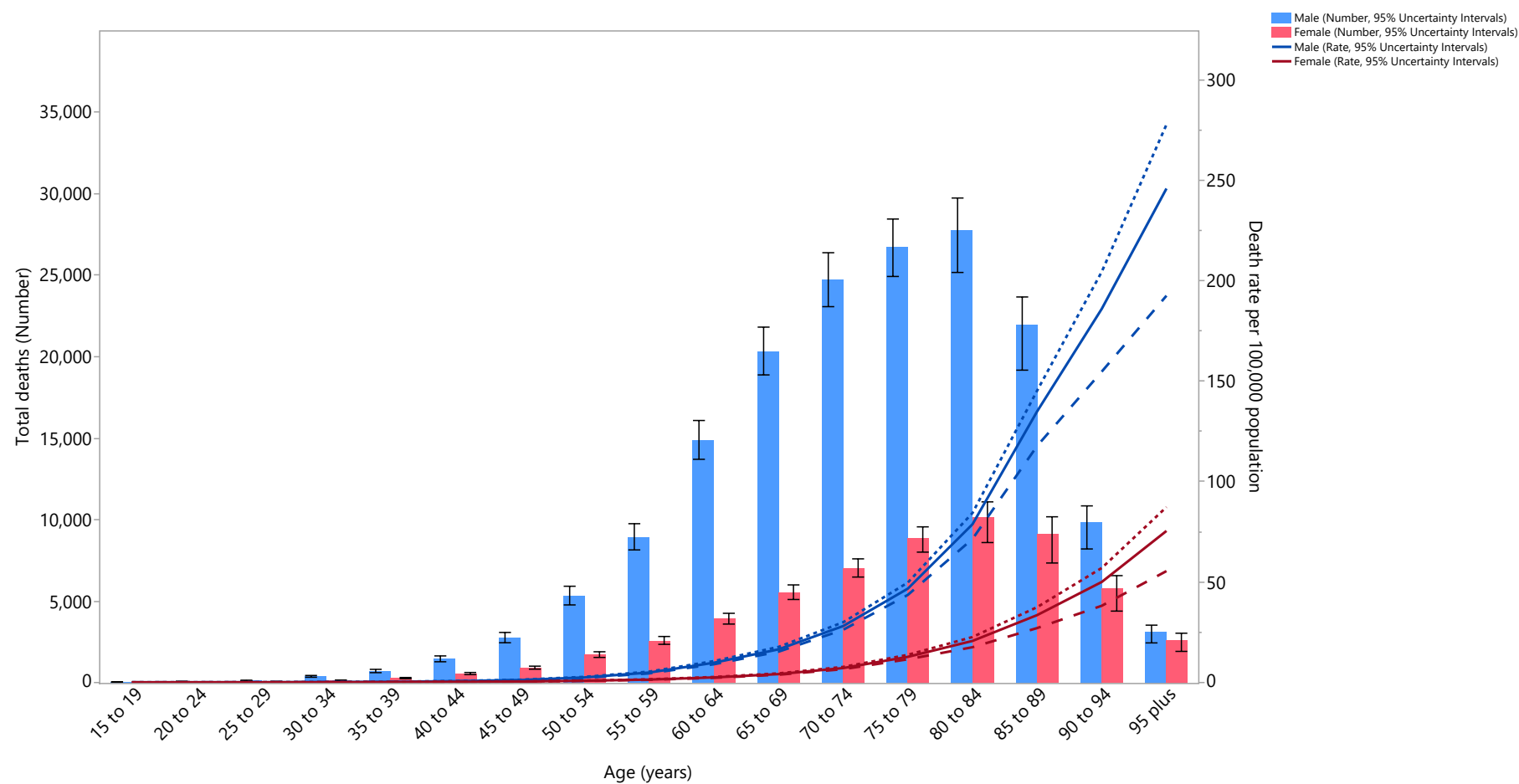

Supplement: Supplementary data [file bmjgh-2020-004128supp006.pdf]

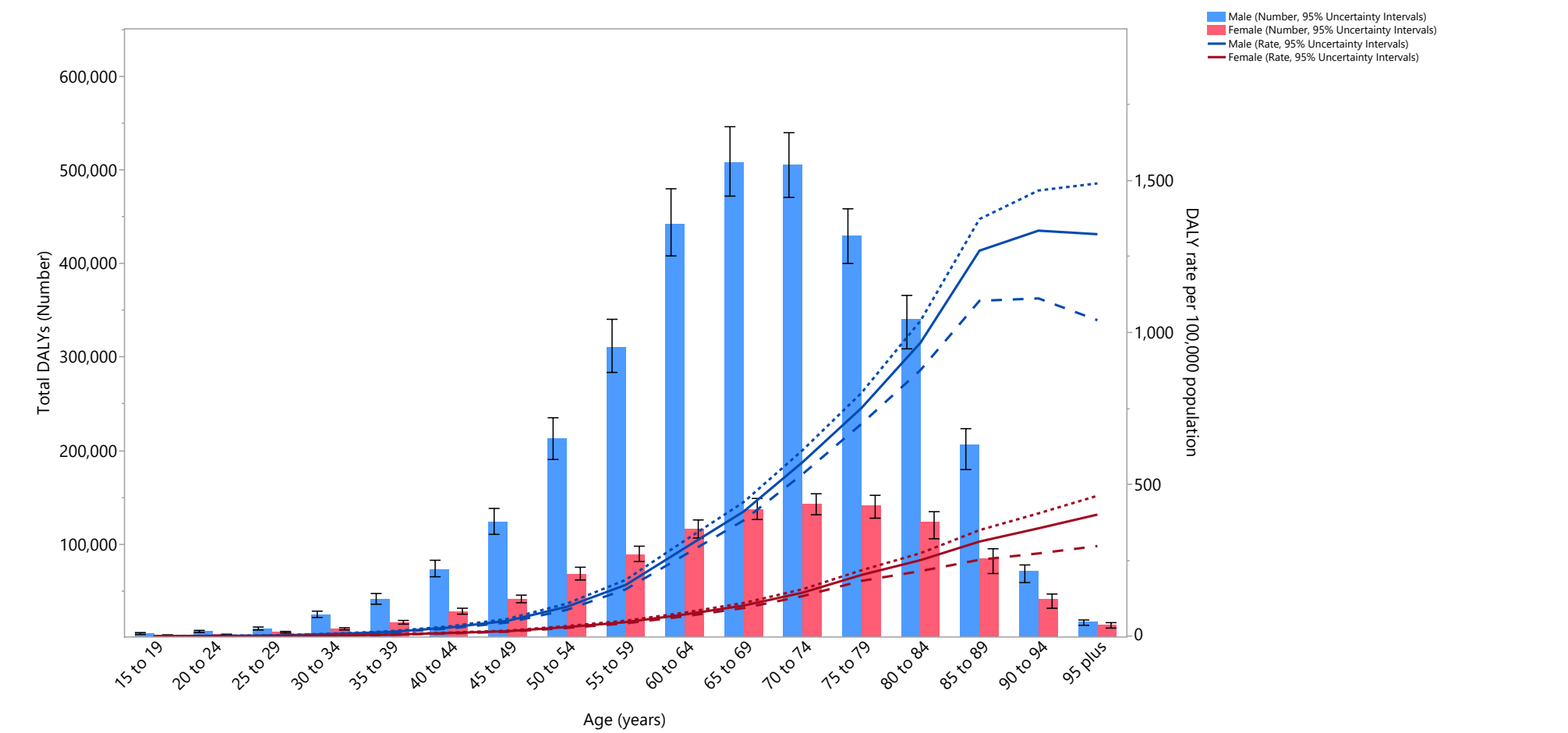

Supplement: Supplementary data [file bmjgh-2020-004128supp007.pdf]

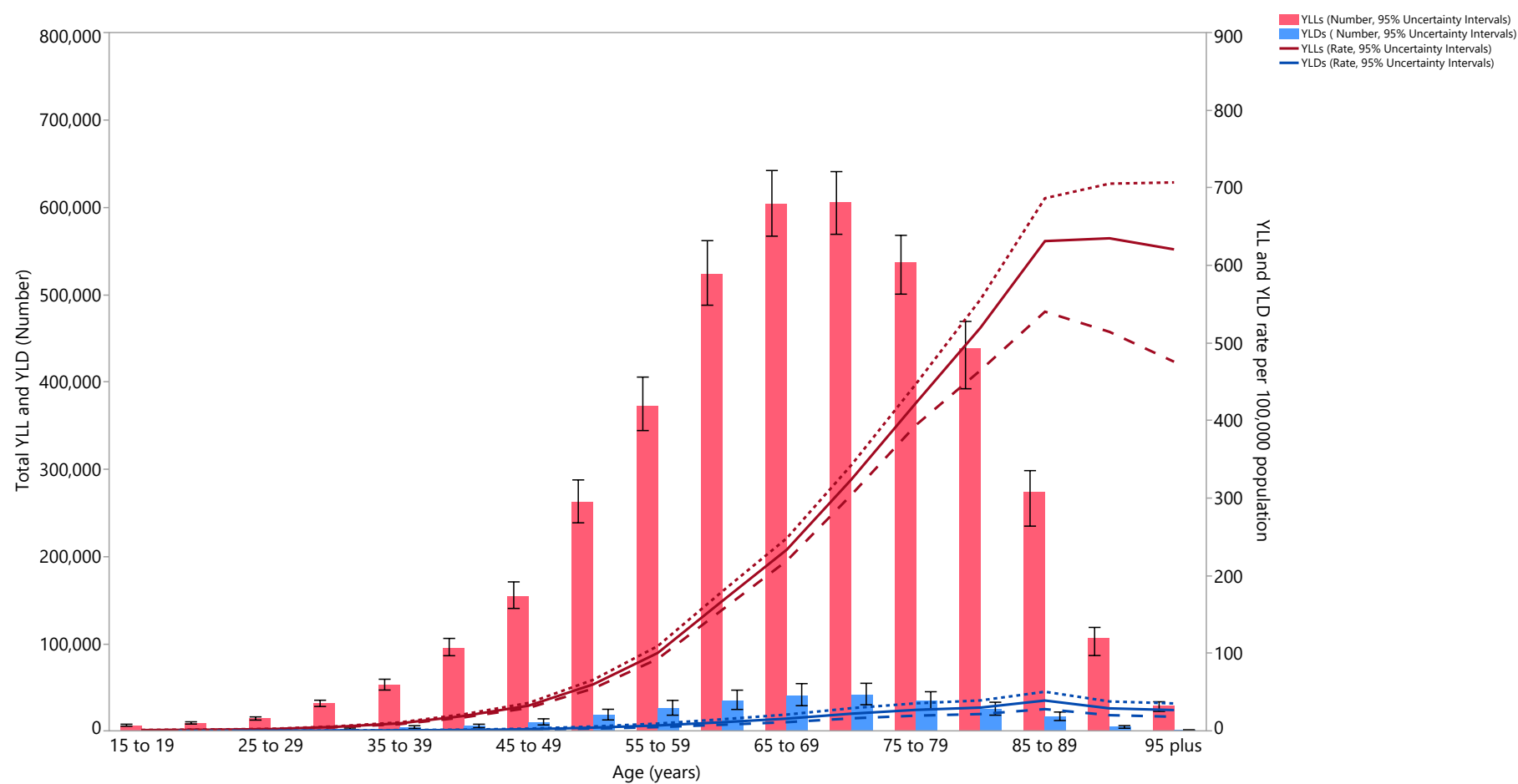

Supplement: Supplementary data [file bmjgh-2020-004128supp008.pdf]
