# Supplementary material for: Global, regional and national burden of bladder cancer and its attributable risk factors in 204 countries and territories, 1990–2019: a systematic analysis for the Global Burden of Disease study 2019
Source: BMJ Glob Health. 2021 Nov 29;6(11):e004128. doi: 10.1136/bmjgh-2020-004128 (PMC8634015; doi:10.1136/bmjgh-2020-004128)
Supplement: Supplementary data [file bmjgh-2020-004128supp009.pdf]

Authors' Contributions

Managing the estimation or publications process

Saeid Safiri and Mohsen Naghavi.

Writing the first draft of the manuscript

Saeid Safiri, Ali-Asghar Kolahi, Seyed Aria Nejadghaderi, Mark J M Sullman, Mahdi Sepidarkish, and Ahad Ashrafi-Asgarabad.

Primary responsibility for applying analytical methods to produce estimates

Saeid Safiri and Mohsen Naghavi.

Primary responsibility for seeking, cataloguing, extracting, or cleaning data; designing or coding figures and tables

Saeid Safiri, Mohammad Ali Mansournia, Mahdi Sepidarkish, and Ahad Ashrafi-Asgarabad.

Providing data or critical feedback on data sources

Mohammad Ali Mansournia, Amir Almasi-Hashiani, Amir Abdoli, Shailesh M Advani, Fares Alahdab, Vahid Alipour, Erfan Amini, Jalal Arabloo, Atif Amin Baig, Akshaya Srikanth Bhagavathula, Dejana Braithwaite, Florentino Luciano Caetano dos Santos, Dinh-Toi Chu, Vera Marisa Costa, Lalit Dandona, Rakhi Dandona, Meseret Derbew Molla, Mostafa Dianatinasab, Babak Eshrati, Farshad Farzadfar, Berhanu Elfu Feleke, Artem Alekseevich Fomenkov, Mansour Ghafourifard, Ahmad Ghashghaee, Mahaveer Golechha, Kebebe Bekele Gonfa, Nima Hafezi-Nejad, Soheil Hassanipour, Mowafa Househ, Segun Emmanuel Ibitoye, Farahnaz Joukar, G Anil Kumar, Reza Malekzadeh, Bartosz Miazgowski, Irmina Maria Michalek, Masoud Moghadaszadeh, Abdollah Mohammadian-Hafshejani, Reza Mohammadpourhodki, Shafiu Mohammed, Ali H Mokdad, Mariam Molokhia, Lorenzo Monasta, Mukhammad David Naimzada, Cuong Tat Nguyen, Huong Lan Thi Nguyen, Rajan Nikbakhsh, Mohammad Reza Nowroozi, Stanislav S Otstavnov, Hai Quang Pham, Majid Pirestani, Navid Rabiee, Alireza Rafiei, David Laith Rawaf, Salman Rawaf, Nima Rezaei, Nicholas L S Roberts, Gholamreza Roshandel, Abdallah M Samy, Sadaf G Sepanlou, Masood Ali Shaikh, Sara Sheikhbahaei, Jasvinder A Singh, Yonas Getaye Tefera, Mariya Vladimirovna Titova, Roman Topor-Madry, Bach Xuan Tran, Vesna Zadnik, Zhi-Jiang Zhang, and Mohsen Naghavi.

Developing methods or computational machinery

Erfan Amini, Xiaochen Dai, Mostafa Dianatinasab, Berhanu Elfu Feleke, James D Harvey, Simon I Hay, Mowafa Househ, Masoud Moghadaszadeh, Shafiu Mohammed, Ali H Mokdad, Alireza Rafiei, Nicholas L S Roberts, Abdallah M Samy, Rixing Xu, and Mohsen Naghavi.

Providing critical feedback on methods or results

Seyed Aria Nejadghaderi, Mohammad Ali Mansournia, Mark J M Sullman, Amir Almasi-Hashiani, Shailesh M Advani, Fares Alahdab, Vahid Alipour, Erfan Amini, Etsay Woldu Anbesu, Jalal Arabloo, Atalel Fentahun Awedew, Atif Amin Baig, Akshaya Srikanth Bhagavathula, Ali Bijani, Antonio Biondi, Dejana Braithwaite, Florentino Luciano Caetano dos Santos, Dinh-Toi Chu, Vera
